# Agreement between dried blood spots and HemoCue in Tamil Nadu, India

**DOI:** 10.1038/s41598-021-88425-y

**Published:** 2021-04-29

**Authors:** Reshma P. Roshania, Rukshan V. Mehta, Ashwini Shete, Rohini Bingewar, Sangeeta Kulkarni, Aprajit Mahajan, Grant Miller, Alessandro Tarozzi, Reynaldo Martorell

**Affiliations:** 1 Nutrition and Health Sciences, Laney Graduate School, Emory University, Atlanta, GA USA; 2 National AIDS Research Institute, Indian Council of Medical Research, Pune, Maharashtra India; 3 Department of Agricultural and Resource Economics, University of California Berkeley, Berkeley, CA USA; 4 Center for Health Policy/Center for Primary Care and Outcomes Research, School of Medicine, Stanford, CA USA; 5 Department of Economics and Business, Universitat Pompeu Fabra and Barcelona GSE, Barcelona, Spain; 6 Hubert Department of Global Health, Rollins School of Public Health, Emory University, 1518 Clifton Road, NE, CNR 5005, Mailstop #1518-002-7BB, Atlanta, GA 30322 USA

**Keywords:** Blood proteins, Enzymes, Anaemia

## Abstract

India retains the world’s largest burden of anemia despite decades of economic growth and anemia prevention programming. Accurate screening and estimates of anemia prevalence are critical for successful anemia control. Evidence is mixed on the performance of HemoCue, a point-of-care testing device most widely used for large-scale surveys. The use of dried blood spots (DBS) to assess hemoglobin (Hb) concentration is a potential alternative, particularly in field settings. The objective of this study is to assess Hb measurement agreement between capillary HemoCue and DBS among two age groups, children 6–59 months and females age 12–40 years. We analyzed data from the baseline round of a cluster randomized rice fortification intervention in Cuddalore district of Tamil Nadu, India. Capillary blood was collected from a subset of participants for Hb assessment by HemoCue 301 and DBS methods. We calculated Lin’s concordance correlation coefficient, and tested bias by conducting paired t-tests of Hb concentration. Independence of the bias and Hb magnitude was examined visually using Bland–Altman plots and statistically tested by Pearson’s correlation. We assessed differences in anemia classification using McNemar’s test of marginal homogeneity. Concordance between HemoCue and DBS Hb measures was moderate for both children 6–59 months (*ρ*_*c*_ = 0.67; 95% CI 0.65, 0.71) and females 12–40 years (*ρ*_*c*_ = 0.67: 95% CI 0.64, 0.69). HemoCue measures were on average 0.06 g/dL higher than DBS for children (95% CI 0.002, 0.12; p = 0.043) and 0.29 g/dL lower than DBS for females (95% CI − 0.34, − 0.23; p < 0.0001). 50% and 56% of children were classified as anemic according to HemoCue and DBS, respectively (p < 0.0001). 55% and 47% of females were classified as anemic according to HemoCue and DBS, respectively (p < 0.0001). There is moderate statistical agreement of Hb concentration between HemoCue and DBS for both age groups. The choice of Hb assessment method has important implications for individual anemia diagnosis and population prevalence estimates. Further research is required to understand factors that influence the accuracy and reliability of DBS as a methodology for Hb assessment.

## Introduction

The consequences of anemia, defined as low hemoglobin (Hb) concentration, are significant and many, including poor maternal and perinatal outcomes such as maternal mortality, low birth weight and pre-term birth; longer term child health outcomes such as impaired cognitive development; and broader socioeconomic costs due to reduced work potential^1^. India has a severe burden of anemia; the prevalence of anemia among children under five is approximately 40%^2^, and over one in two women of reproductive age (15–49 years) are anemic^3^.

Despite longstanding anemia prevention programming and recent decades of economic growth, there has been little progress in reducing the prevalence of anemia in India^4^, with trends in some states showing increases in anemia burden^5^. A key component of successful anemia control is accurate diagnosis. The gold standard for the assessment of Hb concentration is analysis of venous blood using the direct cyanmethemoglobin (CMG) method^6^. This method, however, is not always feasible for survey research as venous blood collection is invasive, must be conducted by skilled personnel, and spectrophotometric analysis in a laboratory is required within hours of specimen collection.

HemoCue, a portable, point-of-care testing device that reads Hb in whole capillary, venous or arterial blood by spectrophotometry is widely used for anemia prevalence estimation in population-based surveys. In field settings, collection of capillary blood is most feasible, although biological variability between capillary and venous Hb concentration is likely^7^. Existing evidence demonstrates that accuracy of HemoCue capillary blood samples is generally poorer compared to the accuracy of venous and arterial HemoCue samples^8^. One review of HemoCue accuracy among children concluded that research is lacking in field-based and primary care settings in low resource contexts^9^. Furthermore, HemoCue performance can vary by specific model based on preanalytical factors related to the context such as temperature and humidity, as well as factors related to measurement protocol such as time from specimen collection to reading^10^. Adherence to quality control procedures during data collection such as using microcuvettes from newly opened containers and ensuring complete microcuvette filling are also critical for accurate HemoCue readings^11^.

An alternate field-friendly method of Hb collection and assessment is indirect CMG using dried blood spots (DBS), for which capillary blood is collected on filter paper, dried, and transported to a laboratory where samples are extracted with a solvent before analysis. There are several advantages to this approach compared to the gold standard^12^. Similar to HemoCue, sampling is less invasive and equipment is portable; additionally, DBS is less costly than HemoCue and samples can remain at room temperature for several days before analysis. Unlike HemoCue however, Hb measures from DBS are not immediately available and must be analyzed in a laboratory. We found only one study comparing indirect CMG using DBS of capillary blood to the gold standard of direct CMG of venous blood, which reported that the indirect method overestimates anemia prevalence. Hb measures using DBS were on average 0.7 g/dL lower than direct CMG^13^. This study also compared indirect CMG using DBS to HemoCue and found that DBS measures were on average, 0.9 g/dL lower than for HemoCue, increasing anemia prevalence by 24 percentage points, from 14% by HemoCue to 38% by DBS. The authors suggest incomplete dissolution of blood from filter paper as a potential explanation of these findings. This conclusion has been challenged by Mohanram and colleagues however, who report a similar pattern despite ensuring complete dissolution of blood from DBS samples; capillary Hb values measured using DBS were approximately 2.0 g/dL lower than HemoCue for both pregnant and non-pregnant women in a hospital setting^14^. Lower DBS readings compared to HemoCue have also been reported for venous blood comparisons; a hospital-based study among adults in rural India found that Hb concentration values from indirect CMG using DBS of venous blood are 1.8 g/dL lower compared to venous HemoCue^15^.

The comparisons between HemoCue and indirect CMG using DBS have only been examined in adult populations. Differences in Hb measurement concordance by age group have been observed when comparing HemoCue to a reference method^16^, suggesting that age may be an important consideration in the choice and assessment of Hb measurement methods. The objective of this study is to evaluate the extent of agreement between two methods of Hb estimation that are feasible in field settings, capillary HemoCue and indirect CMG using DBS, among different age groups that are critical to consider to prevent the long-term and intergenerational consequences of anemia—young children, and adolescent and adult non-pregnant females.

## Methods

This cross-sectional study analyzed the baseline round of data from a cluster randomized rice fortification intervention in Tamil Nadu, India. The research project (clinicaltrials.gov, NCT 03573570) is jointly funded by the Government of Tamil Nadu, Global Innovation Fund, the Tata Trusts and King Philanthropies; led by investigators at Stanford University, University of California Berkeley, Universitat Pompeu Fabra, and Emory University; and carried out in collaboration with the Abdul Latif Jameel Poverty Action Lab (JPAL) South Asia at the Institute of Financial Management and Research (IFMR). All methods were performed in accordance with the approved protocol and relevant regulations.

### Study population

Baseline data were collected between July and October 2018 in the catchment areas of 223 randomly selected Fair Price Shops (FPS) across predominantly rural Chidambaram and Bhuvanagiri taluks (administrative subdivisions) in Cuddalore District. The Government of India Public Distribution System operates through FPS by providing a monthly quota of subsidized staples; in Tamil Nadu this includes rice, wheat and sugar to households with ration cards. Selected FPS were randomized to receive either nutrient fortified or regular rice. Within each FPS area, households with active FPS ration cards were sampled randomly until a target of 40 non-pregnant females 12–40 years and 40 children 6–59 months were reached. In the overall study, a total of 7320 eligible households were surveyed, containing 6646 children 6–59 months and 8076 females 12–40 years. Both HemoCue and DBS samples were available for a random subset of all study participants (ten females 12–40 years and ten children 6–59 months per FPS area); this subset was included in our analysis, further described below.

### Review of literature

We searched PubMed and Google Scholar for published studies on the validity of HemoCue and DBS methods as compared to the gold standard of direct CMG, and studies comparing HemoCue with DBS of both capillary and venous blood. We reviewed studies in resource-limited contexts, from both community and hospital settings.

### Data collection and Hb assessment

HemoCue and DBS were collected at a fixed location, a health camp set up by the survey team in each FPS area. After completion of consenting protocols and a household survey questionnaire, participants visited the health camp for biomarker and anthropometric data collection. The questionnaires developed and used in the study are listed as supplemental material S1. Samples were collected first for HemoCue followed by DBS. A diagnosis of anemia was made based on Hb concentration cut-offs of < 11.0 g/dL for children 6–59 months and < 12.0 g/dL for females 12–40 years^17^.

Hb measures were collected for the entire sample using HemoCue Hb 301 System using standard protocols developed for use in field settings^11^. Ten microliters of capillary blood were collected from each participant using disposable microcuvettes. For the subset of participants for whom DBS samples were also available, an additional five drops of capillary blood from the same puncture site were collected.. Capillary blood was collected by finger prick, and for children 6–12 months, by heel prick.

DBS samples were collected on Whatman 903 filter paper, which were then dried, stored in sealed plastic bags with desiccant, and transported to the National AIDS Research Institute (NARI) in Pune, located in the state of Maharashtra. There, DBS samples were stored at − 20° centigrade and analyzed using the colorimetric indirect CMG method after elution in Drabkin’s solution. A punch of 3.2 mm in diameter was cut from a single spot and eluted in Drabkin’s solution for two hours on a shaker. Hb concentration was estimated in the eluent by measuring optical density on a spectrophotometer (Tecan Trading AG, Switzerland). Hb standards were included with every batch of samples and the standard curve with R^2^ value of > 0.95 was considered acceptable. Low and normal level controls prepared from whole blood were also included in each batch of samples for monitoring internal quality control of the test. Method validation conducted prior to the study did not demonstrate volcano effect.

Enumeration teams underwent extensive training prior to the start of data collection. Practice exercises were conducted as per recommended training protocols^11^. Following practice, standardization exercises were conducted to help identify systematic errors in implementation of protocols for blood sample collection and with HemoCue machines. Interobserver and intra-observer correlation measures < 0.7 were flagged as problematic, and further training and practice was advised for data collectors accordingly.

### Sample selection for analysis

**Figure 1:**
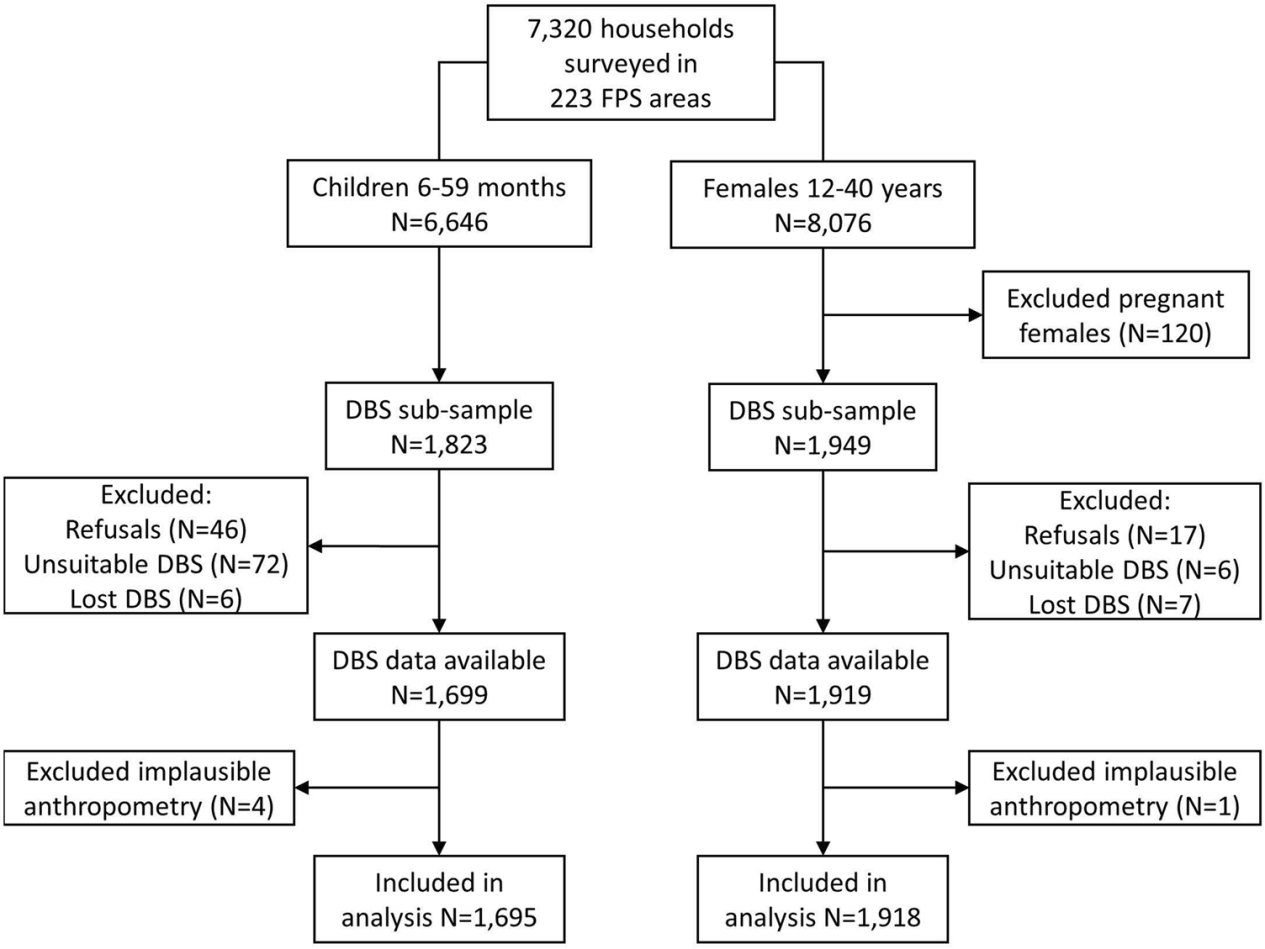
Sample selection flowchart for analysis of HemoCue and DBS Hb measurement agreement.

We included cases with both HemoCue and complete DBS samples available for children (n = 1695) and non-pregnant females (n = 1918), and excluded cases with implausible anthropometric data, as described in Fig. 1. DBS samples were considered unsuitable for analysis if the sample exhibited a serum ring, or if there was an insufficient quantity of blood (< 75% of the spot was filled).

### Statistical analysis

We first generated descriptive statistics of our sample to characterize sociodemographic characteristics as well as nutritional status indicated by height-for-age (HAZ) and weight-for-height z-score (WHZ)^18^ among children, body mass index-for-age z-score (BFA)^19^ among adolescent girls 12–19 years of age, and body mass index (BMI) among adult women 20–40 years of age. To test for systematic bias due to differences among 21 HemoCue machines used for the study, we conducted analysis of variance followed by Tukey and Scheffe pairwise comparisons.

We conducted paired t-tests of the difference of the two measures; a significant, non-zero difference between the means implies systematic bias. To explore the extent and patterns of agreement, we created Bland–Altman diagrams^20^, with 95% statistical limits of agreement (LoA), represented by ± 1.96 standard deviations (SD) of the mean. For normally distributed difference values, it is expected that 95% of difference values would fall within these limits. It is important to note that the clinical LoA can be different from these and should be established prior to the analysis of a study^21^. We conducted a Pearson’s correlation of the difference and mean of the HemoCue and DBS measures for statistical assessment of any patterns observed in the Bland–Altman plots; a significant correlation implies a dependence between the bias (difference of measures) and magnitude (mean of measures)^22^. We assessed concordance between HemoCue and DBS measures by calculating Lin’s concordance correlation coefficient (CCC)^23^. Lin’s CCC (*ρ*_*c*_) is the appropriate test for agreement between two measures^24^ as it specifically considers how well the data fit around the 45-degree line of equality. CCC values ranging from 0.7 to 1.0 are considered good agreement, and values below 0.4 are considered poor agreement^25^.

For assessment of the binary outcome of interest (anemic/non-anemic), we tested whether the proportion of the samples who are classified as anemic differ significantly by method of measurement by conducting McNemar’s test of marginal homogeneity, a modification of the ordinary chi square test. Analyses were conducted separately for children 6–59 months (n = 1695), adolescent non-pregnant females 12–19 years of age (n = 93), and adult non-pregnant females 20–40 years of age (n = 1825). Since there were no differences observed between adolescent and adult females for our primary outcomes of Hb concentration and anemia prevalence (data not shown), we present these as combined results for all non-pregnant females 12–40 years of age. We conducted analyses in SAS 9.4, accounting for the multi-stage clustering survey design for descriptive statistics. Statistical significance was set at α = 0.05.

### Ethics approval and consent to participate

Ethical review was conducted by Institutional Review Boards in India (IFMR) and the USA (Stanford University); the study was approved by the Indian Council of Medical Research (ICMR). Written informed consent was obtained from research participants. For children, written informed consent was obtained from caregivers. Fingerprints were obtained from research participants and caregivers who could not write their signature.

## Results

Children who did not participate due to either refusal or unsuitable DBS samples were on average younger (9 months and 15 months, respectively) than children who successfully participated. Children for whom unsuitable DBS samples were collected had 0.5 g/dL lower Hb values as measured by HemoCue, on average, compared to those children for whom valid DBS samples were collected for DBS analysis. There were no differences in mean age or Hb values as measured by HemoCue among females 12–40 years between successful participation and refusals or unsuitable DBS samples.

### Study population characteristics

Over 99% of sampled females 12–40 years belonged to a marginalized community (Scheduled Caste/Scheduled Tribe or Backward Class), and close to three-quarters completed secondary schooling or higher (Table 1). Among adolescent girls, 16.1% were thin and 17.2% were classified as overweight/obese. Among adult non-pregnant women, 14.4% were underweight and 36.8% were overweight or obese.

**Table 1:** Sociodemographic characteristics and nutrition status of females 12–40 years (n = 1918).

	n	%
**Taluk**		
Bhuvanagiri	745	38.8
Chidambaram	1173	61.2
**Age category**		
< 15 years	36	1.9
15–19 years	57	3.0
20–24 years	329	17.2
25–29 years	790	41.2
30–34 years	517	26.9
≥ 35 years	189	9.9
**Education level**		
None	48	2.5
Primary	466	24.3
Secondary	635	33.1
Higher	769	40.1
**Religion**		
Hindu	1855	96.7
Muslim	28	1.5
Christian	35	1.8
**Caste**		
General	3	0.2
SC/ST^a^	874	45.7
Backward classes	1037	54.2
**BFA (12–19 years)**^**b**^		
Thin	15	16.1
Normal	62	66.7
Overweight/obese	16	17.2
**BMI category (20–40 years)**^**c**^		
Underweight	262	14.4
Normal	890	48.8
Overweight	493	27,0
Obese	178	9.8
**Hb concentration, g/dL**	**Mean**	**SD**
HemoCue	11.64	1.42
DBS	11.93	1.71

^a^*SC/ST* scheduled caste/scheduled tribe.

^b^Body mass index-for-age z-score (BFA): n = 93, thin: <  − 2 BFA; normal: − 2 to ≤ 1 BFA; overweight/obese: > 1 BFA.

^c^Body mass index (BMI): n = 1823, underweight: < 18.5 kg/m^2^; normal: 18.5 to < 25 kg/m^2^; overweight: 25 to < 30 kg/m^2^; Obese: ≥ 30 kg/m^2^.

Our sample had slightly more male children than female children (Table 2). The prevalence of stunting was 22.8%, and wasting was 13.6%.

**Table 2:** Age, sex, and nutrition status of children 6–59 months (n = 1695).

	n	%
**Age category**		
6–11 months	117	6.9
12–17 months	189	11.2
18–23 months	169	10.0
24–29 months	185	10.9
30–35 months	178	10.5
36–41 months	208	12.3
42–47 months	178	10.5
48–53 months	233	13.7
54–59 months	238	14.0
**Sex**		
Male	917	54.1
Female	777	45.8
**Stunting**^a^		
Severely stunted	78	4.6
Moderately stunted	306	18.2
Not stunted	1297	77.2
**Wasting**^b^		
Severely wasted	22	1.3
Moderately wasted	206	12.3
Not wasted	1449	86.3
**Hb concentration, g/dL**	**Mean**	**SD**
HemoCue	10.79	1.34
DBS	10.72	1.70

^a^Severely stunted: <  − 3 HAZ; moderately stunted: − 3 to < − 2 HAZ; not stunted: ≥  − 2 HAZ.

^b^Severely wasted: <  − 3 WHZ; moderately wasted: − 3 to < − 2 WHZ; not wasted: ≥  − 2 WHZ.

### Measurement agreement

We did not observe systematic differences among HemoCue machines. Among children, mean (± SD) Hb concentration was 10.79 ± 1.34 g/dL and 10.72 ± 1.70 g/dL by HemoCue and DBS, respectively. Among females 12–40 years, mean Hb (± SD) concentration was 11.64 ± 1.42 g/dL and 11.93 ± 1.71 g/dL by HemoCue and DBS, respectively. Paired t-tests between the two measures indicate the mean differences were significantly different from zero for both age groups (Table 3), implying bias. Concordance between HemoCue and DBS was 0.67 for both groups, implying moderate agreement according to the cut-offs we used; alternate cut-offs have been proposed, further described in the discussion section.

**Table 3:** Measures of agreement between HemoCue and DBS, children 6–59 months and females 12–40 years.

	Mean difference (g/dL)		Range	Paired t-test		Concordance	
	HemoCue-DBS	(95% CI)	(min, max)	t	p-value	ρ_c_	(95% CI)
Children 6–59 months	0.06	(0.002, 0.12)	(− 7.49, 4.51)	2.02	0.043	0.67	(0.65, 0.71)
Females 12–40 years	− 0.29	(− 0.34, − 0.23)	(− 6.48, 6.59)	− 9.91	< 0.0001	0.67	(0.64, 0.69)

*CI* confidence interval.

**Figure 2:**
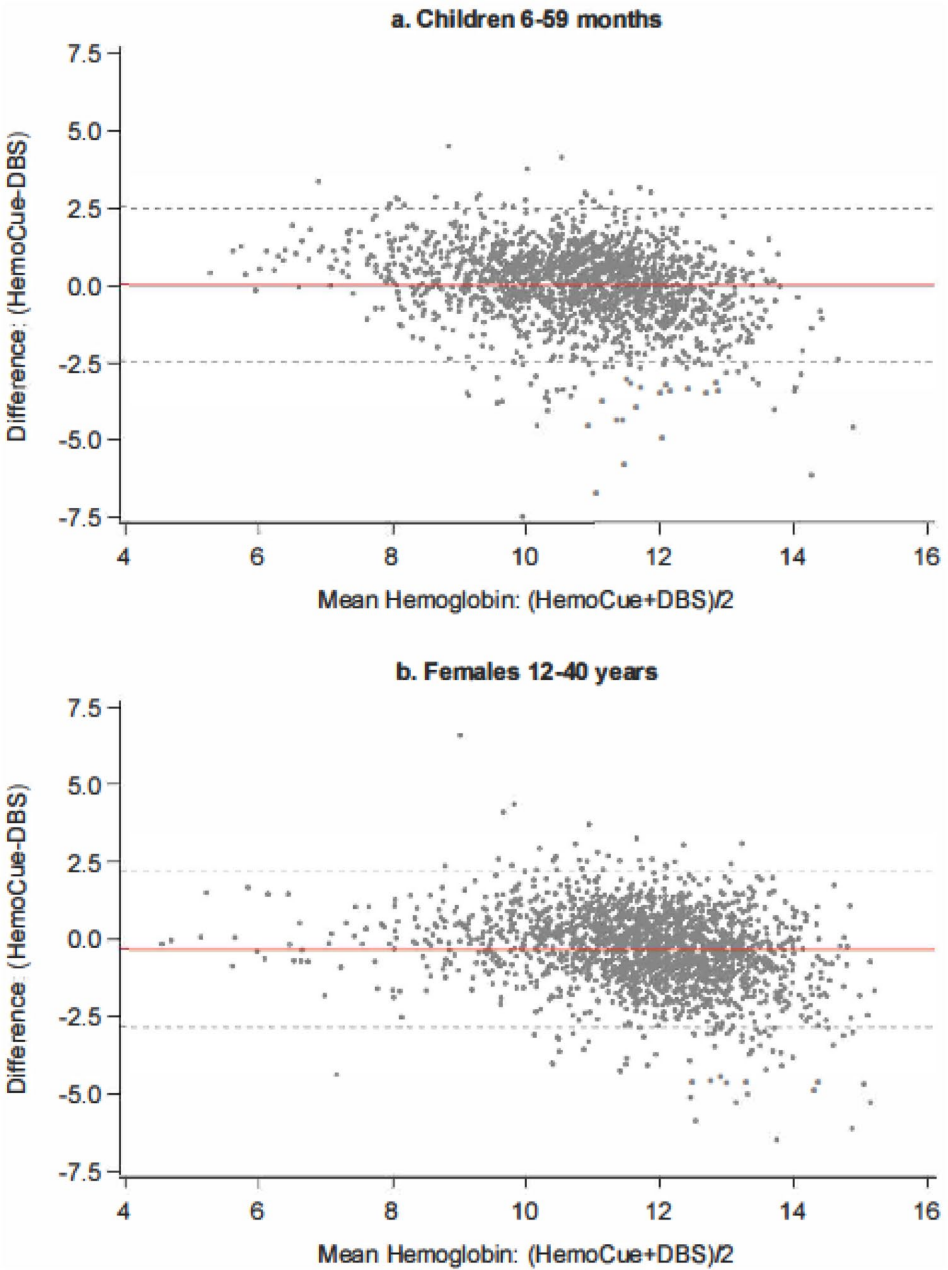
Bland–Altman plot of HemoCue and DBS Hb measures, children 6–59 months (**a**) and females 12–40 years (**b**). The red line represents the mean difference; the dashed grey lines represent ± 1.96 SD of the mean difference indicating the statistical limits.

Results from the analysis of the Bland–Altman plots (Fig. 2a,b) demonstrate that 95.1% of the difference values lay within the statistical LoA for both age groups as expected for normally distributed data. For children, the LoA were from − 2.4 to 2.6 g/dL; 23 of 1695 or 1.4% of values were above the upper limit and 60 or 3.5% of the values were below the lower limit. For females 12–40 years, the LoA were from − 2.8 to 2.2 g/dL; 32 of 1918 or 1.7% of values were above the upper limit and 62 or 3.2% of values were below the lower limit. Examination of the plots also shows that for both groups, as mean Hb concentration increases, the difference between HemoCue and DBS measures shifts from positive to negative values. The difference between HemoCue and DBS has a weak, negative correlation with the mean of both measures for children (r = − 0.28, p < 0.0001) and females 12–40 years (r = − 0.25, p < 0.0001). That is, the difference between the measures depends on the magnitude of the measure.

To explore this further, we present the average difference between HemoCue and DBS by Hb concentration interval, shown in Table 4. Among children, mean difference values at the lower Hb concentration intervals tend to be positive and decrease to negative values at higher Hb concentration intervals; this pattern is not as apparent among females 12–40 years. For both groups, variability in the differences increases with increasing magnitude.

**Table 4:** Mean differences by Hb concentration interval, children 6–59 months and females 12–40 years.

Hb interval^a^	Children 6–59 months		Females 12–40 years	
	n	Mean difference^b^ ± SD	n	Mean difference^b^ ± SD
< 8 g/dL	66	0.98 ± 0.80	33	− 0.06 ± 1.14
8 to < 10 g/dL	402	0.35 ± 1.18	160	0.29 ± 1.19
10 to < 12 g/dL	912	0.08 ± 1.21	805	− 0.09 ± 1.17
≥ 12 g/dL	315	− 0.55 ± 1.28	920	− 0.57 ± 1.29

^a^Average of DBS and HemoCue measures.

^b^HemoCue—DBS.

### Anemia classification

**Figure 3:**
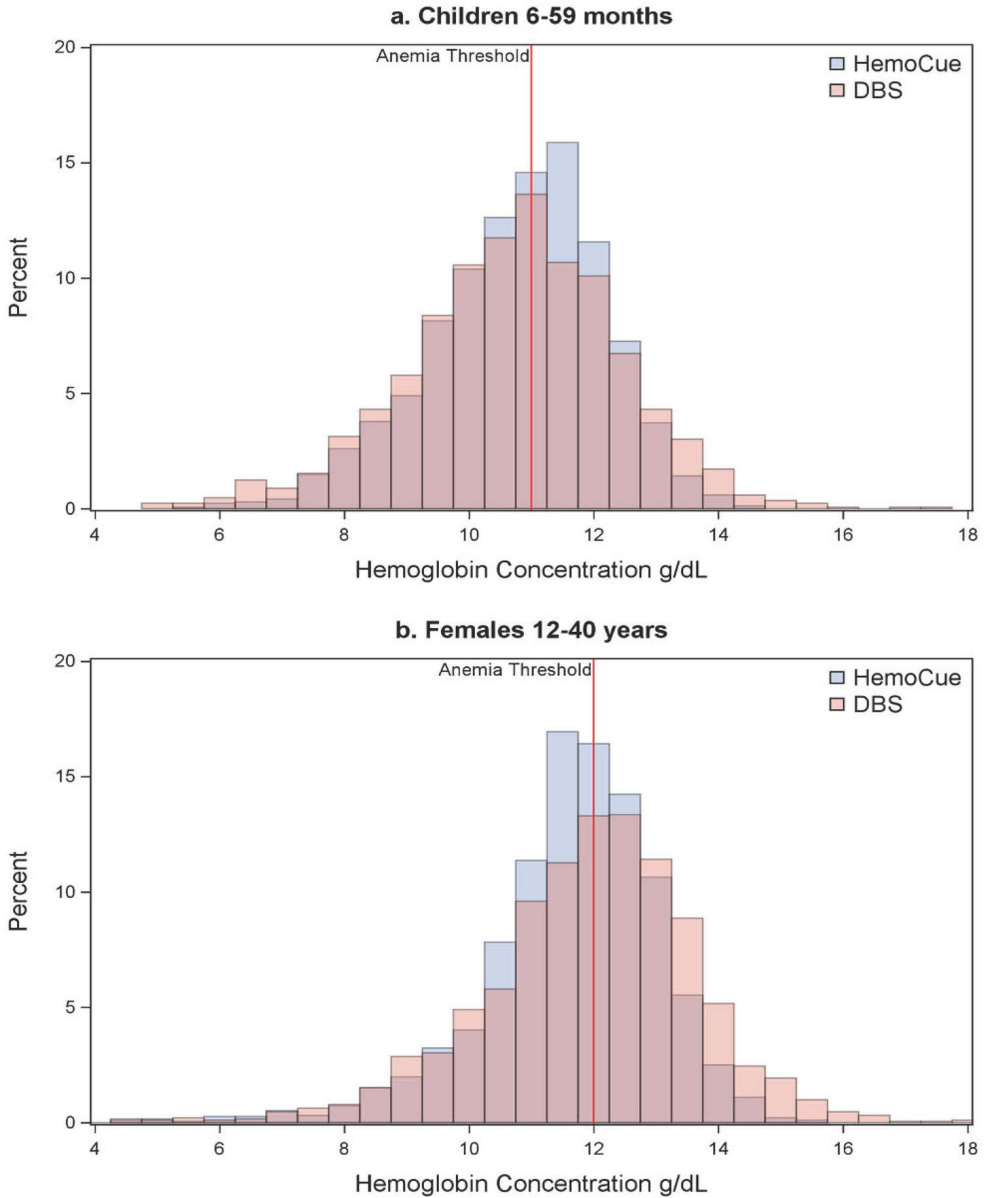
Overlaid histograms for Hb (g/dL) assessed by HemoCue and DBS for (**a**) children 6–59 months and (**b**) females 12–49 years, with the threshold for anemia shown in red.

Among children, 50% and 56% classified as anemic according to HemoCue and DBS, respectively (Table 5). Among females 12–40 years, 55% classified as anemic according to HemoCue and 47% classified as anemic according to DBS. For both groups, the null hypothesis of marginal homogeneity was rejected (McNemar’s χ^2^: 42.5, p < 0.0001 and McNemar’s χ^2^: 18.5, p < 0.0001, respectively), indicating that the proportion of positive cases from the two methods are significantly different, more so in females 12–40 years than in children. In Fig. 3 we show overlaid histograms of HemoCue and DBS Hb concentration, as well as the anemia threshold, to illustrate the different patterns in children 6–59 months and females 12–40 years. In the latter group, the HemoCue values distribution is shifted to the *left* of the anemia threshold compared to the shift to the *righ*t of the threshold for DBS values. In children, there is greater overlap in the distributions of HemoCue and DBS Hb values.

**Table 5:** Anemia classification^a^ by HemoCue and DBS, children 6–59 months and females 12–40 years.

	Children 6–59 months			Females 12–40 years		
	Anemia DBS, n	No Anemia DBS, n	Total, n (%)	Anemia DBS, n	No Anemia DBS, n	Total, n (%)
Anemia HemoCue, n	699	183	852 (50.3)	703	360	1063 (55.4)
No anemia HemoCue, n	275	568	843 (49.7)	205	650	855 (44.6)
Total n, (%)	944 (55.7)	751 (44.3)	1695 (100.0)	908 (47.3)	1010 (52.7)	1918 (100.0)

^a^Anemia is classified as Hb concentration < 11.0 g/dL for children and < 12.0 g/dL for females.

## Discussion

Around 50% of children 6–59 months and 55% of non-pregnant females 12–40 years in our study sample were diagnosed as anemic using the point-of-care device HemoCue; these prevalence estimates are consistent with national^3^ and regional^26^ data. Our results demonstrate that there is moderate statistical agreement between indirect CMG using DBS and HemoCue in capillary Hb assessment among both children 6–59 months and females 12–40 years of age. Concordance between the two measures was 0.67 for both groups, indicating moderate agreement according to criteria used in previous research^25^; however, McBride has proposed alternative cut-offs^27^, for which all CCC values below 0.90 are considered poor. Lin’s CCC is the appropriate method of assessing agreement as it provides the extent of fit around the line of perfect agreement, whereas Pearson’s correlation coefficient provides the extent of fit around any line^24^.

The bias observed between the two measures seems to operate inversely for children and females 12–40 years. HemoCue measures 0.06 g/dL higher than DBS, on average, for children and are 0.29 g/dL lower than DBS for females, on average. While these differences were significant, it is important to note that our study had a large sample size and was therefore powered to detect small differences. Indeed, the magnitude of difference is minor and arguably clinically insignificant, particularly among children. At the same time, the observed bias translates to significantly different anemia prevalence estimates at the population level given the distribution of Hb values around the age-specific anemia thresholds. Measures by HemoCue result in a lower anemia prevalence compared to DBS by over five percentage points for children, and 29% of children who are classified as anemic according to DBS would classify as normal using HemoCue. Conversely, measures by HemoCue result in a higher anemia prevalence compared to DBS by eight percentage points for females 12–40 years; around 34% who are classified as anemic according to HemoCue would classify as normal using DBS.

Overall, the differences by method we observed in both mean Hb measurements and anemia prevalence are lower than those reported by previous studies^13–15^; in contrast to our findings, these studies also observed lower Hb concentration by indirect CMG using DBS compared to HemoCue for capillary blood samples among adult women. Potential explanations include substantial variability of Hb in capillary blood depending on site of collection and over time for example^28^, differing climatic and environmental conditions, or varying specimen collection and quality control protocols^11^. Ours is the first study to compare these two methods among children.

The 95% LoA represent the limits within which 95% of normally distributed difference values are expected to lie; in our study, they range from − 2.4 to 2.6 g/dL and − 2.8 to 2.2 g/dL for children and females 12–40 years, respectively. In other words, for 95% children for example, one method of measurement would range from 2.4 less to 2.6 g/dL more than the other method of measurement^29^. These are statistically derived, however, and may be unacceptable for clinical method agreement. The threshold criteria for hemoglobin estimation evaluation established by the Clinical Laboratory Improvement Amendments^30^ is ± 7% of the reference method. We were unable to assess the percent difference with a reference method, mentioned in the limitations below; however, the mean measurements of the two methods we compared were within ± 7% of each other for both age groups. We also found the difference between HemoCue and DBS measures varies with Hb concentration magnitude, especially among children. Within this group, HemoCue measures appear higher than DBS at lower Hb concentration levels, and lower than DBS at higher Hb concentration levels, reinforcing our finding that HemoCue results in a lower anemia prevalence compared to DBS among children. Varying HemoCue performance by magnitude among children has been previously observed^16^. This might be partially explained by varying quality of blood samples. Blood collection among children is often challenging. In our study, over 90% of DBS samples that were unsuitable for analysis were among children. ‘Milking’, or application of pressure on the site of the puncture to stimulate blood flow should not be done since it results in hemodilution by increasing plasma and interstitial fluid in the sample, thereby lowering Hb concentration readings^28^. Although this process was discouraged during trainings, if ‘milking’ did inadvertently occur, we would expect Hb measures by DBS to be lower as we observed, since DBS collection occurred after HemoCue for all research participants. Varying the order of sample collection by method would have allowed testing of the effects of this potential source of measurement error; we recommend this to be done in future studies. However, we would have expected this trend across the Hb magnitude spectrum, not only at the lower end. Our study highlights the need for research on methodological validation over the Hb concentration range especially among children.

The choice of method of anemia assessment can have a substantial effect at the individual level with respect to appropriate anemia classification, referral and treatment, as well as at regional levels with respect to prevalence estimates and resource allocation. In India, national programs^31^ provide prophylactic iron and folic acid supplementation to children, adolescent boys and girls, women of reproductive age, and pregnant and lactating women. The country’s anemia reduction strategy also calls for periodic screening and testing for anemia in all age groups, and appropriate referral and treatment based on mild, moderate and severe anemia classification. Currently, anemia is screened at the community level using clinical pallor assessment^32^ and tested at the primary health clinic and hospital levels utilizing Sahli’s method, for which capillary blood is collected by pipette and mixed with hydrochloric acid and distilled water until the formed brown acid hematin matches the color of the standard. It is inexpensive and simple, but is subjective as the method is based on color comparison^33^. Studies assessing accuracy of Sahli’s method have found high sensitivity (84–92%) but low specificity (39–63%) compared to HemoCue^34,35^. Strategy documentation suggests that digital hemoglobinometers will be adopted for Hb measurement^31^. The eventual shift in measurement method will have important implications in the context of drawing conclusions across sources of monitoring data, national level statistics, and micro-level studies that utilize different and inconsistent methods of Hb measurement.

Our study limitations include the absence of a gold standard reference measure thus limiting our ability to assess and compare the accuracy of HemoCue and DBS. There are very few community-based studies validating capillary samples analyzed by the HemoCue 301 model. Existing research has found that compared the gold standard, HemoCue overestimates Hb levels by 0.16 g/dL among adults^36^ and by 0.87 g/dL among children^37^, resulting in lower anemia prevalence. The accuracy of Hb measurement using indirect CMG of capillary blood was assessed in only one field-based study among adult women which found that indirect CMG results in a 0.67 g/dL lower Hb level compared to direct CMG^13^, leading to a higher anemia prevalence. Based on the available data, we were unable to assess variability by surveyor, which may have contributed to variability in HemoCue and DBS measures. Additionally, we were unable to report reliability for either method in our study. Previous research has reported intra-sample variability higher for DBS (SD of 1.0 g/dL) than for direct CMG (SD of 0.4 g/dL)^13^. Evidence on reliability of capillary HemoCue has found that while differences between repeat samples are non-significant, coefficients of variation range from 3.9 to 7.0%^16,28^. Comparing the reliability of two field-friendly methods, DBS and HemoCue, would be a valuable future analysis that will contribute to the interpretation of changes over time within individuals as well as population level trends.

To the best of our knowledge, this is the first study to assess agreement between HemoCue and indirect CMG using DBS among children and furthermore, compare agreement with an adolescent and adult female population. While capillary HemoCue is a widely used field-friendly method, machines are relatively costly and validity and reliability remain questionable. DBS presents an alternative method to HemoCue for use in field settings, one which can allow for testing of multiple analytes with a single sample, for example measures of iron status and inflammation which would be immensely valuable in contexts such as India. However, further research is required to assess factors that influence DBS accuracy and reliability for Hb estimation, especially among children, an important target group for anemia prevention and control.

## Conclusions

Our study found moderate agreement between two methods that are feasible in field settings, HemoCue and indirect CMG using DBS. The bias we observed was small but operated differently for females 12–40 years and children 6–59 months, and is dependent on Hb magnitude, which should be further explored. In addition, the two methods resulted in differing anemia prevalence estimates, demonstrating that the choice of Hb assessment method has important implications for individual anemia diagnosis and population prevalence estimates. Accurate and field-friendly Hb assessment methods are necessary to meaningfully identify targets and track progress towards anemia reduction. Indirect DBS is a potential alternative to the widely used HemoCue device. However, additional studies with comparison to the reference method are needed. We recommend further research to understand utilization of DBS methods for Hb assessment, especially among young children.

## Supplementary Information

Supplementary Information 1.

Supplementary Information 2.

## Data Availability

The data set used in this article is available from the corresponding author and other Principal Investigators upon reasonable request.

## Abbreviations

BFA: Body mass index-for-age z-score
BMI: Body mass index
CCC: Concordance correlation coefficient
CI: Confidence interval
CMG: Cyanmethemoglobin
DBS: Dried blood spots
FPS: Fair price shop
g/dL: Grams per deciliter
HAZ: Height-for-age z-score
Hb: Hemoglobin
ICMR: Indian Council of Medical Research
IFMR: Institute of Financial Management and Research
JPAL: Abdul Latif Jameel Poverty Action Lab
LoA: Limits of agreement
NARI: National AIDS Research Institute
SC/ST: Scheduled caste/scheduled tribe
SD: Standard deviation
WHZ: Weight-for-height z-score
