# Supplementary material for: Agreement between dried blood spots and HemoCue in Tamil Nadu, India
Source: Sci Rep. 2021 Apr 29;11:9285. doi: 10.1038/s41598-021-88425-y (PMC8085154; doi:10.1038/s41598-021-88425-y)
Supplement: Supplementary file 1 — Supplementary Information 1. [file 41598_2021_88425_MOESM1_ESM.pdf]

Agreement between two field-friendly methods of hemoglobin assessment: dried blood spots and HemoCue among females 12 to 40 years of age and children 6 to 59 months in Tamil Nadu, India

Reshma P Roshania[1], Rukshan V Mehta[1], Ashwini Shete[2], Aprajit Mahajan[3], Grant Miller[4], Alessandro Tarozzi[5], Reynaldo Martorell[1,6]*

*Corresponding Author: rmart77@emory.edu. Reynaldo Martorell, PhD, Hubert Department of Global Health, Rollins School of Public Health, Emory University, 1518 Clifton Road, NE, CNR 5005, Mailstop #1518-002-7BB, Atlanta, GA 30322 USA.

Tamil Nadu Rice Fortification Study | Baseline Questionnaire -1

தமிழ்நாடுஊட்டச்சத்துநிறைந்தஅரிசிகுறித்தஆய்வு | முதல் நிலைகேள்விப்பட்டியல்

<u>Questionnaire 1</u>
<u>கேள்விப்பட்டியல் 1</u>

<u>Table of contents:</u>
<u>பொருள்உள்ளடக்கம்:</u>

Questionnaire 1
கேள்விப்பட்டியல் 1

0. **Identifying location and consent**

   இடத்தைகண்டறிந்துஒப்புதலைபெறுதல்

   Surveyors will enter tracking information before entering the household, reading consent, and beginning the survey.

   ஆய்வாளர்கள்வீட்டிற்குள்நுழைந்துஒப்புதலைபடித்துக்காட்டிஆய்வைதுவங்குவதற்குமுன்பாகவீட்டின்அமைவிடதகவலைஉள்ளீடுசெய்யவேண்டும்.

1. **Household socio-demographics**

   வீட்டின்சமூகசார்ந்ததகவல்

   Surveyors will collect information about the religion, education level, employment, and assets of the family.

   ஆய்வாளர்கள்குடும்பத்திற்குரியமதம், கல்வி, வேலைமற்றும்சொத்துகுறித்ததகவலைவீட்டாரிடம்கேட்டுபெறவேண்டும்.

2. **Household Expenditure**

   வீட்டுசெலவு

Surveyors will look at how much money families are spending on different components such as food, health care, clothing, etc.

ஆய்வாளர்கள்உணவு, உடல்ஆரோக்கியம், உடைபோன்றபலதரப்பட்டகாரியங்களுக்காகஇந்தகுடும்பங்கள்எவ்வளவுபணத்தைசெலவழிக்கிறார்கள்என்பதைகேட்டுதெரிந்துக் கொள்ளவேண்டும்.

3. **PDS consumers**

PDS நுகர்வோர்கள்

This section examines what quantity of each type of rice the family has been allotted, and tries to understand attitudes towards PDS.

இந்தபிரிவின்மூலம்ஒருகுடும்பத்திற்குஒவ்வொருஅரிசியிலும்எத்தனைகிலோஒதுக்கப்பட்டுள்ளதுஎன்பதைகுறித்துஆராயப்பட்டுஅக் குடும்பம் PDS - யைகுறித்துஎன்னநினைக்கிறதுஎன்பதைபுரிந்துக்கொள்ளமுயற்சி மேற்கொள்ளப்படும்.

4. **Non-PDS consumers**

PDS சாராதநுகர்வோர்கள்

Directed at Non-PDShouseholds to understand why they do not take rice from FPS

இந்தபிரிவு PDS சாராகுடும்பங்கள்எதனால் FPS கடைகளிலிருந்துஅரிசிவாங்குவதில்லைஎன்பதைகுறித்துஅறிந்துக் கொள்வதற்காகஒதுக்கப்பட்டுள்ளது.

5. **Supplementation and health of women and children**

கூடுதல்ஊட்டச்சத்தும்பெண்கள்மற்றும்குழந்தைகளின்ஆரோக்கியமும்

This section aims to understand whether women and children are taking any other supplementation already that may affect the study's results.

இந்தஆய்வின்முடிவுகளைபாதிக்கும்வகையில்பெண்களும்குழந்தைகளும்முன்னரேவேறுஏதேனும்கூடுதல்ஊட்டச்சத்துமருந்துகளையோஅல்லதுபொருட்களையோசாப்பிட்டுவருகிறார்களாஎன்பதைபுரிந்துக்கொள்ளும்நோக்கத்திற்காகஇந்தபிரிவுகொடுக்கப்பட்டிருக்கிறது.

6. **Health Outcomes**

உடல்ஆரோக்கியவிளைவுகள்

This section works towards understanding the health of the respondents to see whether any symptoms correlate with anemia, or the respondent has another illness.

இந்தஆய்வில்பங்குகொள்ளும்நபர்களுக்குஇரத்தசோகையுடன்தொடர்புடையஅறிகுறிகள், அல்லதுவேறுஏதேனும்உடல்பாதிப்புஇருக்கிறதாஎன்பதைஅறிந்துக்கொள்ளும்நோக்கத்துடன்இந்தபிரிவுகொடுக்கப்பட்டிருக்கிறது.

7. **Cognition**

அறிவாற்றல்

Aims to test children's developmental levels by using the Developmental Milestones Checklist and the Wechsler Preschool and Primary Scale of Intelligence. The section also has a couple of questions to understand the nature of the child's household and whether there is enough stimulus to help improve development.

வளர்ச்சிஅளவுக்கோல்சோதனைபட்டியல்மற்றும்வெக்ஸ்லர்மழலையர்பள்ளிக்கூடம்மற்றும்அறிவாற்றலுக்கானஆரம்பஅளவுக்கோள்ஆகியவற்றைபயன்படுத்திகுழந்தைகளின்வளர்ச்சிநிலைகளைபரிசோதித்துபார்க்கஇந்தபிரிவுநோக்கம்கொண்டுள்ளது. மேலும்,குழந்தையின்வீட்டுத்தன்மையைபுரிந்துக்கொண்டு, அந்தவீட்டில்குழந்தையின்வளர்ச்சிக்குஉதவக்கூடியபோதுமானதூண்டுதலைதரும்பொருட்கள்இருக்கிறதாஎன்பதையும்புரிந்துக்கொள்வதற்குஉதவியாகஇந்தபிரிவில்ஒருஜோடிகேள்விகள்கூடுதலாககொடுக்கப்பட்டுள்ளது.

Section 0: Identifying the location & Consent

இடத்தைகண்டறிந்துஒப்புதலைபெறுதல்

Instructions to surveyor:

ஆய்வாளருக்கானகுறிப்புகள்:

This section has three parts:

இந்தபிரிவில்மூன்றுபகுதிகள்இருக்கிறது:

a) Part A needs to be prefilled before entering the house
பேட்டிகாணப்படும்வீட்டிற்குள்நுழைவதற்குமுன்பாகபகுதி A முன்நிரப்பப்படவேண்டும்.

b) Provide an introduction before moving into consent. Please ensure that written consent is given before proceeding with the survey. Read out the consent portion and/or allow the respondent to read the same, if they wish to. This is Part C. The primary respondent should a knowledgeable person in the household, preferably a woman above the age of 18. If this woman is unavailable, up to three return visits to the household should be made. After three failures to interview this woman, we drop this household and select one from the back-up list.

ஒப்புதல்பிரிவுக்குள்செல்வதற்குமுன்பாகஇந்தபேட்டிகுறித்தஒருஅறிமுகத்தைஅவர்களுக்குதரவும்.

•ஆய்வைதுவங்குவதற்குமுன்பாகஎழுத்துப்பூர்வஒப்புதல்பெற்றுக்கொள்வதைஉறுதிசெய்துக்கொள்ளவும்.பங்குகொள்பவருக்குஒப்புதல்பிரிவைபடித்துக்காண்பிக்கவும்மற்றும்/அல்லதுஅவர்விரும்பினால், அவரேபடித்துப்பார்ப்பதற்குஅவரைஅனுமதிக்கவும்,

•இதுபகுதிC.பதில்அளிக்கும்பிரதானபங்காளர்வீட்டைப்பற்றியும்வீட்டிலுள்ளமற்றவர்களைபற்றியும்நல்லஅறிவுபெற்றவராகஇருக்கவேண்டும். மேலும்அவர்18 வயதுநிரம்பியஒருபெண்ணாகஇருப்பதுவிரும்பத்தகுந்தது.

•ஒருவேளைநீங்கள்வீட்டிற்குபோகும்போதுஅப்படிஒருபெண்வீட்டில்இல்லையெனில்,மீண்டும்அந்தவீட்டைநீங்கள்மூன்றுமுறைமற்றொருநேரத்தில்சென்றுபார்க்கவேண்டும்.

•ஒருவேளைஇப்படிமூன்றுமுறைஅந்தவீட்டைசந்தித்தபிறகும்அங்குள்ளஒருபெண்ணைஉங்களால்பேட்டிஎடுக்கமுடியவில்லைஎனில், அந்தவீட்டைகைவிட்டுவிட்டுஅடுத்தவீட்டிற்குநேராகபேட்டிகாண்பதற்குசெல்லவேண்டும்.

Part A: Identifying the location | பகுதி A: இடத்தைஅடையாளம்காணுதல்

Instructions to surveyor:
ஆய்வாளருக்கானகுறிப்பு:

This part of the section needs to be filled in by the surveyor prior to consent.

பங்காளருக்கானஏற்புடைமைபரிசோதனையைசெய்வதற்குமுன்பாகஇந்தபகுதியிலுள்ளகேள்விகளுக்குபதிலைஆய்வாளர்நிரப்பியாகவேண்டும்.

Q. No. கே. எண்	Question கேள்வி	Answer Options பதிலுக்கானதேர்வுகள்	Coding Instructions
0.00	Date of first visit முதல்சந்திப்புதேதி		Date should be programmed to show up automatically
0.01	Time of first visit முதல்சந்திப்புநேரம்		Time should be programmed to show up automatically
0.02	Surveyor ID ஆய்வாளர்அடையாளஎண்	_ _	We will provide a list of ID's with corresponding names<br>3 digit numeric entry only
0.03	Surveyor name ஆய்வாளர்பெயர்		We will provide a list of names with corresponding ID's so that once the ID is filled in, the name fills in. but make the name editable
0.04	Taluk name தாலுகாபெயர்	1. Chidambaram சிதம்பரம்<br>2. Bhuvanagiri புவனகிரி	
0.05	Area type பகுதியின்வகை	0. Rural கிராமப்புறம்<br>1. Urban நகர்ப்புறம்	

0.05A	FPS area code<br>ரேஷன்கடையின்கு றியீடு		Enter code. Numeric. Check in pre-filled data if this FPS area code exists in the Taluk given in 0.04. If it does not, show error message “This FPS code is wrong” and let the surveyor renter this field.
0.05B	FPS area name<br>ரேஷன் கடையின் பெயர்		To automatically appear after entering ID. List of FPS IDs and corresponding names to be shared with data team.
0.07A	Household ID<br>வீட்டு அடையாள எண்		6 digit numeric entry Check if this ID exists in the FPS code entered in 0.05A. If it does not, show error message “This HHID does not exist in the FPS code” and don’t let the questionnaire move forward.
0.07B	Ration card ID used to track this household<br>**குடும்பஉறுப்பின ருக்கு**சொந்தமான ரேஷன்அட்டைஅ டையாளஎண்.<br>ஆய்வாளருக்கான குறிப்பு: இந்தவீட்டைநீங்கள் கண்டுபிடிப்பதற்கு உதவியாகஉங்களி டம்கொடுக்கப்பட்ட ரேஷன்அட்டைவிவ ரங்களைஇங்குஉள் ளீடுசெய்யவும்.		Check if this ration card ID matches the Household ID entered in 0.07A. If it does not, show error message “Ration card ID does not match Household ID” and don’t let the questionnaire move forward.

0.07C	Is the name of the card owner correct?<br><br>ஸ்மார்ட் கார்டு வைத்திருப்பவரின் பெயர் சரியாக உள்ளதா?	0. No இல்லை<br>1. Yes ஆம்	Automatically display this name based on the ration card ID entered in 0.07B.<br>If '0' is selected then show the error message "Check the household ID and ration card details".<br>Don't let the questionnaire move forward after if 0 is selected.
0.07D	Which household ID is this household replacing?<br>எந்த குடும்பத்திற்கு பதிலாக இந்த குடும்ப அடையாள எண் மாற்றப்படுகிறது?	1. Enter ID<br>2. Main list completed மெயின் லிஸ்ட் முடிந்துவிட்டது	Only ask if the household ID entered in 0.07A is a replacement ID (refer to pre-fill)<br>If "Main list completed" is selected then directly go to 0.09. If "Enter ID" is selected then enter the ID 6 digit numeric entry.<br>Check if this household ID exists in the FPS code given in 0.05A. If it does not, show error message "please check ID"<br>Check if this hh ID is NOT a replacement HH ID, i.e.- replacement_hh column for this hh ID should be 0. If not show error message and don't let them move forward.
0.09	What is the status of this household?<br>இந்த வீட்டின் நிலவரம் என்ன?<br><u>Instruction to surveyor:</u><br>If door is locked, ask neighbors if it is permanently locked or if	1. Available பதில் அளிப்பவர் இருக்கிறார்<br>2. Temporary door lock தற்காலிகமாகவீடுபூட் டப்பட்டிருக்கிறது<br>3. Permanent door lockவீடுநிரந்தரமாகபூ ட்டப்பட்டிருக்கிறது	If 1 then move to the next section (0.09c)<br><br>If 3/6 in 0.09 then skip to 9.0A.<br><br>If 2/4/5 then go to 0.09B

	the person will be available in the next 3 days. If you are not sure if door is permanently locked, visit household 3 times (with appointment) before selecting option 3.<br>ஆய்வாளருக்களுக்கான குறிப்பு:<br>ஒருவேளைவீடுபூட்டப்பட்டிருந்தால், அருகில்வசிக்கும்யாரிடமாவதுசென்றுஇந்தவீடுஎப்போதுமேபூட்டப்பட்டுதான்இருக்கிறதாஅல்லதுஇந்தவாரத்தில்அவர்கள்மீண்டும்வீட்டிற்குவருவார்களாஎன்றுஅவர்களிடம்கேட்கவும்.<br>ஒருவேளைஅந்தவீடுநிரந்தரமாகபூட்டப்பட்டுதான்இருக்கிறதுஎன்பதைகுறித்துஉங்களுக்குநிச்சயமாகதெரியவில்லைஎனில், அந்தவீட்டைமேலும் 3 முறைசென்றுசந்திக்கவும் (சந்திப்புநேரம்குறித்துஉரியவீட்டாரிடம்அனுமதிபெறப்பட்டது) தேர்வு 3 -யைசெலக்ட்செய்வதற்குமுன்பாக.	4. Appointment given (not free now) நேரம்கொடுக்கப்பட்டுள்ளது (இப்போதுஅவர்வீட்டில்இல்லை/பேட்டிகொடுக்கநேரமில்லை)<br>5. Knowledgable person / adult not available வீட்டைக்குறித்துஅறிவுப்பெற்ற / பெரியவர்யாரும்இல்லை<br>6. Household has migrated இந்த குடும்பத்தினர் இடம் பெயர்ந்து விட்டனர்	
0.09B	Appointment date and time<br>பின்னர் சந்திக்கும் தேதி மற்றும் நேரம்	DD/MM<br><br>HH:MM	Select day and month. Should not be more than 5 days from start date.<br><br>HH:MM, select from drop-down of 30 minute intervals<br><br>Should not allow any date before the current date

			(but the current current date should be allowed)
Display message on screen if/after appointment entered:<br>சந்திப்பு நேரத்தை உள்ளிட்டவுடன் திரையில் காண்பிக்கப்பட வேண்டிய செய்தி:<br><br>**Press the back button on the bottom of the tablet, select 'save changes', and move to the next household. If the appointment is on the same day, keep a note of this and revisit the household on the same day.**<br>**டேப்ளட்டின் கீழ்ப்புறத்தில் காணப்படும் 'back' பொத்தானை அழுத்தவும், 'save changes' -யை தேர்வை செலக்டு செய்துவிட்டு அடுத்த வீட்டிற்கு நேராக கடந்து செல்லவும். ஒருவேளை ஒதுக்கப்படும் சந்திப்பு நேரம் அதே நாளில் இருக்குமெனில், அதை குறிப்பெடுத்து வைத்துக்கொண்டு அந்த வீட்டை அதே நாளில் திரும்ப வந்து சந்திக்கவும்.**			
0.09C	Is any eligible member as given in the tracking and targeting sheet available for the survey period?<br><br>கொடுக்கப்பட்ட ட்ராக்கிங் மற்றும் டார்கெட்டிங் சீட்டில் உள்ள தகுதி வாய்ந்த நபர் யாரேனும் ஆய்வு காலத்தில் வீட்டில் இருக்கிறரர்களா?	0 No<br>இல்லை<br>1 Yes<br>ஆம்	If 0, skip to 9.0A.

Part B: Introduction and Consent

Good morning/ afternoon/ evening. My name is [STATE YOUR NAME] and I work with the Abdul Latif Jameel Poverty Action Lab South Asia (J-PAL South Asia) at the Institute for Financial Management and Research based in Chennai. J-PAL is a non-governmental organization (NGO), which conducts research on social and health subjects.

காலை / பிற்பகல் / மாலை வணக்கம் . என்னுடைய பெயர் [உங்கள் பெயர் குறிப்பிடவும்] மற்றும் நான் சென்னை சார்ந்த நிதி மேலாண்மை மற்றும் ஆராய்ச்சி நிறுவனம், அப்துல் லத்தீஃப் ஜமீல் வறுமை ஒழிப்பு

நடவடிக்கை ஆய்வகம் (J-PAL தெற்காசியா) உடன் பணியாற்றுகிறேன். J-PAL என்பது அரசு சாரா அமைப்பு (NGO), இது சமூக மற்றும் சுகாதாரம்பற்றிய ஆராய்ச்சி நடத்துகிறது.

Things to keep in mind:

மனதில் கொள்ள வேண்டிய விஷயங்கள்:

1. Your name
   உங்கள் பெயர்
2. Organisation (J-PAL)
   நிறுவனம்(J-PAL)
3. Partner: Govt. of Tamil Nadu
   தமிழ்நாடு அரசுடன் இணைந்து
4. Why you are doing the survey (women and children in Cuddalore are highly anaemic and govt. wants to se what can be done to reduce it)
   எதற்காக இந்த ஆய்வு செய்கிறோம்(கடலூர் மாவட்ட பெண்கள் மற்றும் குழந்தைகளுக்கு ரத்த சோகை அதிகமாக உள்ளது மற்றும் அரசு அதை கட்டுப்படுத்த வேண்டி ஆய்வு செய்கிறோம்
5. Time of survey: 1 hr
   எடுத்து கொள்ளும் நேரம்(1 மணி நேரம்)
6. Data security: We will keep your information safe
   தகவல் பாதுகாப்பு : நீங்கள் கூறும் தகவல் பாதுகாப்பாக இருக்கும்
7. What will we do in the survey? Part A) Household; Part B) Health camp
   இந்த ஆய்வில் என்ன செய்வோம்?(பார்ட் A வீட்டில் ஆய்வு, பார்ட் B சுகாதார முகாம்
8. Get voluntary written consent
   தன்னார்வ எழுத்து பூர்வமான அனுமதி பெறுதல்

Part C: Identifying and verifying household information

பகுதி C: வீடுகுறித்ததகவலைஅடையாளம்கண்டுசரிபார்த்தல்

**Q. No.<br>கே. எண்**	**Question<br>கேள்வி**	**Answer Options<br>பதிலுக்கானதேர்வுகள்**	**Coding Instructions**
0.10	Did the respondent agree to participate in the study?	0. No, refused because respondent does not have the timeஇல்லை,பேட்டி அளிப்பவருக்கு	If response is ‘0’ ‘1, 2, 3, 4, go to 9.0A

	இந்தஆய்வில்பங்கு பெறபதில்அளிப்பவர் ஒப்புக்கொண்டாரா?	நேரமில்லை என்பதால் மறுத்துவிட்டார்<br>1. No, refused because not comfortable with other health measurementsஇல்லை,மற்ற ஆரோக்கிய அளவீடுகளில் வசதியாக உணரவில்லை என்பதால் மறுத்துவிட்டார்<br>2. No, refused because did not want to give blood இல்லை,இரத்தம் கொடுக்க விரும்பாததால் மறுத்துவிட்டார்<br>3. No, refused because lack of interestஇல்லை,பேட்டிக்கு ஆர்வம் இல்லாததால் மறுத்துவிட்டார்<br>4. No, refused because of other reasons (specify) இல்லை,மற்ற காரணங்களுக்காக மறுத்துவிட்டார் (குறிப்பிடவும்))<br>5. Yes ஆம்	
0.11	Name of respondent பதில்அளிப்பவரின் பெயர்		Display a list of all the adults in the household from prefill (i.e.- who are above 18 years)
0.12	What is your relationship with the Head of the household? இவ்வீட்டின்குடும்பத் தலைவருடன்உங்க ளுக்குஎன்னஉறவு?	1. Self குடும்பத்தலைவர்<br>2. Spouse of head of household குடும்பத்தலைவரின்ம னைவி/கணவன்<br>3. Married child	Select one option only

	Head of Household:<br>The head of the Household is someone who lives in the household and may be considered the head on the basis of age, sex, economic status etc. He/ she usually takes the final decision on all family activities and needs.<br>குடும்பத்தலைவர்:<br>குடும்பத்தலைவர்என்பவர்அந்தவீட்டில்வசிப்பவர்.<br>மேலும்அவரதுவயது, பாலினம், பொருளாதாரநிலை முதலியவற்றின்அடிப்படையில்குடும்பத்தின்தலைவர்எனஏற்றுக்கொள்ளப்பட்டவர்ஆவார்.<br>பொதுவாகஅனைத்துகுடும்பவிஷயங்களிலும்மற்றும்தேவைகளிலும்அவரேஇறுதிமுடிவைஎடுப்பார்.	திருமணஆனகுடும்பத்தலைவரின்மகன்/மகள்<br>4. Spouse of married child (daughter in law- son in law) திருமணமானமகன்/மகளின்மனைவி/கணவன் (மருமகள்/மருமகன்)<br>5. Unmarried child குடும்பத்தலைவரின்திருமணமாகாதமகன்/மகள்<br>6. Grandchild பேரக்குழந்தை<br>7. Mother/father அப்பா / அம்மா<br>8. Mother/father in law of head of household குடும்பத்தலைவரின்மாமனார் / மாமியார்<br>9. Brother/ sister/brother-in-law/sister-in-law குடும்பத்தலைவரின்சகோதரன் / சகோதரி / மைத்துனன் / கொழுந்தியாள்<br>10. Non-relatives எந்த சொந்தமும் இல்லை<br>11. Other (specify) மற்றவை (குறிப்பிடவும்)<br>-99 Don’t know தெரியாது<br>-98 Refused to Answer பதில்கூறமறுத்துவிட்டார்	
0.13	Street name<br>தெருப்பெயர்		
0.14	House Number<br>கதவுஎண்		Allow special characters

0.14A	Landmark<br>லேண்ட்மார்க்<br><br><u>Instruction to surveyor:</u><br>Write down any landmark that you can see that would make it possible for another person to visit the household in your absence.<br>பேட்டி காண்பவருக்கான குறிப்பு :<br>நீங்கள்ஆய்வுக்களத்தில்இல்லாதபோதுவேறு ஒருவர்நீங்கள்சந்தித்த வீட்டைசரியாகஅடையாளம்கண்டுக்கொள்ளவசதியாகஅந்தவீட்டிற்குஅருகில்உள்ளஉங்கள்கண்களால்பார்க்கக்கூடியஏதேனும்ஒருலேன்ட்மார்க்கைஇங்குகுறிப்பிடவும்.		Text entry
0.15	Cell Phone number<br>மொபைல்நம்பர்	--	Only allow 10 digit numbers
0.16D	Is the ration card a smart card?<br>இந்த ரேஷன் கார்டு ஸ்மார்ட் கார்டா?	0. No<br>இல்லை<br>1. Yes<br>ஆம்	
0.18	Do you consume the rice that you get from this card?<br>இந்தரேஷன்அட்டை மூலம்உங்களுக்குகிடைக்கும்அரிசியைநீங்கள் சாப்பிட உபயோகிக்கிறீர்களா ?Instruction to surveyor: This means	0 No<br>இல்லை<br>1 Yes<br>ஆம்<br>-99 Don't know<br>தெரியாது<br>-98 Refused to Answer<br>பதில்கூறமறுத்துவிட்டார்	

	take rice from the FPS and consume it themselves.<br>ஆய்வாளருக்கானகுறிப்பு:இதன் அர்த்தம்: ரேஷன் கடையிலிருந்து வாங்குற அரிசியை அவங்களே சாப்பிட பயன்படுத்துகிறார்களா? .		
0.19	Does your household consume ration rice through other sources?<br><br>இந்த கார்டுல மட்டும்தான் ரேஷன் அரிசி வாங்குறீங்களா இல்ல வேற வழியாகவும் வாங்குறீங்களா. அதாவது,மற்றொரு ரேஷன் கார்டு பயன்படுத்தியோஉங்களுக்கு தெரிஞ்சவங்க கிட்ட இருந்து அவங்க கார்டுலயோ, அல்லது தெரிஞ்சவங்க கிட்ட விலை கொடுத்தோ ரேஷன் அரிசி வாங்கி பயன்படுத்துறீங்களா??<br>Note to surveyor: Prompt by asking if they have received rice from other	1. Yes, rice bought/taken from friend/neighbour/family/employer in the same village or using a card from a friend/neighbour/family/employer in the same village<br>ஆம், **அதேகிராமத்தை** சேர்ந்தநண்பர்கள்/அக்கம்பக்கத்தினர்/சொந்தக்கார்கள்/தொழிலாளர்கள் ரேஷன் கார்டு பயன்படுத்தி அல்லது அவர்களிடமிருந்து ரேஷன் அரிசியை வாங்குவோம்<br>2. Yes, rice bought/taken from friend/neighbour/family/employer from a different village or using a card from a friend/neighbour/family/employer in a different villageஆம், **வேறு கிராமத்தை**சேர்ந்தநண்பர்கள்/அக்கம்பக்கத்தினர்/சொந்தக்கார்க்ள்/ தொழிலாளர்கள் ரேஷன் கார்டு பயன்படுத்தி அல்லது அவர்களிடமிருந்து	Allow multiple choices to be selected here.

	family members' cards, neighbours, friends, etc.<br><br>ஆய்வாளருக்கானகுறிப்பு: இதர குடும்ப உறுப்பினர்மற்றும்உறவினர்,நண்பர்கள்அக்கம்பக்த்தினர்போன்றபிறருக்குசொந்தமானரேஷன்அட்டையின்மூலம்வாங்கப்படும்அரிசியைவாங்கி பயன்படுத்துவார்களாஎன்றுகேட்கவும்.	ரேஷன் அரிசியை வாங்குவோம்<br>3. Yes, using another card that belongs to a member of the householdஆம், **இதேவீட்டைசேர்ந்தமற்றொரு**குடும்பஉறுப்பினரின்குடும்பஅட்டையைபயன்படுத்திஅரிசியைவாங்குவோம்<br>4. No, we don't consume PDS rice from other sourcesஇல்லை, நாங்கள் மற்ற வழிவகைகளில் PDS அரிசி வாங்கி பயன் படுத்துவதில்லை-99 Don't know<br>தெரியாது<br>-98 Refused to Answer<br>பதில்கூறமறுத்துவிட்டார்	
0.20A	Enter details of the ration shop from which you get rice for this household, using another ration card that you own<br>உங்க வீட்டுல இருக்க இன்னொருத்தரோட ரேஷன் கார்டுலயும் அரிசி வாங்குவதா சொன்னீங்க இல்லயா. அந்த ரேஷன் ரேஷன் கர்ர்டு எந்த ரேஷன் கடையைச் சேர்ந்தது?	Shop ID:<br>கடையின்அடையாளஎண்:<br>Don't know<br>தெரியாது<br>Shop Name:<br>கடையின்பெயர்:<br>Don't know<br>தெரியாது	Only ask this question if 3 selected in 0.19<br><br>Shop ID should be alphanumeric<br><br>Shop name should only text entry

0.20B	Enter details of the ration shop from which your friend/neighbour/family gets rice for your household உங்களுக்குத் தெரிஞ்சவங்களோட கார்டுலயும் அரிசி வாங்குறதா சொன்னீங்க இல்லயா. அந்த கார்டு எந்த ரேஷன் கடயைச் சேர்ந்தது?	Shop ID: கடையின்அடையாளஎண்: Don't know தெரியாது Shop Name: கடையின்பெயர்: Don't know தெரியாது	Ask if 1/2was selected in 0.19 Shop ID should be alphanumeric Shop name should only text entry

0.22 I am going to read out some names. Please tell me if the person is living in this household or not.
நான் சில பெயர்களை படிக்கப் போகிறேன். அவர் இந்த வீட்டில் வசிக்கிறாரா இல்லையா என்று என்னிடம் சொல்லுங்கள்.

0.22	Is [name] [member_ID] still living in this household? [name] [member ID] அவர்கள் தற்சமயம் இந்த வீட்டில் வசிக்கிறாரா?	0.Not living in the hh anymore இவர் முன்னே வசித்து வந்தார். ஆனால் இனிமேல் இந்த வீட்டில் வசிப்பதில்லை 1. Still living in the hh இன்னும் இந்த வீட்டில்தான் வசிக்கிறார்	Programmer instruction: Please display all the names from the pre-filled household roster.
0.22A	Why does [name] not live in the household anymore? இவர் இந்த வீட்டில் இப்போது	1. Died இறந்துவிட்டார் 2. Migrated for work	Ask this question in a roster form for each member where 0.22 is 0.

	வசிக்காததற்கு என்ன காரணம்?	வேலைக்காக இடம் பெயர்ந்துவிட்டார்<br>3. Migrated for education படிப்புக்காக இடம் பெயர்ந்துவிட்டார்<br>4. Others: Please specify மற்றவை : குறிப்பிடவும்<br>5. Migrated because family/family members migrated குடும்பம் குடிபெயர்ந்தது / குடும்ப உறுப்பினர்கள் குடிபெயர்ந்தார்கள்	
0.22B Check if there is at least 1 eligible woman/eligible child still living in the hh (i.e.- in the pre-fill data, mem_elig_wom or mem_elig_child is 1 AND for that person, 0.22=1). If this condition is met then move to 0.23. Else, display "No eligible woman or child in the household இந்த குடும்பத்தில் தகுதி வாய்ந்த பெண்மணி அல்லது குழந்தை இல்லை" and then go to 9.0A.			
Instruction to data team: Ask the next two questions (0.23 and 0.24) in a roster format for all the eligible children aged 6 months to 59 months, and women aged 12-40, who are still living at home (i.e.- 0.22=1). Skip 0.23 and 0.24 if there are no women in the household roster that are older than 18 years AND 0.22=1). The household roster information is available in the pre-fill for each household ID			
0.23	Does [name]'s mother live in this household?	0. No இல்லை<br>1. Yes	If 0,-99,-98, skip 0.24

	(பெயர்)___ அவர்களின் தாயார் இந்த வீட்டில் வசிக்கிறாரா?	ஆம்<br>-99 Don't know தெரியாது<br>-98 Refused to Answer பதில்கூறமறு த்துவிட்டார்	
0.24	Who is the mother of [name]?<br>(பெயர்)___ அவர்களின் தாயார்பெயர் என்ன?		Provide a list of all women ages 18 and above from the household roster. The list should not include the [name] in the question i.e show the list of all women aged 18 and above from the roster, except the name used in the question itself.
0.24A	Who is the head of the household?<br>இந்த வீட்டின் குடும்ப தலைவர் யார்?		Show a list of all adults in the house older than 18 years as answer options along with -99 Don't know and -98 Refused to Answer.
Instruction to Data team: Repeat 0.25-0.29 as a roster for every member who is still living in the hh (i.e.- 0.22=1)			
0.25	Date of Birth பிறந்ததேதி	1. Enter Date of Birth பிறந்ததேதி<br>-99 Don't know தெரியாது<br>-98 Refused to Answer பதில்கூறமறுத்து விட்டார்	Go to 0.26 if '1' selected<br>Got to 0.27 if -99/-98 selected

0.26	Date of Birth<br>பிறந்ததேதி	Dd/mm/yyyy	Don't allow dates after date of the survey<br><br>Go to Section 1/next person in the roster after this
0.27	Age:<br><br>வயது:	1. Age in months, for children 5 years old and below<br><br>வயது (மாதக்கணக்கில்), 5 வயது மற்றும் அதற்கு கீழ் உள்ள குழந்தைகளுக்காக<br><br>2. Age in years, for everyone above 5 years<br><br>வயது (வருடக்கணக்கில்), 5 வயதுக்கு மேற்பட்ட அனைவருக்கும்<br><br>-99 Don't know<br>தெரியாது<br><br>-98 Refused to Answer<br>பதில்கூறமறுத்து விட்டார்	Go to Section 1/next person in the roster if -99/-98<br><br>Go to 0.28 if '1'<br>Go to 0.29 if '2'
0.28	Age in months<br><br>வயது (மாதக்கணக்கில்)		Numeric entry between 0-60, no decimals allowed

			Go to Section 1/next person in the roster after this
0.29	Age in years<br><br>வயது (வருடக்கணக்கில்)		Numeric entry between 5-120, no decimals allowed<br><br>Go to Section 1/next person in the roster after this
0.30	Instruction to data team: Check if there is<br>1. a woman (check if mem_elig_wom=1) AND she is between the age of 12 to 40 years (check 0.25-0.29)<br>OR<br>2. a child (check if mem_elig_child=1) AND the child is between 6-59 months (check 0.25-0.29)<br>If neither of the above conditions are met, please display the following:<br>"No eligible woman or child in the household இந்த குடும்பத்தில் தகுதி வாய்ந்த பெண்மணி அல்லது குழந்தை இல்லை"<br>And then skip directly to 9.0A.		

Section 1: Household Socio-Demographics | பிரிவு 1: வீட்டின்சமூகவிவரங்கள்

Instruction to surveyor: This section is divided into two parts. Both parts need to be addressed to any knowledgeable member of the household/wife of head of household.

ஆய்வாளருக்கானகுறிப்புகள்: இந்தபிரிவுஇரண்டுபகுதிகளாகபிரிக்கப்பட்டுள்ளது. இந்தஇரண்டுபகுதிகளிலுமேஉள்ளகேள்விகளைகுடும்பத்தை பற்றிய விவரம் தெரிந்த வீட்டுஉறுப்பினரிடம்/குடும்பத்தலைவரின்மனைவியிடம்கேட்கவேண்டும்.

Part 1: Education and Employment | பகுதி 1: கல்வி மற்றும் வேலை

Data team to provide a household roster of names as given in pre-fill. These questions will be asked for ALL household members if 0.22=1 for that member

.

Q. No.<br>கே. எண்	Question<br>கேள்வி	Answer Options<br>பதிலுக்கானதேர்வுகள்	Coding Instructions
	Name of member உறுப்பினரின்பெயர்<br>ID of member உறுப்பினரின்அடையாளஎண்<br><br>Instruction to data team: Display the name and ID of each member in the roster before asking 1.00		
1.00	What is [name]'s education level?<br><பெயர்> -அவர்கள்கல்விதகுதிஎன்ன?	0. Not literate<br>கல்வியறிவுஇல்லாதவர்<br>1. Literate without formal schooling: EGS Education (Guarantee Scheme), NFTC (no-formal education courses), AEC (Adult education Center Bachelors<br>முறையாகபள்ளியில்படிக்காமல்கல்வியைகற்றுள்ளார்: EGS கல்வி (உத்தரவாததிட்டம்), NFTC (முறையானகல்விபயிற்சிகள்அல்ல), AEC (முதியோர்கல்விமையம்) இளங்கலை<br>2. Literate (Below Primary)<br>படித்தவர் (ஆரம்பகல்விக்கும்குறைவாக)<br>3. Literate (Middle)<br>படித்தவர் (நடுநிலைகல்விவரை)<br>4. Literate (Secondary)<br>படித்தவர் (உயர்நிலைகல்விவரை)<br>5. Literate (Higher secondary)<br>படித்தவர் (மேல்நிலைகல்விவரை)<br>6. Literate (Diploma/ certificate course)<br>படித்தவர் (டிப்ளோமா/சான்றிதழ்படிப்பு)<br>7. Literature (graduate)<br>படித்தவர் (பட்டம்பெற்றவர்)<br>8. Literate (post graduate and above)<br>படித்தவர் (முதுகலைபட்டம்மற்றும்அதற்குமேல்படித்தவர்)<br>-99 Don't know	

		தெரியாது<br>-98 Refused to Answer<br>பதில்கூறமறுத்துவிட்டார்	
1.01	Does [name of child] go to a palvadi or anganwadi center?<br><குழந்தையின்பெயர்> பால்வாடிக்குஅல்லதுஅங்கன்வாடிமையத்திற்கு போகிறாரா?	0. No<br>இல்லை<br>1. Yes<br>ஆம்	*Only ask for children below 5 years*
Instruction to data team: Only ask the following questions (1.02-1.06) for everyone in the roster who is between 12-75 years old and if the response to 0.22==1 for that member			
1.02	What was the main activity of [name] during the last year?<br>கடந்த ஒரு வருடத்தில்<பெயர்>அவர்கள் முக்கியமாகஎந்தவேலையைசெய்தார்?	1. self-employed in agriculture<br>விவசாயத்தில்சுயவேலைசெய்தார்<br>2. self-employed in non-agriculture<br>விவசாயம்சாராவேலையில்சுயவேலைசெய்தார்<br>3. regular wage/salary earning<br>மாதசம்பளத்திற்குவேலைசெய்தார்<br>4. casual labour in agriculture<br>விவசாயதற்செயல்கூலியாளாகவேலைசெய்தார்<br>5. casual labour in non-agriculture<br>விவசாயம்சாராவேலையில்தற்செயல்கூலியாளாகவேலைசெய்தார்<br>6. attending school/college/ etc<br>பள்ளி/கல்லூரி / இதர.. செல்கிறார் (கல்வி பயின்று வருகிறார்)<br>7. domestic duties (House wife)<br>குடும்ப பணிவீட்டுவேலைகளைசெய்தார்<br>8. Unemployed and looking for work<br>வேலையில்லை, வேலை தேடிக்கொண்டிருக்கிறார்?	

		9. Unemployed and not looking for workவேலையில்லை மற்றும் வேலை தேடவில்லை<br>10. Retired/Aged ஒய்வு பெற்றவர்/ வயது முதிர்ந்தவர்<br>11. Pensioner ஒய்வு ஊதியம் பெறுபவர்<br>97 Other: Please specify மற்றவை: குறிப்பிடவும்<br>-99 Don’t know தெரியாது<br>-98 Refused to Answer<br>பதில்கூறமறுத்துவிட்டார்	
1.03	During the last week, how many days did [name] work?<br>கடந்த வாரத்தில், <பெயர்>அவர்கள் எத்தனைநாட்கள்வேலை செய்தார்	-- days<br>நாட்கள்<br>-99 Don’t know<br>தெரியாது<br>-98 Refused to Answer<br>பதில்கூறமறுத்துவி ட்டார்	Only ask if 1.02 is 1/2/3/4/5<br>Numeric entry for days restricted between 0-7
1.04	How many hours per day did [name] work for, on average, during the last week?<br>கடந்தவாரத்தில், சராசரியாகஒருநாளைக்கு எவ்வளவுநேரம்<பெயர்>அ வர்கள் வேலைசெய்தார்?	-- hours<br>மணிநேரம்<br>-99 Don’t know<br>தெரியாது<br>-98 Refused to Answer<br>பதில்கூறமறுத்துவி ட்டார்	Only ask if 1.02 is 1/2/3/4/5<br>Numeric entry for hours restricted between 0-24
1.05	How much is [name] paid for this job?<br>இந்த வேலையில் (பெயர்) அவர்கள் எவ்வளவு ஊதியம்/ ஒய்வு ஊதியம் பெற்றார்?	a) Frequency<br>எவ்வளவு அடிக்கடி<br>1. daily<br>தினசரி<br>2. weekly	Only ask if 1.02 is 3/4/5/11<br>Show this whole question on the screen at once.

	a) Frequency<br>எவ்வளவு அடிக்கடி<br>b) Amount<br>தொகை<br><br><u>Instruction to surveyor:</u> Ask the question as is phrased and let the respondent report what frequency they prefer. What was the mode of payment received?<br>கேள்வியை அதே வார்த்தைகள் கொண்டு கேட்கவும்.பங்கு கொள்பவர் பதில் அளிக்கட்டும்.எந்த இடைவெளியில் அல்லது எந்த வகையில் அவர்களுக்கு சம்பளம் வழங்கப்பட்டது.	வாராந்திரம்<br>3. monthly<br>மாதாந்திரம்<br>4. Quarterly (every 3 months) காலாண்டு (ஒவ்வொரு 3 மாதத்திற்கு ஒருமுறை)<br>5. Half yearly (every 6 months)<br>அரையாண்டு (ஒவ்வொரு 6 மாதத்திற்கு ஒருமுறை)<br>-99 Don't knowதெரியாது<br>-98 Refused to Answer<br>பதில்கூறமறுத்து விட்டார்<br>b) Amount<br>தொகை<br>Rs.__________<br><br>-99 Don't know<br>தெரியாது<br><br>-98 Refused to Answer<br>பதில்கூறமறுத்து விட்டார்	a) should be a numeric entry<br><br>b) should show the answer options<br><br>Should allow all positive values, including 0.
1.06	How much revenue/money did [name] make in the last 6 months?<br>[பெயர்] அவர்கள் கடந்த 6 மாதத்தில் எவ்வளவு பணம்(வருமானம்) சம்பாதித்தார்?	Rs. ___________<br><br>₹ _______<br>-99 Don't know<br>தெரியாது<br>-98 Refused to Answer<br>பதில்கூறமறுத்துவிட்டார்	Only ask if 1.02 is 1/2<br><br>Numeric entry. Should allow all positive values, including 0.

Part 2: Socio-demographics and assets | பகுதி 2: சமூகசார்ந்தவிவரங்களும்சொத்துக்களும்

Instructions to surveyor:

ஆய்வாளருக்கானகுறிப்பு:

Read out loud:

பின்வரும்வாக்கியத்தைசத்தமாகபடித்துக்காண்பிக்கவும்:

"In this section we shall ask you a few general questions about your household. If you are not comfortable answering these questions you may refuse to answer that particular question, in which case we shall skip that question and move ahead"

"இந்தபிரிவில்உங்கள்வீட்டைபற்றியஒருசிலபொதுவானகேள்விகளைநாங்கள்உங்களிடம்கேட்போம். அப்படிநாங்கள்கேட்கும்ஏதேனும்கேள்விகளுக்குபதில்சொல்லஉங்களுக்குசங்கடமாகஇருந்தால், அதைநீங்கள்எங்களிடம்சொல்லலாம். அப்போதுநாங்கள்அந்தகேள்வியைதவிர்த்துவிட்டுஅடுத்துகேள்விக்குசென்றுவிடுவோம். இப்படிநீங்கள்எந்தகேள்வியைவேண்டுமானாலும்தவிர்க்கும்படிஎங்களைகேட்டுக்கொள்ளலாம்."

**Q. No<br>கே. எண்**	**Question<br>கேள்வி**	**Answer Options<br>பதிலுக்கானதேர்வுகள்**	**Coding Instructions**
Instructions to surveyor: Before asking questions relating to income, religion and caste, tell the respondent that we are required to collect this information as part of our survey and will now be asking questions about their income, religion and caste. Please also inform the respondent that their answers will be kept completely confidential and no harm will ensue as a result of their participation. You may choose not to answer the following questions if you so wish.<br>**ஆய்வாளருக்கானகுறிப்புகள்:**<br>• வருமானம், மதம்மற்றும்ஜாதிப்பற்றியகேள்விகளைகேட்பதற்குமுன்பாக, பதில்அளிப்பவரிடம்இந்தஆய்வின்ஒருபகுதியாகநாங்கள்உங்களுடையமதம், வருமானம்மற்றும்ஜாதிப்பற்றியகேள்விகளைகேட்கஇருக்கிறோம்என்பதைதெளிவாகஎடுத்துசொல்லவும்.<br>• மேலும்,இந்தபகுதியில்அவர்கள்தெரிவிக்கும்பதில்கள்முற்றிலும்இரகசியாகபாதுகாக்கப்படும்என்றும்மற்றும்இந்தஆய்வில்பங்குகொள்வதன்விளைவாகபதில்அளிப்பவருக்கு**எவ்விதபாதிப்பும்ஏற்படாது**என்றும்அவர்களுக்குதெரிவிக்கவும்.			

• அப்படிஅவர்கள்விருப்பப்பட்டால்இந்தகேள்விகளுக்கு**பதில்சொல்லாமலும்கூடஇருக்கலாம்**என்றுஅவர்களுக்குதெரிவிக்கவும்.		
1.07	What is the religion followed by members of this household?<br>இந்தவீட்டைசேர்ந்தவர்கள்எந்தமதத்தைபின்பற்றுகிறார்கள்?	1. Hindu<br>இந்துமதம்<br>2. Muslim<br>முஸ்லிம்மதம்<br>3. Christian<br>கிறிஸ்தவமதம்<br>97 Other: Please specify<br>மற்றவை: குறிப்பிடவும்<br>-99 Don't know<br>தெரியாது<br>-98 Refused to Answer<br>பதில்கூறமறுத்துவிட்டார்
1.08	What caste do members of this household belong to?<br>இந்தவீட்டின்உறுப்பினர்கள்எந்தஜாதியை(சமூகப்பிரிவு)சேர்ந்தவர்கள்?	1. General<br>பொதுப்பிரிவினர்<br>2. Scheduled Caste/ Scheduled Tribe (SC/ST)<br>பழங்குடியினர்/மலைவாழ்மக்கள் (SC/ST)<br>3. Backward Classes (BC/OBC/MBC)<br>பிற்படுத்தப்பட்டபிரிவினர் (BC/OBC/MBC)<br>-99 Don't know<br>தெரியாது<br>-98 Refused to Answer<br>பதில்கூறமறுத்துவிட்டார்<br>97 Other: Please specify<br>மற்றவை: குறிப்பிடவும்
1.09<br>Surveyor observation qn<br>Is this a kutcha, semi-pucca or pucca house?<br><br>இந்த வீடு கச்சாவீடா, அரை பக்காவீடாஅல்லது பக்காவீடா?<br>Instructions to surveyors: This question should not be asked out loud. Please observe and answer yourself.		

ஆய்வாளருக்கானகுறிப்புகள்:
இந்தகேள்வியைபதில்அளிப்பவரிடம்கேட்கக்கூடாது.
அதற்குபதிலாகநீங்களேஉங்கள்கண்ணால்பார்த்துநிரப்பவேண்டும்.
A kutcha house is any establishment any part of which is built of low quality or naturally occurring material like tarpaulin, aluminum sheets, leaves, mud.

ஒருவீடுஅல்லதுவீட்டின்எந்தவொருபகுதியாவதுதரம்குறைந்தஅல்லது தார்ப்பாலின், அலுமினியதகடுகள், ஓலைகள், கலிமன்போன்றஇயற்கையாககிடைக்கும்பொருட்களைகொண்டுகட்டப்பட்டால்அதுகச்சாவீடுஎனப்படும்.
A pucca house is any establishment which has walls and roof made of: burnt bricks, stones (packed with lime or cement), cement concrete, timber, ekra etc.

ஒருவீட்டின்சுவரும்மேற்கூரையும்செங்கல், கல் (சுண்ணாம்புஅல்லதுசிமெண்டுகொண்டுகட்டப்பட்டது), சிமெண்டுகாண்கிரீட்டு, மரம், எக்ராமுதலியபொருட்கள்கொண்டுகட்டப்பட்டால்அதுபக்காவீடுஎனப்படும்.
A semi-pucca house has fixed walls made up of pucca material but the roof or other parts are made up of the material not used for pucca house such as straw e.g. thatched roof
ஒருவீட்டின்சுவர்பக்காபொருட்களாளும்மற்றும்அதன்கூரைமற்றும்இதரபகுதிகள்பக்காஅல்லாதபொருட்களால்அதாவதுவைக்கோலால்கட்டப்படும்கூரையால்கட்டப்பட்டால்அதுஅரைபக்காவீடுஎனப்படும்

Answer options:
1. Kutcha house
   கச்சாவீடு
2. Pucca house
   பக்காவீடு
3. Semi-pucca house
   அரை - பக்காவீடு

-99 Don't know
தெரியாது
-98 Refused to Answer
பதில்கூறமறுத்துவிட்டார்
97 Other: Specify
மற்றவை: குறிப்பிடவும்

1.10A	Do members of this householduse a mosquito bed net for sleeping?<br>இந்தவீட்டில்இருப்பவர்கள்தூங்குவதற்குகொசுவலையையபயன்படுத்துவார்களா?	0. No<br>இல்லை<br>1. Yes<br>ஆம்<br>-99 Don't know<br>தெரியாது<br>-98 Refused to Answer<br>பதில்கூறமறுத்துவிட்டார்	Only ask 1.10B if 1 and there is a child less than 18 years in the household.<br>If 0/-99/-98 is selected then skip to 1.11
1.10B	Does [name] use a mosquito bed net for sleeping?<br><பெயர்>தூங்குவதற்கு கொசுவலையையபயன்படுத்துகிறாரா?	1. No<br>இல்லை<br>2. Yes<br>ஆம்<br>-99 Don't know<br>தெரியாது<br>-98 Refused to Answer<br>பதில்கூறமறுத்துவிட்டார்	If there is an eligible child in the HH- ask this question for each child
1.11A	Has your house been sprayed with mosquito spray during the last six months?<br>Note to surveyor:<br>Done by the government (spraying/fumigating)<br>போனஆறுமாதங்களில் உங்கள்வீட்டில்கொசுமருந்துஅடித்திருக்கிறார்களா?<br>ஆய்வாளருக்கானகுறிப்பு:<br>அரசுதரப்பிலிருந்துமருந்துஅடிக்கப்பட்டது (தெளிப்புமருந்து/கொசுஒழிப்புபுகை)	0. No<br>இல்லை<br>1. Yes<br>ஆம்<br>-99 Don't know<br>தெரியாது<br>-98 Refused to Answer<br>பதில்கூறமறுத்துவிட்டார்	
1.11B	What type of toilet facilities does your household have?<br>உங்கள்வீட்டில்எப்படிப்பட்டகழிவறைஇருக்கிறது?	1. Private toilet (inside the house/compound)<br>தனிப்பட்டகழிவறை (வீட்டிற்குள்/காம்பவுண்டிற்குள்)<br>2. Shared/communal toilet (inside/outside the compound)	

		பொது/பகிர்ந்துக்கொள்ளப்பட்டகழிவறை (காம்பவுண்டிற்குள்/வெளியே)<br>3. No toilet (open usage) கழிவறையேஇல்லை (திறந்தவெளிபயன்பாடு)<br><br>-99 Don't know தெரியாது<br>-98 Refused to Answer பதில்கூறமறுத்துவிட்டார்

1.12	Where do you get your drinking water from? எங்கிருந்துகுடிதண்ணீரைகொண்டுவருகிறீர்கள்?<br><br>1. Piped into dwelling வீட்டிற்குள்ளேயேகுழாய்போடப்பட்டிருக்கிறது<br>2. Piped to yard/plot மனைக்குள்குழாய்போடப்பட்டுள்ளது<br>3. Public tap/standpipe பொதுகுழாய்/அடிகுழாய்<br>4. Tube well or borehole ஆழ்குழாய்கிணறு<br>5. Protected well பாதுகாக்கப்பட்டகிணறு<br>6. Unprotected well பாதுகாக்கப்படாதகிணறு<br>7. Protected spring பாதுகாக்கப்பட்டநீருற்று<br>8. Unprotected spring பாதுகாக்கப்படாதநீருற்று<br>9. River/dam/lake/ponds/stream/canal/irrigation channel நதி/அணை/குளம்/ஏரி/நீரோடை/வாய்க்கால்/நீர்பாசனகால்வாய்<br>10. Rainwater மழைநீர்<br>11. Tanker truck தண்ணீர்லாரி<br>12. Cart with small tank மாட்டுவண்டிடேங்கு	Allow multiple answers to be selected

	13. Bottled/packet water<br>கேன்தண்ணீர்/பாக்கெட்செய்யப்பட்டதண்ணீர்<br>14. Community RO Plant<br>பொது RO குழாய்<br>15. Water purifier system<br>நீர்சுத்திகரிப்புஇயந்திரம்<br><br>-99 Don't know<br>தெரியாது<br>-98 Refused to Answer<br>பதில்கூறமறுத்துவிட்டார்<br>97 Other: Specify<br>மற்றவை: குறிப்பிடவும்		
1.13	Do you do anything to water to make it safe to drink (boil, add bleach/chlorine, filter etc.)<br>குடிநீரை அப்படியே குடிப்பீர்களா இல்லை குடிப்பதற்கு முன் எதாவது செய்வீங்களா?<br>prompt:<br>கொதிக்கவைத்தல், பிளீச்சிங்பவுடர்/குளோரின்சேர்த்தல், வடிகட்டுதல் அந்த மாதிரி)	0. No<br>இல்லை<br>1. Yes<br>ஆம்<br>2. Sometimes<br>சிலநேரங்களில்<br><br>-99 Don't know<br>தெரியாது<br>-98 Refused to Answer<br>பதில்கூறமறுத்துவிட்டார்	
1.14	What kind of access to electricity do you have?<br>உங்க வீட்டில் மின்சாரம் இருக்கா?எவ்வகைமின்சாரம்உங்களுக்குகிடைக்கிறது?	0. None<br>எதுவுமில்லை<br>1. Regular supply: daily supply, minimal power cuts<br>வழக்கமானசப்ளை: தினசரிசப்ளை, குறைந்தபட்சமின்வெட்டுகள்<br>2. Irregular supply: frequent unplanned power cuts, and<br>வழக்கத்திற்குமாறானசப்ளை: அவ்வப்போதுஏற்படும்திட்டமிடப்படாதமின்வெட்டுகள்<br>-99 Don't know	

		தெரியாது<br>-98 Refused to AnswerR1A<br>பதில்கூறமறுத்துவிட்டார்<br>97 Other: Specify<br>மற்றவை: குறிப்பிடவும்	
1.15	Does this household have a television?<br>உங்களிடம்டீ.வி. இருக்கிறதா?	0. No<br>இல்லை<br>1.Yes<br>ஆம்<br>-99 Don't know<br>தெரியாது<br>-98 Refused to Answer<br>பதில்கூறமறுத்து விட்டார்	Display 1.15 to 1.20 all on the same screen
1.16	Does this household have a refrigerator?<br>உங்களிடம்பிரிட்ஜ்இருக் கிறதா?	0. No<br>இல்லை<br>1. Yes<br>ஆம்<br>-99 Don't know<br>தெரியாது<br>-98 Refused to Answer<br>பதில்கூறமறுத்துவிட்டார்	
1.17	Does this household have a bicycle?<br>உங்களிடம்சைக்கிள்இரு க்கிறதா?	0. No<br>இல்லை<br>1. Yes<br>ஆம்<br>-99 Don't know<br>தெரியாது<br>-98 Refused to Answer<br>பதில்கூறமறுத்துவிட்டார்	
1.18	Does this household have a motorcycle?<br>உங்களிடம்மோட்டர்சை க்கிள்இருக்கிறதா?	0. No<br>இல்லை<br>1. Yes<br>ஆம்<br>-99 Don't know<br>தெரியாது<br>-98 Refused to Answer<br>பதில்கூறமறுத்துவிட்டார்	

1.19	Does this household have an animal drawn cart?<br>உங்களிடம்மாட்டுவண்டி/குதிரைவண்டிஇருக்கிறதா?	0. No<br>இல்லை<br>1. Yes<br>ஆம்<br>-99 Don't know<br>தெரியாது<br>-98 Refused to Answer<br>பதில்கூறமறுத்துவிட்டார்	
1.20	Does this household have a car/truck?<br>உங்களிடம்கார்/டிரக்இருக்கிறதா?	0. No<br>இல்லை<br>1. Yes<br>ஆம்<br>-99 Don't know<br>தெரியாது<br>-98 Refused to Answer<br>பதில்கூறமறுத்துவிட்டார்	
1.21	Does this household own land usable for agriculture?<br>உங்களிடம்விவசாயநிலம்இருக்கிறதா?	0. No<br>இல்லை<br>1. Yes<br>ஆம்<br>-99 Don't know<br>தெரியாது<br>-98 Refused to Answer<br>பதில்கூறமறுத்துவிட்டார்	Skip 1.22 (and got to 1.23) if 0/-99/-98
1.22	How many acres of agricultural land does this household own?<br>உங்களிடம்எவ்வளவுஏக்கர்/சென்டுவிவசாயநிலம்இருக்கிறது?	___ Acres (use 1 decimal)<br>_____ ஏக்கர் (ஒருதசமஎண்வரைபயன்படுத்தலாம்)<br>-99 Don't know<br>தெரியாது<br>-98 Refused to Answer<br>பதில்கூறமறுத்துவிட்டார்<br>97 Other: Specify<br>மற்றவை: குறிப்பிடவும்	
1.23	Does this household own cattle?	0. None<br>எதுவுமில்லை<br>1. Yes	

	உங்களிடம்(மாடுகள்)இருக்கிறதா?	ஆம்<br>-99 Don't know<br>தெரியாது<br>-98 Refused to Answer<br>பதில்கூறமறுத்துவிட்டார்
1.24	Does this household own goats?<br>உங்களிடம்ஆடுகள்இருக்கிறதா?	0. - None<br>எதுவுமில்லை<br>1. Yes<br>ஆம்<br>-99 Don't know<br>தெரியாது<br>-98 Refused to Answer<br>பதில்கூறமறுத்துவிட்டார்
1.25	Does this household own chickens or any other birds?<br>உங்களிடம்கோழிகள்இருக்கிறதா?	0. None<br>எதுவுமில்லை<br>1. Yes<br>ஆம்<br>-99 Don't know<br>தெரியாது<br>-98 Refused to Answer<br>பதில்கூறமறுத்துவிட்டார்
1.26	Does this household have a bank account?<br>உங்கள் குடும்பத்தில் உள்ள யாருக்காவது வங்கி கணக்கு உள்ளதா?	0. No<br>இல்லை<br>1. Yes<br>ஆம்<br>-99 Don't know<br>தெரியாது<br>-98 Refused to Answer<br>பதில்கூறமறுத்துவிட்டார்

Section 2: Household Expenditure and Consumption | பிரிவு 2: வீட்டுசெலவுகள்மற்றும்நுகர்வு

Instruction to data team:

Start with 2.2 in this section if

- the response to 0.18 is '0' and response to 0.19 is '4'

Else, start with 2.0

For every question, display the following below the question:

“Instructionto surveyorஆய்வாளருக்கானகுறிப்புகள்: Write -99 if Don’t know தெரியாது. Write -99 if Refused to Answer-98 பதில்கூறமறுத்துவிட்டார்”

Instructions to surveyor:

ஆய்வாளருக்கானகுறிப்புகள்:

Read out loud:

இதைசத்தமாகபங்குகொள்பவருக்குபடித்துக்காண்பிக்கவும்:

“In this section we shall ask you a few questions about your household’s expenditure and consumption habits. If you are not comfortable answering these questions you may to refuse to answer that particular question, in which case we shall skip that question and move ahead”

“இந்தபிரிவில்உங்கள்வீட்டுசெலவுகள்மற்றும்நுகர்வுபழக்கங்களைகுறித்துநாங்கள்ஒருசிலகேள்விகளைஉங்களிடம்கேட்போம். அப்படிநாங்கள்கேட்கும்ஏதேனும்கேள்விகளுக்குபதில்சொல்லஉங்களுக்குசங்கடமாகஇருந்தால், அதைநீங்கள்எங்களிடம்சொல்லலாம். அப்போதுநாங்கள்அந்தகேள்வியைதவிர்த்துவிட்டுஅடுத்துகேள்விக்குசென்றுவிடுவோம்.”

**Q. No. கே. எண்**	**Question கேள்வி**	**Answer Options பதில்தேர்வுகள்**		**Coding Instructions**
		Expenditure last 30 days கடந்த 30 நாட்களில் செய்யப்பட்ட செலவுகள்		Display the following table
		**Quantity (kgs)** அளவு (கிலோ)	**Value (Rs)** மதிப்பு (ரூ)	
2.0	Rice from PDS, directly from FPS (using all cards owned and used by household) in the last 30 days			Restrict quantity between 0-

	கடந்த 30 நாட்களில்நேரடியா FPS -லிருந்துவாங்கிய PDS அரிசி, ஆய்வாளர் குறிப்பு : ஒன்றுக்கு மேற்பட்ட ரேஷன் கார்டு இருப்பின் சேர்த்து கணக்கிட்டுக்கொள்ளவும்			200, including 0<br><br>Restrict value to positive value, including 0<br><br>If 0 is entered in Quantity then skip to 2.1 and don't ask Value
2.1	Rice from PDS, other sources (purchasing from a friend, gifts, in kind payments etc.) in the last 30 days<br>கடந்த 30 நாட்களில்பிறவழிகளில் வாங்கிய) PDS அரிசி (நண்பரிடம் வாங்கிய , பரிசாகபெற்ற,கூலியாக,சம்பளமாக பெற்ற அரிசி)			Restrict quantity between 0-200, including 0<br><br>Restrict value to positive value, including 0
2.2	Non PDS rice purchased from market in the last 30 days<br>கடந்த 30 நாட்களில் ரேஷன் கடை தவிர வேறு கடையிலிருந்துவாங்கியஅரிசி			Restrict quantity between 0-200, including 0
2.3	Non-PDS rice from gifts, in kind payments in the last 30 days<br>கடந்த 30 நாட்களில்பரிசாகபெற்ற, கூலியாக,சம்பளமாக பெற்ற ரேஷன் கடை அல்லாத அரிசி			Restrict quantity between 0-200, including 0
2.4	Rice from home production in the last 30 days<br>கடந்த 30 நாட்களில்சொந்தமாகவிளைவித்தஅரிசி(சொந்த, குத்தகை மற்றும்			Restrict quantity between 0-200, including 0

	குடும்பத்தினர் நிலத்தில் விளைவித்தஅரிசி)			
2.5	Wheat, atta, other cereals and substitutes purchased through PDS and other means in the last 30 days<br>கடந்த 30 நாட்களில்கோதுமை, கோதுமைமாவு, இதரஉணவுதானியங்கள்மற்றும்அதற்கானமாற்று(கம்பு,கேழ்வரகு,சோளம்,ரவை போன்றவை)பொருட்கள் [ரேஷன் கடை மற்றும் இதர வழியில் வாங்கியவை]			Restrict value to positive value, including 0
2.6	pulses & pulse products purchased through PDS and other means in the last 30 days<br>கடந்த 30 நாட்களில் பருப்புமற்றும் (உ ளுந்து, கடலை பருப்பு,துவரம் பருப்பு போன்ற)பருப்புபொருட்கள் [ரேஷன் கடை மற்றும் இதர வழியில் வாங்கியவை]			Restrict value to positive value, including 0
2.7	milk & milk products purchased through PDS and other means in the last 30 days<br>கடந்த 30 நாட்களில் பால்மற்றும்பால்பொருட்கள்(தயிர், நெய்,வெண்ணை,பன்னீர் போன்றவை)			Restrict value to positive value, including 0
2.8	Salt, sugar & spices purchased through PDS and other means in the last 30 days<br>கடந்த 30 நாட்களில் உப்பு, சர்க்கரைமற்றும்மசாலாப்பொருட்கள்[ரேஷன் கடை மற்றும் இதர வழியில் வாங்கியவை]			Restrict value to positive value, including 0
2.9	edible oil purchased through PDS and other means in the last 30 days<br>கடந்த 30 நாட்களில்சமையல்எண்ணெய் (நல்லெண்ணெய்,கடலை எண்ணெய் பாம் ஆயில் போன்றவை) [ரேஷன் கடை மற்றும் இதர வழியில் வாங்கியவை]			Restrict value to positive value, including 0

2.10	egg, fish & meat in the last 30 days<br>கடந்த 30 நாட்களில்முட்டை, மீன்மற்றும்இறைச்சி			Restrict value to positive value, including 0
2.11	Vegetables, fruits (fresh & dry) in the last 30 days<br>கடந்த 30 நாட்களில்காய்கறி, பழங்கள் (புதிதானதுமற்றும்உலரவைக்கப்பட்டது)			Restrict value to positive value, including 0
2.12	processed food (beverages, packaged, snacks, restaurant meals etc.) in the last 30 days<br>கடந்த 30 நாட்களில்பதப்படுத்தப்பட்டஉணவு (பானங்கள், உரையில்அடைக்கப்பட்டஉணவுகள், தின்பண்டங்கள், ஹோட்டல்உணவுகள்முதலியவை) eg:சிப்ஸ், ஃபான்டா,			Restrict value to positive value, including 0
		Expenditure last 30 days<br>போன30 நாட்களில்செய்யப்பட்டசெலவுகள்		
2.15	Medical expenditures in the last 30 days<br>கடந்த 30 நாட்களில்மருத்துவசெலவுகள்			Restrict value to positive value, including 0
2.18	Any other expenditure in the last 30 days<br>கடந்த 30 நாட்களில்வேறுஏதேனும்செலவுகள்<br>Note to surveyor: Prompt by giving examples of other items "By others we mean.transportation/ luxury items, religious/ cultural events or functions, jewellery, etc."<br><br>ஆய்வாளருக்கானகுறிப்பு: "இதரசெலவுகள்எனில் போக்குவரத்து/ஆடம்பரபொருட்கள்/ மத / கலாச்சார நிகழ்வுகள் அல்லது			Restrict value to positive value, including 0

	செயல்பாடுகள், நகைமுதலியவைபோன்ற இதரசெலவுகளுக்குதேவையானஉதாரணங்களைசொல்லிஅவர்களிடம்இதற்கானபதிலைகேட்கவும்”			

Section 3: PDS | பிரிவு 3: PDS

Instruction to data team:

The questions in this section must **not** be administered if

- the response to 0.18 is '0' and response to 0.19 is '4'

Instructions to the surveyor:

ஆய்வாளருக்கானகுறிப்பு:

Read out loud:

இதைசத்தமாகபங்குகொள்பவருக்குபடித்துக்காண்பிக்கவும்:

“In this section, we shall ask you a few questions about consumption of rice supplied by the Government's Public Distribution System, and your impression of the Public Distribution System. Your responses are important for us to understand the actual use of the PDS supplied rice. Your responses shall be kept confidential and will not be shared with anyone outside of the research team.”

“இந்தபிரிவில், **அரசு பொது விநியோக அமைப்பின் மூலம் வழங்கப்படும் அரிசியை சாப்பிடுவது மற்றும்** அரசு பொது விநியோக அமைப்**பு பற்றிய உங்களது அபிப்பிராயம் குறித்த** ஒரு சில கேள்விகளை **நாங்கள்** உங்களிடம் கேட்போம்.

PDS **அரிசி உண்மையில் எப்படி பயன்படுத்தப்படுகிறது**, **என்பதை குறித்து நாங்கள் புரிந்துக் கொள்வதற்கு உங்கள் பதில் எங்களுக்கு மிகவும் முக்கியமாகும்**. **நீங்கள் சொல்லும் பதில் முற்றிலும் இரகசியமாக பாதுகாக்கப்படும்**, **அதோடு எங்கள் ஆராய்ச்சி குழுவை சாராத யாரிடமும் பகிர்ந்துக் கொள்ளப்படாது**.”

**Q. No.**	**Question கேள்வி**	**Answer Options பதில்தேர்வுகள்**	**Coding Instructions**

கே. எண்			
3.00	How many kilograms of rice is your household eligible to take from FPSs per month using all the ration cards that you own?<br>ரேஷன் கடையிலிருந்து மாதம் ஒன்றுக்கு உங்களுக்கு சொந்தமான அனைத்து ரேஷன் அட்டைகளை பயன்படுத்தி உங்கள் வீட்டிற்கு எத்தனை கிலோ அரிசியை வாங்க முடியும்?	_ _ kilograms<br><br>Write ___ கிலோ<br>-99 if Don't knowதெரியாது<br>Write -99 if Refused to Answer<br>-98<br>பதில்கூறமறுத்துவிட்டார்	Numeric response between 0-150, including 0
3.01	How many kilograms of rice does your household usually take per month from FPSs using all the cards that you own?<br>ரேஷன் கடையிலிருந்து மாதம் ஒன்றுக்கு உங்களுக்கு சொந்தமான அனைத்து ரேஷன் அட்டைகளை பயன்படுத்தி உங்கள் வீட்டிற்கு **வழக்கமாக** எத்தனை கிலோ அரிசியை நீங்கள் வாங்குவீர்கள்?	_ _ kilograms<br><br>Write ___ கிலோ<br>-99 if Don't knowதெரியாது<br>Write -99 if Refused to Answer<br>-98<br>பதில்கூறமறுத்துவிட்டார்	Numeric response between 0-150, including 0<br><br>Should not be more in value than 3.00
3.02	Rice available at FPSs is sometimes of two main kinds - parboiled rice and raw rice. Please tell us how much of each kind of rice your household took from the ration shop last month, using all the cards that you own?<br><br><u>Instruction to surveyor:</u><br>Write 0 if none.<br>அவ்வப்போதுFPS கடைகளில்வழங்கப்படும்அரிசிஇரண்டுவகையைசார்ந்ததாகஇருக்கு	1. _ _ kilograms parboiled rice<br>____ கிலோபுழுங்கலரிசி<br>2. _ _ kilograms raw rice<br>Write ____ கிலோபச்சையரிசி<br>-99 if Don't knowதெரியாது<br>-98 if Refused to Answer<br>பதில்கூறமறுத்துவிட்டார்	Numeric response between 0-150, including 0<br><br>Skip 3.03 if 0 for 2 and skip 3.04 if 0 for 1<br><br>3.02.1+3.02.2 should not be more than 3.00

	ம் - புழுங்கலரிசிமற்றும்பச்சையரிசி. போனமாதம்உங்களுக்கு சொந்தமான அனைத்து ரேஷன் அட்டைகள்மூலம்இந்தஇரண்டுவகையானஅரிசிகளில்எத்தனைஎத்தனை கிலோஉங்கள்வீட்டுக்கு வாங்கினீர்கள்என்றுசொல்லவும்?		
3.03	How do you cook PDS supplied raw rice?<br>PDS - லிருந்துவாங்கியபச்சையரிசியைஎப்படிசமைப்பீர்கள்?<br><br>Note to surveyor:<br>This means rice cooked for meals. Not pongal, or vathal preparations.<br>ஆய்வாளர்குறிப்பு<br>அதாவது, சாதமாக சமைக்கும் முறையை மட்டும் கணக்கிட்டுகிள்ளவும் , பொங்கல், வத்தல் போன்ற பயன்பாட்டிற்கு பயன்படுத்துவதை கணக்கிட்டுக்கொள்ளக் கூடாது.	1.Water draining - where rice is boiled until cooked and the excess water is drained out<br>கஞ்சிவடித்துசமைத்தல் - அரிசியைவேகும்வரை சமைத்துவிட்டுகடைசியில்கூடுதலாகஇருக்கும்கஞ்சியைவடித்தல்<br>2.Watertight - where all water is either evaporated or absorbed by the rice during cooking. No water is drained.<br>கஞ்சிவடிக்காமல்சமைத்தல் - சமைக்கும்போதுஅரிசியுடன்நீர்ஆவியாகிவிடும்அல்லதுஅரிசியேஅதைஇழுத்துக்கொள்ளும்.<br>இதில்கஞ்சிவடிக்கப்படாது<br>3.Pressure cooker/ Rice cooker - no water is drained	Multiple Choices should be allowed

		பிரஷர்குக்கர்/ரைஸ்குக்கர்சமையல் - இதிலும்நீர்வடிக்கப்படாது<br>4. We do not cook raw PDS rice as rice. We use it to make idli/dosa batter. நாங்கள் பச்சரிசியை சமைப்பதில்லை. இட்லி, தோசைக்கு பயன்படுத்துகிறோம்.<br><br>5. We get it from the shop, but do not cook it/consume it at allநாங்கள்ரேஷன்கடைபச்சரிசியைவாங்குகிறோம்ஆனால்சமைப்பதில்லை.<br>-99 Don't know தெரியாது<br>-98 Refused to Answer பதில்கூறமறுத்துவிட்டார்<br>97Other: specify மற்றவை: குறிப்பிடவும்	
3.04	How do you cook PDS supplied parboiled rice? PDS - லிருந்துவாங்கியபுழுங்கலரிசியைஎப்படிசமைப்பீர்கள்?<br><br>Note to surveyor:<br>This means rice cooked for meals. Not pongal, or vathal preparations.<br>ஆய்வாளர்குறிப்பு : அதாவது, சாதமாக	1.Water draining - where rice is boiled until cooked and the excess water is drained out கஞ்சிவடித்துசமைத்தல்-அரிசியைவேகும்வரைசமைத்துவிட்டுகடைசியில்கூடுதலாகஇருக்கும்கஞ்சியைவடித்தல்<br>2.Watertight - where all water is either evaporated or absorbed by the rice	Multiple Choices should be allowed

	சமைக்கும் முறையை மட்டும் கணக்கிட்டுகிள்ளவும் , பொங்கல், வத்தல் போன்ற பயன்பாட்டிற்கு பயன்படுத்துவதை கணக்கிட்டுக்கொள்ளக் கூடாது.	during cooking. No water is drained.<br>கஞ்சிவடிக்காமல்சமைத்தல்- சமைக்கும்போதுஅரிசியுடன்நீர்ஆவியாகிவிடும்அல்லதுஅரிசியேஅதைஇழுத்துக்கொள்ளும்.<br>இதில்கஞ்சிவடிக்கப்படாது<br>3.Pressure cooker/ Rice cooker - no water is drained<br>பிரஷர்குக்கர்/ரைஸ்குக்கர்சமையல்- இதிலும்நீர்வடிக்கப்படாது<br>4. We do not use parboiled PDS rice to cook as rice, We use it to make idli/dosa<br>நாங்கள் புழுங்கலரிசியை சமைப்பதில்லை. இட்லி, தோசைக்கு பயன்படுத்துகிறோம்.<br><br>5. We get it from the shop, but do not cook it/consume it at all.<br><br>நாங்கள்ரேஷன்கடைபுழுங்கலரிசியைவாங்குகிறோம்ஆனால் சமைப்பதில்லை<br><br>-99 Don’t know<br>தெரியாது<br>-98 Refused to Answer	

		பதில்கூறமறுத்துவிட்டார்<br>97Other: specify<br>மற்றவை: குறிப்பிடவும்	
3.05	How much of the PDS rice that you obtain from FPSs do you actually eat, including in the form of rice preparations such as idlis, dosas or any other form?<br>Instruction to surveyor:Write 0 if no rice is eaten.<br>FPS கடையிலிருந்துவாங்கும் PDS அரிசியில்எந்தளவுநீங்கள்சமைத்துசாப்பிடுவீர்கள். இதில்இட்லி, தோசைஅல்லதுஅரிசிமூலம்தயாரிக்கப்படும்வேறுசாப்பாடுபோன்றவற்றையும்சேர்த்துகூறவேண்டும்.<br>ஆய்வாளருக்கானகுறிப்பு: ஒருவேளைரேஷன்அரிசியேஅவர்கள்சாப்பிடவில்லைஎனில் '0' எனஉள்ளிடவும்.	_ _ kilograms<br><br>Write __ கிலோ<br>Write -99 if Don't know தெரியாது<br>Write -98 if Refused to Answer பதில்கூறமறுத்துவிட்டார்	Numeric response between 0-150, including 0<br><br>Should not be more than 3.00
3.06	Approximately how many kilograms of PDS rice do you give away for free per month?<br>Instruction to surveyorenumerator: Write 0 if no rice is given awayaway<br>ஒவ்வொருமாதமும்தோராயமாகஎத்தனைகிலோரேஷன்அரிசியைநீ	_ _ kilograms<br>Write __ கிலோ<br>Write -99 if Don't know தெரியாது<br>Write -98 if Refused to Answer பதில்கூறமறுத்துவிட்டார்	Numeric response between 0-150, including 0

	ங்கள்இலவசமாகமற்றவர்களுக்குகொடுப்பீர்கள்? ஆய்வாளருக்கானகுறிப்பு: ஒருவேளைரேஷன்அரிசியையாருக்கும்இலவசமாககொடுப்பதில்லைஎனில்‘0’ எனஉள்ளிடவும்.		
3.07	Approximately how many kilograms of PDS rice do you sell or exchange for something else per month? Instruction to surveyorenumerator: Write 0 if no rice is sold ஒவ்வொருமாதமும்வேறுஒருபொருளுக்காகதோராயமாகஎத்தனைகிலோரேஷன்அரிசியைநீங்கள்மாற்றிக்கொள்வீர்கள்? ஆய்வாளருக்கானகுறிப்பு: ஒருவேளைரேஷன்அரிசியையாருக்கும்விற்பதில்லைஎனில்‘0’ எனஉள்ளிடவும்.	_ _ kilograms<br><br>Write __ கிலோ<br>-99 if Don’t know<br>Write -99 if Refused to Answerதெரியாது<br>-98<br>பதில்கூறமறுத்துவிட்டார்	Numeric response between 0-150, including 0
3.08	Approximately how many kilograms of PDS rice do you feed your cattle or other animals per month? Instruction to surveyorenumerator: Write 0 if you do not feed PDS rice to animals ஒவ்வொருமாதமும்தோராயமாகஎத்தனைகிலோரேஷன்அரிசியைநீங்கள்கால்நடைஅல்லது	_ _ Kilograms<br><br>Write __ கிலோ<br><br>Write -99 if Don’t know தெரியாது<br>Write -98 if Refused to Answer<br>பதில்கூறமறுத்துவிட்டார்	Numeric response between 0-150, including 0

	வேறுவிலங்குகளுக்குதீ வனமாகபோடுவீர்கள்? ஆய்வாளருக்கானகுறிப்பு: ஒருவேளைரேஷன்அரிசியைவிலங்குகளுக்குதீவனமாககொடுப்பதில்லை எனில்‘0’ எனஉள்ளிடவும்.		
3.09	Do you give your PDS rice for polishing?<br>நீங்கள்வாங்கும்ரேஷன் அரிசிக்குபாலிஷ்போடுவீர்களா?	1. No<br>இல்லை<br>2. Yes<br>ஆம்<br>-99 Don’t know<br>தெரியாது<br>-98 Refused to Answer<br>பதில்கூறமறுத்துவிட்டார்	
3.10	Do you give your PDS rice to the local miller to be ground into rice flour/ batter to make idlis and dosas?<br>நீங்கள்வாங்கும்ரேஷன் அரிசியைஇட்லிமற்றும் தோசைமாவாகஅரைப்பதற்குநீங்கள்மாவரைப்பவரிடம்கொடுப்பீர்களா?	1. No<br>இல்லை<br>2. Yes<br>ஆம்<br>-99 Don’t know<br>தெரியாது<br>-98 Refused to Answer<br>பதில்கூறமறுத்துவிட்டார்	
3.11	How would you describe the quality of the PDS rice that you receive?<br>நீங்கள்வாங்கும்ரேஷன் அரிசியின்தரம்எப்படிஇருக்கும்?	1.Good<br>நன்று<br>2. Average<br>சராசரி<br>3. Poor<br>**மோசம்**<br>-99 Don’t know<br>தெரியாது<br>-98 Refused to Answer<br>பதில்கூறமறுத்துவிட்டார்	After this, go to next section (Section 5) if 0.19 was ‘4’. Else, go to the next question: 3.12
3.12	Does your household take rice from more than one ration shop?	0.No<br>இல்லை<br>1. Yes	If 0/-98/-99, end section

	உங்கள்வீட்டில்ஒன்றுக்கும்மேற்பட்டரேஷன்கடைகளிலிருந்துஅரிசிவாங்குவீர்களா?	ஆம்<br>-99 Don't know<br>தெரியாது<br>-98 Refused to Answer<br>பதில்கூறமறுத்துவிட்டார்	
3.13	Why does your household take rice from more than one ration shop?<br>Instruction to surveyor: Mark as many as applicable<br>எதற்காகஉங்கள்வீட்டில் ஒன்றுக்கும்மேற்பட்டரேஷன்கடைகளிலிருந்துஅரிசிவாங்குகிறீர்கள்?	1. The PDS shop we have been allocated does not have enough stock எங்களுக்குஒதுக்கிதரப்பட்டுள்ளரேஷன் கடையில்போதுமானஅளவுபொருட்கள் கிடைப்பதில்லை.<br>2. The PDS shop we have been allocated is not open எங்களுக்குஒதுக்கிதரப்பட்டுள்ளரேஷன் கடைதிறக்கப்படுவதில்லை<br>3. The PDS shop we have been allocated is too far எங்களுக்குஒதுக்கிதரப்பட்டுள்ளரேஷன் கடைவெகுதூரத்தில் இருக்கிறது<br>4. We live in a joint household and each family unit has been allocated to a different ration shop எங்கள்வீட்டில்நாங்கள்கூட்டுகுடும்பமாக வசிக்கிறோம். மேலும்ஒவ்வொருகுடும்பத்திற்கும்ஒருரேஷன்கடைஒதுக்கப்பட்டுள்ளது	

		5. We don’t get enough rice from one ration card ஒரு ரேஷன் கார்டில் இருந்து போதுமான அரிசி கிடைப்பதில்லை..<br>-99 Don’t know தெரியாது<br>-98 Refused to Answer பதில்கூறமறுத்துவிட் டார்<br>97 Other - please specify மற்றவை-குறிப்பிடவும்	

Section 4: For non-PDS participants

பிரிவு 4: ரேஷன்கடையில்பொருள்வாங்காதபங்குபெறும்நபர்களுக்கு

Instruction to data team:

This section must only be administered if the response to 0.18 iss ‘0’ AND response to 0.19 is ‘4’

Q. No. கே. எண்	Question கேள்வி	Answer Options பதில்தேர்வுகள்	Coding Instructions
4.00	Why do you not consume PDS rice from the ration shop? நீங்கள்ஏன்ரேஷன்கடையிலிருந்துஅரிசியைவாங்குவதில்லை?	1. Our allocated ration shop is far away எங்களுக்குஒதுக்கப்பட்டுள்ளரேஷன்கடைவெகு தொலைவில்இருக்கிறது<br>2. Our allocated ration shop is always closed/ shop keeper is never available எங்களுக்குஒதுக்கப்பட்டுள்ளரேஷன்கடையிலுள்ள கடைக்காரர்எப்போதுகடையைதிறப்பதில்லை/க	Multiple choice

		டைதிறக்கப்படுவதில்லை<br>3. The PDS rice is of very poor quality<br>ரேஷன்அரிசிமிகவும்மோசமாகஇருக்கிறது<br>-99 Don't know<br>தெரியாது<br>-98 Refused to Answer<br>பதில்கூறமறுத்துவிட்டார்<br>97 Other - please specify<br>மற்றவை-குறிப்பிடவும்	
4.01	Where does your household get rice for consumption from?<br>உங்கள்வீட்டிற்குஎங்கிருந்துஅரிசியை வாங்குகிறீர்கள்?<br><br>Instructions to Surveyor: Select all applicable options<br>ஆய்வாளருக்கானகுறிப்புகள்: பொருந்தக்கூடியஅனைத்துதேர்வுகளையும்செலக்டுசெய்யவும்.	1. Market/ corner store<br>கடையில்/மார்க்கெட்டில்<br>2. We get non-PDS rice from other sources (not market/corner store)<br>ரேஷன் அல்லாத வேறு அரிசியை வேறு வழிகளில் வாங்குகிறோம் (கடையில்/மார்க்கெட்டில் அல்ல<br><br>3. We grow rice<br>அரிசியைநாங்களேவிளைவிக்கிறோம்<br>4. We get PDS rice from other sources<br>ரேஷன்அரிசியைவேறுவழியாகவாங்குகிறோம்.<br>5.<br>-99 Don't know<br>தெரியாது<br>-98 Refused to Answer<br>பதில்கூறமறுத்துவிட்டார்<br>97 Other - please specify<br>மற்றவை-குறிப்பிடவும்	Mark all applicable options

Section5: Supplementation and health of women and children

பிரிவு5: பெண்கள் மற்றும் குழந்தைகளுக்கான கூடுதல் ஊட்டச்சத்தும் உடல் ஆரோக்கியமும்

_ Instructions to the surveyor:

ஆய்வாளருக்கானகுறிப்புகள்:

Read out loud:

இதைசத்தமாகபங்குகொள்பவருக்குபடித்துக்காண்பிக்கவும்:

“We would now like to ask you some personal questions about the health and medical supplemnttaion that you and other women in the household take.We will also be asking you some questions about the children in the household. Some questions may be sensitive; could we speak somewhere privately?”

“இப்போதுஉங்களுடைய மற்றும் உங்கள் வீட்டில் உள்ள மற்ற பெண்கள்உடல்ஆரோக்கியம்குறித்தும்மற்றும்நீங்கள்சாப்பிட்டுவரும்மருந்துமாத்திரைகள்குறித்தும்ஒருசிலகேள்விகளைநாங்கள்உங்களிடம்கேட்கஇருக்கிறோம். உங்கள்வீட்டிலுள்ளகுழந்தைகளைபற்றியும்சிலகேள்விகளைநாங்கள்கேட்போம்.இதில்சிலகேள்விகள்அந்தரங்கமானதாகவும்இருக்கும். ஆகவே, வேறுஎங்காவதுதனிமையானஇடத்தில்போய்பேசலாமா?”

The questions in this section will be directed to a knowledgeable person in the household. For children aged 6 months- 59 months, and women aged 12-17 encourage the respondent to ask their guardian/ mother for help.

இந்தகேள்விகளைவீட்டைப்பற்றிநன்குஅறிந்துள்ளஒருவரிடம்தான்கேட்கவேண்டும். 6 மாதங்கள்முதல் 59 மாதங்கள் வயதுவரையுள்ளகுழந்தைகளுக்கும்மற்றும் 12 - 17 வயதுக்குட்பட்டபெண்களுக்கும்அவர்களதுஅம்மா/பெண்காப்பாளரின்உதவியைகேட்கும்படிஅவர்களைகேட்டுக்கொள்ளவும்.

For women other than the respondent, encourage the respondent to ask the woman for help if she is available.

பதில் அளிப்பவர் தவிர மற்ற பெண்களுக்கு, அவர் இருந்தால், அந்த பெண்மணி உதவியை கேட்பதற்கு பதிலளிப்பாளரை ஊக்கப்படுத்துங்கள்.

Instruction to data team:

தரவுக்குழுவுக்கானகுறிப்பு:

The questions in the table (5.00A to 5.00F) should be displayed in the form of a roster. All eligible members from the household (i.e.- women 12-40 years and children 6 months-59 months5 years) who still living in the household (response to 0.22=1) need to be covered.

Q. No.<br>கே. எண்	Question<br>கேள்வி	Answer Options<br>பதில்தேர்வுகள்	Coding Instructions
	ID of member<br>உறுப்பினரின்அடையாளஎண்		From household roster
	Name of member<br>உறுப்பினரின்பெயர்		From household roster
5.00 A.1	Has [name] consumed iron-folic acid (IFA) tablets during the last 7 days?.<br>சென்ற 7 தினங்களில் [பெயர்] இரும்பு-போலிக்அமில (IFA) சத்துமாத்திரைகளைசாப்பிட்டாரா?	0. No<br>இல்லை<br>1. Yes<br>ஆம்<br>-99 Don't know<br>தெரியாது<br>-98 Refused to Answer<br>பதில்கூறமறுத்துவிட்டார்	Skip to 5.00B.1 if 0/-99/-98<br>Go to 5.00A.2 if 1
5.00 A.2	Where was the supplementation consumed?<br>இந்தகூடுதல்(இரும்பு-போலிக்அமில (IFA) மருந்து / மாத்திரையைஎங்குசாப்பிட்டார்?	1 Home (consumed at home but bought in a medical store etc.)<br>வீட்டில் (வீட்டில் சாப்பிட்டார் ஆனால் தனியார் மருந்து கடையில் வாங்கியது)<br>2 Angwadi center	

		அங்கன்வாடி மையத்தில்<br>3 School பள்ளிக்கூடத்தில்<br>4 Public Health Center பொதுசுகாதார மையத்தில்<br><br>5 Private hospital/clinic தனியார் மருத்துவமனை/ கிளினிக்<br><br>97 Other (specify)<br>மற்றவை - குறிப்பிடவும்<br>-99 Don't know தெரியாது<br>-98 Refused to Answer பதில்கூறமறுத்துவிட்டார்	
5.00 B.1	Has [name] consumed liquid iron drops / iron tablets during the last 7 days சென்ற7 தினங்களில் [பெயர்] இரும்புசத்துசொட்டுமருந்து / இரும்புசத்துமாத்திரைகளைசாப்பிட்டாரா?	0. No இல்லை<br>1. Yes ஆம்<br>97 Other (specify) மற்றவை (குறிப்பிடவும்)<br>-99 Don't know தெரியாது<br>-98 Refused to Answer பதில்கூறமறுத்துவிட்டார்	Skip to 5.00C.1 if 0/-99/-98<br>Go to 5.00B.2 if 1
5.00 B.2	Where was the supplementation consumed?	1. Home (consumed at	

	இந்தகூடுதல்இரும்புசத்து சொட்டுமருந்து / இரும்புசத்துமருந்து / மாத்திரையைஎங்குசாப்பிட்டார்?	home but bought in a medical store etc.) வீட்டில் (வீட்டில் சாப்பிட்டார் ஆனால் தனியார் மருந்து கடையில் வாங்கியது)<br>2. Angwadi center அங்கன்வாடி மையத்தில்<br>3. School பள்ளிக்கூடத்தில்<br>4. Public Health Center பொதுசுகாதார மையத்தில்<br>5. Private hospital/clinic தனியார் மருத்துவமனை/ கிளினிக்<br>97 Other (specify) மற்றவை - குறிப்பிடவும்<br>-99 Don't know தெரியாது<br>-98 Refused to Answer பதில்கூறமறுத்துவிட்டார்	

5.00 C.1	Has [name] consumed vitamin A capsules<br>during the last 6 months?<br>சென்ற6 மாதத்தில் [பெயர்] விட்டமின் -ஏமாத்திரைகளைசாப்பிட்டாரா?	0. No<br>இல்லை<br>1. Yes<br>ஆம்<br>-99 Don't know<br>தெரியாது<br>-98 Refused to Answer<br>பதில்கூறமறுத்துவிட்டார்	Skip to 5.00D.1 if 0/-99/-98<br>Go to 5.00C.2 if 1
5.00 C.2	Where was the supplementation consumed?<br>இந்தகூடுதல்விட்டமின் -ஏமருந்து / மாத்திரையைஎங்குசாப்பிட்டார்?	1. Home (consumed at home but bought in a medical store etc.) வீட்டில் (வீட்டில் சாப்பிட்டார் ஆனால் தனியார் மருந்து கடையில் வாங்கியது)<br>2. Anganwadi center அங்கன்வாடி மையத்தில்<br>3. School பள்ளிக்கூடத்தில்<br>4. Public Health Center<br>பொதுசுகாதார மையத்தில்<br>5. Private hospital/clinic	

		தனியார் மருத்துவமனை/ கிளினிக்<br><br>97 Other (specify)<br>மற்றவை - குறிப்பிடவும்<br>-99 Don't know<br>தெரியாது<br>-98 Refused to Answer<br>பதில்கூறமறுத் துவிட்டார்	
5.00 D.1	Has [name]<br>Taken deworming pills during the last **year**?<br>சென்றவருடத்தில் [பெயர்] பூச்சிமாத்திரைகளைசாப் பிட்டாரா?	0. No<br>இல்லை<br>1. Yes<br>ஆம்<br>-99 Don't know<br>தெரியாது<br>-98 Refused to Answer<br>பதில்கூறமறுத் துவிட்டார்	Skip to 5.00E if 0/-99/-98<br>Go to 5.00D.2 if 1
5.00 D.2	Where was the supplementation consumed?<br>இந்தபூச்சிமாத்திரைகளை எங்குசாப்பிட்டார்?	1. Home (consumed at home but bought in a medical store etc.) வீட்டில் (வீட்டில் சாப்பிட்டார் ஆனால் தனியார் மருந்து கடையில் வாங்கியது)<br>2. Angwadi center	

		அங்கன்வாடி மையத்தில்<br>3. School பள்ளிக்கூடத்தில்<br>4. Public Health Center பொதுசுகாதார மையத்தில்<br><br>5. Private hospital/clinic தனியார் மருத்துவமனை/ கிளினிக்<br><br>97 Other (specify) மற்றவை - குறிப்பிடவும்<br>-99 Don't know தெரியாது<br>-98 Refused to Answer பதில்கூறமறுத்துவிட்டார்	
5.00 E	Has [name] consumed other vitamin pills/syrup during the last month/30 days? Check all that apply (at prompting of surveyor).<br>சென்றமாதம்/30 நாட்களில் [பெயர்] இதரவிட்டமின்மாத்திரைகளை/சிரப்பினைசாப்பிட்டாரா?<br>பொருந்தும் எல்லாவற்றையும் குறிக்கவும் (சர்வேயர் ஆப்ஷன்களை சொல்லி கேட்கவும்)	0. Noஇல்லை<br>1. Iodine ஐயோடின்<br>2. Vitamins (unspecified) விட்டமின்கள் (குறிப்பாக சொல்லப்படாத்து)<br>3. Mineral (unspecified) மினரல்(குறிப்பாகசொல்லப்படாத்து)	Allow multiple options to be selected<br><br>Shouldn't allow 0/-98/-99 and any other option to be selected together.

		4. Vitamins and minerals (unspecified) விட்டமின்மற்றும்மினரல் (குறிப்பாக சொல்லப்படாத்து)<br>97 Other (specify) மற்றவை (குறிப்பிடவும்)<br>-99 Don't know தெரியாது<br>-98 Refused to Answer பதில்கூறமறுத்துவிட்டார்	
5.00 F	Has [name] consumed fortified meals during the last month/30 days?<br>கடந்த30 நாட்களில் [பெயர்] ஊட்டச்சத்து(ஃபார்ட்டிஃபைட்டு) நிறைந்தசாப்பாட்டைசாப்பிட்டாரா?	0. No<br>இல்லை<br>1. Yes<br>ஆம்<br>-99 Don't know<br>தெரியாது<br>-98 Refused to Answer<br>பதில்கூறமறுத்துவிட்டார்	Only ask this question for children between 6 months and 59 months.

Instructions to the surveyor:

ஆய்வாளருக்கானகுறிப்புகள்:

Read out loud:

இதை சத்தமாக பங்குகொள்பவருக்கு படித்துக்காண்பிக்கவும்:

"We would now like to ask you about fortified foods. Fortified foods are those that have added vitamins and minerals to make them healthier and help decrease various deficiencies such as iron, vitamin A and D and iodine. It includes palmoil supplied through the PDS system, and iodized salt."

“இப்போதுநாங்கள்உங்களிடம்(ஃபார்ட்டிஃபைட்டு) ஊட்டச்சத்துநிறைந்தஉணவுகுறித்துகேள்விகேட்கஇருக்கிறோம். (ஃபார்ட்டிஃபைட்டு) ஊட்டச்சத்துநிறைந்தஉணவுஎன்றால், அதுஇரும்பு, விட்டமின் -ஏமற்றும்விட்டமின் -டிமற்றும்ஐயோடின்பற்றாக்குறைகளைநீக்கிஒருவரைஆரோக்கியமாக மாற்றுவதற்காகஉணவில்கூடுதல்விட்டமின்கள்மற்றும்மினரல்களைசேர்த்துதாயரிக்கப்பட்டுள்ளஉணவாகும்.”

Q. No.<br>கே. எண்	Question<br>**கேள்வி**	Answer Options<br>**பதில்தேர்வுகள்**	Coding Instructions
5.00	Did you know what fortified foods are before I mentioned it to you?நான் ஃபார்ட்டிஃபைட்டு உணவுகளை குறிப்பிடுவதற்கு முன் அது என்ன என்பது உங்களுக்கு தெரியுமா?	0. No<br>இல்லை<br>1. Yes<br>ஆம்	
5.01	Have members of this household consumed fortified food items during the last week/7 days?<br>சென்றவாரத்தில் / 7 தினங்களில்இந்தகுடும்பத்தைசேர்ந்தஉறுப்பினர்கள் (ஃபார்ட்டிஃபைட்டு) ஊட்டச்சத்துநிறைந்தஉணவைசாப்பிட்டார்களா?	0. No<br>இல்லை<br>1. Yes<br>ஆம்<br>-99 Don’t know<br>தெரியாது<br>-98 Refused to Answer<br>பதில்கூறமறுத்துவிட்டார்	If 0/-98/-99 then move to next section
5.02	What kind of fortified food items were consumed during the last week/7 days?<br>சென்றவாரத்தில் / 7 தினங்களில்எவ்வகை (ஃபார்ட்டிஃபைட்டு) ஊட்டச்சத்துநிறைந்தஉணவுகளைசாப்பிட்டார்கள்?	1. Rice<br>அரிசி<br>2. Double fortified salt (DFS)<br>இரட்டிப்புஊட்டச்சத்துநிறைந்த(ஃபார்ட்டிஃபைட்டு) உப்பு (DFS)<br>3. Iodized salt	Allow multiple options to be selected

	Instruction to surveyor: Read the options out loud and select all that apply.<br>ஆய்வாளருக்கானகுறிப்புகள்:<br>அனைத்துதேர்வுகளையும் சப்தமாக வாசிக்கவும் பொருந்தக்கூடியஅனைத்துதேர்வுகளையும்செலக்டு செய்யவும்.	ஐயோடின்கலந்தஉப்பு<br>4. Fortified meals in school<br>பள்ளிக்கூடத்தில்வழங்கப்படும்ஊட்டச்சத்துநிறைந்த(ஃபார்ட்டிஃபைட்டு) சாப்பாடு<br>5. Cooking oils<br>சமையல்எண்ணெய்<br>6. Breakfast cereals<br>காலைஉணவுதானியங்கள்<br>7. Milk and milk products<br>பால்மற்றும்பால்பொருட்கள்<br>97 Other: Please specify<br>மற்றவை: குறிப்பிடவும்<br>-99 Don't know<br>தெரியாது<br>-98 Refused to Answer<br>பதில்கூறமறுத்துவிட்டார்	

Section 6: Health outcomes

பிரிவு 6: உடல்ஆரோக்கியவிளைவுகள்

Instructions to the surveyor:

Read: "Now I am going to be asking you questions about the health of women between the age of 12-40 and children between the age of 6 months to 59 months."

ஆய்வாளருக்கானகுறிப்புகள்:

இதைசத்தமாகபங்குகொள்பவருக்குபடித்துக்காண்பிக்கவும்:நான் இப்பொழுது12 மற்றும் 40 வயதுக்குட்பட்டஅனைத்துவீட்டுப்பெண்களுக்கும்மற்றும் 6 மாதங்கள்மற்றும் 5 வயதுக்குட்பட்டகுழந்தைகள் பற்றிய கேள்விகள் கேட்க போகிறேன்

Instructions to data team:

6.00A to 6.00H should be asked as a roster for all eligible membersc

Q. No. கே. எண்	Question கேள்வி	Answer Options பதில்தேர்வுகள்	Coding Instructions
	Name of the member<br>குடும்ப உறுப்பினர் பெயர்<br>ID of member<br>உறுப்பினரின்அடையாள எண்		Display the name and ID of each member in the roster before asking 6.00A
6.00A	In general, would you describe [name] 's health as excellent, very good, good, fair, or poor?பொதுவாக, [பெயர்] - வின்உடல்ஆரோக்கியம்எப்படி விவரிப்பீர்கள் ?பிரமாதம், மிகநன்று, நன்று, பரவாயில்லை, அல்லதுமோசம்இதில் எதை குறிப்பிடுவீர்கள் ?	1Excellent<br>பிரமாதம்<br>2 Very Good<br>மிகநன்று<br>3 Good<br>நன்று<br>4 Fair<br>பரவாயில்லை<br>5 Poor<br>மோசம்<br>-99 Don't know<br>தெரியாது<br>-98 Refused to answer<br>பதில்கூறமறுத்துவிட்டார்	
6.00B	In the last two weeks, did [name] have<br>Malaria<br>கடந்த இரண்டு வாரங்களில் [பெயர்] - விற்கு மலேரியா வந்ததா?	0. No<br>இல்லை<br>1. Yes<br>ஆம்<br>-99 Don't know<br>தெரியாது<br>-98 Refused to anser<br>பதில்கூறமறுத்துவிட்டார்	
6.00C	In the last two weeks, did [name] have fever (different from malaria)	0. No<br>இல்லை<br>1. Yes<br>ஆம்	

	கடந்த இரண்டு வாரங்களில் [பெயர்] -விற்கு மலேரியாஅல்லாதவேறுகாய்ச்சல்ஏதேனும் ஏற்பட்டதா?	-99 Don't know<br>தெரியாது<br>-98 Refused to anser<br>பதில்கூறமறுத்துவிட்டார்	
6.00D	In the last two weeks, did [name] have diarrhea<br>கடந்த இரண்டு வாரங்களில் [பெயர்] -விற்கு வயிற்றுபோக்கு ஏற்பட்டதா?	0. No<br>இல்லை<br>1. Yes<br>ஆம்<br>-99 Don't know<br>தெரியாது<br>-98 Refused to anser<br>பதில்கூறமறுத்துவிட்டார்	
6.00E	In the last two weeks, did [name] excessive or prolonged fatigue / weakness<br>கடந்த இரண்டு வாரங்களில் [பெயர்] -விற்கு தொடர்ச்சியானசோர்வோ / களைப்போ ஏற்பட்டதா?	0. No<br>இல்லை<br>1. Yes<br>ஆம்<br>-99 Don't know<br>தெரியாது<br>-98 Refused to anser<br>பதில்கூறமறுத்துவிட்டார்	
6.00F	In the last two weeks, did [name] suffer from Intestinal worms<br>கடந்த இரண்டு வாரங்களில் [பெயர்] -விற்கு குடல்புழுதாக்கம்ஏற்பட்டதா?	0. No<br>இல்லை<br>1. Yes<br>ஆம்<br>-99 Don't know<br>தெரியாது<br>-98 Refused to anser<br>பதில்கூறமறுத்துவிட்டார்	
6.00G	How many days did [name] have to skip [name]'s normal	___daysநாட்கள்	Numeric entry. Restrict to 0-7

	activities in the past week due to any illness?<br>கடந்த வாரத்தில் [பெயர்] -விற்கு தன்னுடையவழக்கமானசெயல்களைஎத்தனைநாட்களுக்குசெய்யாமல்இருக்கவேண்டியசூழ்நிலை ஏற்பட்டது?	-99 Don't know<br>தெரியாது<br>-98 Refused to Answer<br>பதில்கூறமறுத்துவிட்டார்	i.e.- if days is selected then when days is entered, the entry should be restricted to 0-7. (right now, it seems to not allow don't know and refused to answer)
6.00H	Has [name] been officially diagnosed with anemia?<br>இதுவரை[பெயர்] -விற்குஇரத்தசோகை நோய்இருக்கிறதாஎன்றுஎந்தமருத்துவராவதுகண்டறிந்துள்ளாரா ?	0. No<br>இல்லை<br>1. Yes<br>ஆம்<br>-99 DK<br>தெரியாது<br>-98 RA<br>பதில்கூறமறுத்துவிட்டார்	

<u>Instructions to the surveyor:</u>

<u>ஆய்வாளருக்கானகுறிப்புகள்:</u>

Read out loud: "Now I am going to be asking you some general questions."
இதைசத்தமாகபங்குகொள்பவருக்குபடித்துக்காண்பிக்கவும்:இப்போது நான்சில பொதுவான கேள்விகள் கேட்க போகிறேன்

**Q. No. கே.எண்**	**Question கேள்வி**	**Answer Options பதிலுக்கானதேர்வுகள்**	**Coding Instructions**
6.01	What is the importance of vitamins (such as Vit A, B etc.) and minerals (such as iron, iodine etc.)? (Select all that apply but do NOT prompt)	1. They help prevent diseases அவைநோய்ஏற்படுவதைதடுக்கஉதவும்<br>2. They give us energy	Allow multiple choices to be selected.

	விட்டமின்கள் (அதாவதுவிட்டமின் -ஏ, பிமுதலியவை) மற்றும்மினரல்கள் (அதாவதுஇரும்புமற்றும்ஜ யோடின்போன்றவை) முக்கியத்துவம்என்ன? (பொருந்தக்கூடியஅனைத் தையும்தேர்வுசெய்யவும், ஆனாலும்அவரிடம்தேர்வுக ள்எதையும்படித்துக்காண்பி க்கவேண்டாம்)<br><br>Instructions to surveyor:<br><br>Do NOT read the options out loud. Select all that apply but do NOT prompt.<br>ஆய்வாளருக்கானகுறிப்பு: தேர்வுகளைபங்குகொள்பவ ருக்குபடித்துக்காண்பிக்க வேண்டாம். பொருந்தக்கூடியஅனைத் தையும்தேர்வுசெய்யவும்.	அதுநமக்குசக்திதருகிற து<br>3. They help staying focused/concentrated in school<br>பள்ளிக்கூடத்தில்நல்ல படிபாடத்தைகவனிக்க உதவும்<br>4. They prevent or treat anemia<br>அதுஇரத்தசோகையை தடுக்கும்அல்லதுகுண மாக்கும்<br>5. Help with healthier pregnancies<br>கர்பகாலத்தில்ஆரோக் கியமாகஇருக்கஉதவும்<br>6. No importance<br>எந்தமுக்கியத்துவமும் இல்லை<br>-99 Don't know<br>தெரியாது<br>-98 Refused to Answer<br>பதில்கூறமறுத்துவிட்டார்<br>97 Other: Please specify<br>மற்றவை: குறிப்பிடவும்	Should not allow "No importance"/"Don't know"/"Refused to answer" with other answer options. Error message if selected: You can't select No importance"/"Don't know"/"Refused to answer" with other options
6.02	Do you know what anemia is?<br>இரத்தசோகைஎன்றால்என் னவென்றுஉங்களுக்குதெரி யுமா?	0 No<br>இல்லை<br>1 Yes<br>ஆம்<br>-99 Don't know<br>தெரியாது	Skip 6.03-6.05 if 0/-98/-99

		-98 Refused to Answer<br>பதில்கூறமறுத்துவிட்டார்	
6.03	What are the primary causes of anemia?<br>இரத்தசோகைஏற்படுவதற்குமுக்கியகாரணங்கள்என்னென்ன?	1. Not enough iron in diet<br>உணவில்போதுமானஇரும்புசத்துஇல்லாமல்இருப்பது<br>2. Malaria<br>மலேரியா<br>3. Intestinal worms<br>குடல்புழுக்கள்<br><br>97 Other: Please specify<br>மற்றவை: குறிப்பிடவும்<br>-99 Don't know<br>தெரியாது<br>-98 Refused to Answer<br>பதில்கூறமறுத்துவிட்டார்	Allow multiple answers to be selected
6.04	What are the consequences of anemia?<br>இரத்தசோகையின்விளைவுகள்என்னென்ன?	1. Tiredness<br>சோர்வு<br>2. Fatigue<br>களைப்பு<br>3. shortness of breath<br>மூச்சிறைத்தல்<br><br>97 Other: Please specify<br>மற்றவை: குறிப்பிடவும்<br>-99 Don't know<br>தெரியாது<br>-98 Refused to Answer<br>பதில்கூறமறுத்துவிட்டார்	Allow multiple answers to be selected
6.05	What can be done to treat anemia?<br>இரத்தசோகைநோயைகுணப்படுத்தஎன்னென்னசெய்யவேண்டும்?	1. Take iron/IFA tablets<br>இரும்புசத்து/IFA மாத்திரைகளைசாப்பிடவேண்டும்<br>2. Eat more meat<br>இறைச்சியைஅதிகம்சாப்பிடவேண்டும்<br>3. Eat more fruit/vegetables	Allow multiple answers to be selected

		அதிகமானகாய்கறிகளையும்பழங்களையும்சாப்பிட வேண்டும்<br>97 Other: Please specify<br>மற்றவை: குறிப்பிடவும்<br>-99 Don’t know<br>தெரியாது<br>-98 Refused to Answer<br>பதில்கூறமறுத்துவிட்டார்	

Section 7: Cognitive outcomes for children

Instructions to data team:

Only enter this section if there is an eligible child in the hh who is stil living there (i..e- for at least one member in the hh, in pre-fill mem_elig_child=1 and 0.22=1)

Q. No. கே. எண்	Question கேள்வி	Answer Options பதிலுக்கானதேர்வுகள்	Coding instructions for the data team
F5.1	How many books are there in the household (including school books, but not other books meant for children such as picture books)? இந்தவீட்டில்எத்தனைபுத்தகங்கள்இருக்கிறது (பள்ளிக்கூடபுத்தகங்கள்உட்பட, ஆனாலும்புகைப்பட /பொம்மை படப்புத்தகங்கள்போன்றகுழந்தைகளுக்கான இதரபுத்தகங்கள்சேர்க்காமல் கூறவும்	None | எதுவுமில்லை ..1<br>1-2 - 2<br>3-5 - 3<br>6 or more | 6 அல்லதுஅதற்குமேல் ..4<br>Don't know / don't remember ..98<br>98. தெரியாது / நினைவில்லை	
F5.2	How many magazines and newspapers are there in the household?<br>இந்தவீட்டில்எத்தனைபத்திரிகைகளும்செய்தித்தாள்களும்இருக்கின்றன?	None | எதுவுமில்லை ..1<br>1-2 - 2<br>3-5 - 3<br>6 or more | 6 அல்லதுஅதற்குமேல் ..4<br>Don't know / don't remember ..98<br>தெரியாது / நினைவில்லை	

Instruction to data team:

Repeat the next set of questions in this section for all eligible children in the hh (mem_elig_child=1 in the prefill) who are still living in the hh (0.22=1)

Q. No. கே. எண்	Question கேள்வி	Answer Options பதிலுக்கானதேர்வுகள்	Coding instructions for the data team
	Name of the member<br>குடும்ப உறுப்பினர் பெயர்<br>ID of member<br>உறுப்பினரின்அடையாள எண்		Display the name of the eligible children in the household (mem_elig_child=1)<br><br>Don't allow the same child's name to be selected for subsequent children in the roster.
F5.21	**Date of Birth**<br>பிறே்ேபேதி	___|____| / |____|____| / 20|____|____| d d m m yy	
7.00	**Age in months**<br>வயது (மாதக்கணக்கில்)		Display age of the child in months, by calculating age from F5.21
7.01	**Is [name] eligible?**<br>இவர் (பெயர்) நம் ஆய்வுக்கு தகுதியுடையவரா?	0 No<br>இல்லை<br>1 Yes<br>ஆம்	Check: If 1 entered here then value in 7.00 should be between 6-59 months. If not, how an error message.

			Check if 0 entered here then value in 7.00 should be between lesser than 6 or more than 59 months.<br><br>If 0 then go to the next person in the roster.<br><br>If 1 then go the 7.02
7.02	Is this child available for the survey right now?<br><br>பேட்டிகாணும் போது குழந்தை இருந்தாரா?	0 No<br><br>இல்லை<br><br>1 Yes<br><br>ஆம்	If 0 then go to the next person in the roster.<br><br>If 1 then continue to Section 7A/7B

Calculate age based on F5.21 and accordingly:

- Ask Section 7A for all the children between the age of 6 months to 36 months
- ask Section 7B for all children between 36 months 60 months,
- Section 7C must be administered for all the children and should follow Section 7A/7B in the roster.

For example: if there are 2 children in the household: one who is 9 months old and the other who is 4 years old then first Section 7B and section 7C must be administered for the older child and then Section 7A and 7C must be administered for the younger child. Please note that for children who are 36 months old, both Section 7A and 7B must be administered.

Section 7A: DMC

Instructions to surveyors:

The DMC-III is an 86-item questionnaire measuring gross motor, fine motor, and language development in children. The majority of items will be assessed using a parent report, with the exception of language items 7.21 and 7.24 to 7.33. Interviews should be conducted with the mother or primary caregiver of the child.

<table>
<tr><th>Q | கே</th><th>Information requested | வேண்டப்படும்தகவல்</th><th colspan="2">Data | தரவு</th></tr>
<tr><td>D1.1</td><td>Nickname of the child: ____________________
குழந்தையுடன்செல்லபெயர்
Instruction to data team: Only allow text entry</td><td colspan="2"></td></tr>
<tr><td>D2.</td><td>Date of the interview / observation
நேர்காணல்/கண்காணிப்புந டைபெறும்தேதி</td><td colspan="2">|____|____| / |____|____| / 20|____|____|
d d m m
yy</td></tr>
<tr><td>D3.2</td><td colspan="2">Respondent's relationship to the child
குழந்தையுடன்பதில்அளிப்பவரின்உறவுமுறை
1 = Mother, 2 = Father, 3 = Other adult, family member ≥15 years of age
1=அம்மா, 2=அப்பா, 3=இதரவயதுமுதிர்ந்தகுடும்பஉறுப்பினர்≥ 15 வயது</td><td>|___| Code | குறியீடு</td></tr>
<tr><td>D4.1</td><td colspan="2">Language most often spoken to the child
குழந்தையுடன்அடிக்கடிபேசப்படும்மொழி
1 = Tamil தமிழ்- , 2 = English இங்கிலீஷ், 3 = Hindi இந்தி, 4 = Others</td><td>|___| Code | குறியீடு</td></tr>
<tr><td>D4.2</td><td colspan="2">Language child most often speaks</td><td>|___| Code | குறியீடு</td></tr>
</table>

	குழந்தைஅடிக்கடிபேசும்மொழி<br><br>1 = **Tamil தமிழ்-, 2 = English இங்கிலீஷ், 3 = Hindi இந்தி 4 = Others**	
**D4.3**	Total number of languages spoken to the child by family, friends, and others<br><br>குடும்பம், நண்பர்கள்மற்றும்மற்றவர்கள்மூலமாககுழந்தையுடன்பேசப்படும்மொழிகளின்எண்ணிக்கை	|___| **Code | குறியீடு**

**Codes for the Response column:**<br><br>**பதில்காலம்/பத்திக்கானகுறியீடுகள்:**<br><br>**0 = respondent said child has not yet started doing the activity**<br><br>**0 = குழந்தைஎந்தவொருநடவடிக்கையிலும்இன்னும்ஈடுபடதுவங்கவில்லைஎன்றுபங்காளர்தெரிவித்தார்**<br><br>**1 = respondent said child has been able to do the activity in the past 1 month but not continually**<br><br>**1 = கடந்தஒருமாதத்தில் குழந்தையால்நடவடிக்கையில்ஈடுபடமுடிந்ததுஆனால்அதனால்தொடர்ச்சியாகஈடுபடமுடியவில்லைஎனபங்காளர்தெரிவித்தார்**<br><br>**2 = respondent said child has been able to do the activity continually for the past 1 month**<br><br>**2 = குழந்தையால்தொடர்ந்துகடந்தஒருமாதத்தில் நடவடிக்கையில்ஈடுபடமுடிந்ததுஎனபங்காளர்தெரிவித்தார்**<br><br>**9 = mother/caregiver does not know / does not remember / has not observed**	**Codes for the Observation column :**<br><br>**0 = interviewer observed that the child cannot do the activity**ஆய்வாளர் கண்காணித்த போது குழந்தை எந்தவொரு நடவடிக்கையிலும் ஈடுபட வில்லை<br><br>**1 = interviewer observed that the child can do the activity**<br><br>ஆய்வாளர் கண்காணித்த போது குழந்தையால் நடவடிக்கையில் ஈடுபட முடிந்தது<br><br>**9 = not observed or the child refused**<br><br>கண்காணிக்க முடியவில்லை

**9 = அம்மா/கவனிப்பாளருக்குதெரியவில்லை / நினைவில்லை / கண்காணிக்கவில்லை**	அல்லது குழந்தை மறுத்து விட்டது

Start at the item appropriate for the child's age. If the child scores 0, go to the previous start point. If the child scores 1 or 2, continue to the next item. Stop after 4 consecutive scores of 0.

குழந்தையின்வயதுக்கேற்றகேள்வியிலிருந்துதுவங்கவும். ஒருவேளைகுழந்தை 0 மதிப்பெண்ணைஎடுத்தால், அதற்குமுந்தையதுவக்கஇடத்துக்குசெல்லவும். குழந்தை 1 அல்லது 2 மதிப்பெண்களைபெற்றால்அடுத்தகேள்விக்குசெல்லவும். குழந்தைதொடர்ந்து 0 மதிப்பெண்களை 4 முறைபெற்றால்அத்துடன்சோதனையைநிறுத்திக்கொள்ளவும்.

10-12 mo

START WITH THE BELOW QUESTION WHEN THE CHILD IS BETWEEN 0-7 MONTHS OLD.

		**Response | பதில்**
**5. GROSS MOTOR | மொத்ததிறன்**		
**Head Control | தலைக்கட்டுப்பாடு**		
**D5.1**	Can your child hold up his or her head for 5 seconds?<br>உங்கள்குழந்தையால் 5 விநாடிகளுக்குதனதுதலையைநேராகவைக்கமுடியுமா?	**D5.1.1|___| Code | குறியீடு**

How to administer 5.1 If you observe the child holding his or her head erect without support for more than 5 seconds, ask the mother how long the child has been able to do this. If you observe the mother supporting the child's head continuously, then ask the mother: *Are you ever able to leave the child's head unsupported? How long can your child support his/her own head?*

பிரிவு 5.1 -யைஎப்படிநடத்தவேண்டும்: எந்தவிதபிடிப்பும்இல்லாமல்குழந்தை 5 விநாடிகளுக்குமேலாகதனதுதலையைநேராகவைத்திருப்பதைநீங்கள்பார்த்தால், அக்குழந்தையால்எவ்வளவுநேரம்இதைசெய்யமுடியும்என்றுஅதன்அம்மாவிடம்கேட்கவும். ஒருவேளைகுழந்தையின்தலையைஅதன்அம்மாதொடர்ந்துதாங்கிப்பிடித்திருப்பதைநீங்கள்பார்த்தால், அந்தஅம்மாவைபார்த்துகேட்கவும்: நீங்கள்எப்போதாவதுகுழந்தையின்தலையைபிடிக்காமல்இருந்துள்ளீர்களா?

உங்கள்குழந்தையால்எவ்வளவுநேரம்தனதுதலையைபிடிமானம்இல்லாமல்வைத்திருக்க முடியும்?		
**D5.2**	Can your child hold his or her head up unsupported and turn his or her head to the left and to the right?<br><br>எந்தவிதபிடிப்பும்இல்லாமல்குழந்தைதனதுதலையைநேராகவைத்திருந்துஅதைவலதுபுறமும்இடதுபுறமும்திருப்ப முடியுமா?	**D5.2.1|___| Code | குறியீடு**
**How to administer 5.2** If you observe the child holding his or her head erect without support and turning his or her head to the right and to the left, ask the mother how long the child has been able to do this. If you observe the mother supporting the child's head, ask the mother: *Have you observed your child hold his head erect unsupported and turn his or her head to the left and to the right?*<br><br>**பிரிவு 5.2 -யைஎப்படிநடத்தவேண்டும்:**<br>எந்தவிதபிடிப்பும்இல்லாமல்குழந்தைதனதுதலையைநேராகவைத்திருந்துஅதைவலதுபுறமும்இடதுபுறமும்திருப்புவதைநீங்கள்பார்த்தால்,<br>அக்குழந்தையால்எவ்வளவுநேரம்இதைசெய்யமுடியும்என்றுஅதன்அம்மாவிடம்கேட்கவும்.<br>ஒருவேளைகுழந்தையின்தலையைஅதன்அம்மாதாங்கிப்பிடித்திருப்பதைநீங்கள்பார்த்தால், அந்தஅம்மாவைபார்த்துகேட்கவும்:<br>எந்தவிதபிடிப்பும்இல்லாமல்குழந்தைதனதுதலையைநேராகவைத்திருந்துஅதைவலதுபுறமும்இடதுபுறமும்திருப்புவதைநீங்கள்பார்த்துள்ளீர்களா?		
**START WITH THE BELOW QUESTION WHEN THE CHILD IS BETWEEN 8-9 MONTHS OLD and if response to F5.0==1**		
**Sitting | அமர்தல்**		
**D5.3**	Can your child sit supported?<br><br>உங்கள்குழந்தையால்பிடிமானத்துடன்உட்காரமுடியுமா?	**D5.3.1|___| Code | குறியீடு**
**D5.4**	Can your child sit alone on the floor?<br><br>உங்கள்குழந்தையால்தனியாகதரையில்உட்காரமுடியுமா ?	**D5.4.1|___| Code | குறியீடு**
**How to administer 5.3-5.4** Ask: *What happens when you leave the child to sit alone on the floor? How does he/she sit? **Additional probes:** Have you observed him/her sit upright by his/herself? Have you*		

observed him/her leaning on anything? Have you observed him/her sitting upright on your lap while leaning on you? Have you observed him/her sitting upright without leaning on his/her hands or on anything?<br><br>**பிரிவு 5.3-5.4 -யைஎப்படிநடத்தவேண்டும்:** குழந்தையைதரையில்தனியாகஅமர /உட்காரவைத்தால்என்னஆகிறது? குழந்தைஎப்படிஅமர்கிறான்/ள்? **கூடுதல்விசாரணை:**குழந்தைசுயமாகநிமிர்ந்துஉட்காருவதைநீங்கள்பார்த்துள்ளீர்களா? குழந்தைஎதன்மீதாவதுசாய்ந்துக்கொள்வதைபார்த்துள்ளீர்களா? உங்கள்மடியில்அமரும்போதுகுழந்தைஉங்கள்மீதுசாய்ந்துக்கொண்டுநேராகஅமர்வதைநீங்கள்பார்த்துள்ளீர்களா? குழந்தைஅமரும்போது/ உட்காரும்போதுதன்கைகளைதரையில்வைத்தோஅல்லதுவேறுஎதன்மீதாவதோசாய்ந்துக் கொள்ளாமல்நேராகஅமர்வதைபார்த்துள்ளீர்களா?		
**Standing | நிற்பது**		
**D5.5**	Can your child push down with feet on the floor when held?<br><br>நீங்கள்பிடித்திருக்கும்போதுஉங்கள்குழந்தையால்தனது பாதத்தைக்கொண்டுதரையில்அழுத்தமுடியுமா?	**D5.5.1|___| Code | குறியீடு**
**D5.6**	Can your child stand when held up?<br><br>நீங்கள்பிடித்திருக்கும்போதுஉங்கள்குழந்தையால்நிற்கமுடியுமா?	**D5.6.1|___| Code | குறியீடு**
**How to administer 5.5-5.6** Ask: *When you hold the child upright, what does he/she do? What does he/she do with his/her feet?* ***Additional probes:*** *Have you observed him/her try to place his/her feet flat on your lap or on the floor and push his/her feet into the floor? Have you observed him/her stand up while you're holding him/her?*<br><br>**பிரிவு 5.5-5.6 -யைஎப்படிநடத்தவேண்டும்:** நீங்கள்குழந்தையைநிற்கவைக்கும்போது, குழந்தைஎன்னசெய்கிறான்/ள்? தனதுபாதங்களைக்கொண்டுகுழந்தைஎன்னசெய்கிறான்/ள்? **கூடுதல்விசாரணை**: குழந்தைதனதுநேராகபாதத்தைதரையில்அல்லதுஉங்கள்மடியில்வைத்துஅழுத்துவதைநீங்கள்பார்த்துள்ளீர்களா? அவனை/ளைநீங்கள்பிடித்திருக்கும்போதுகுழந்தைஎழுந்துநிற்பதைநீங்கள்பார்த்துள்ளீர்களா		
**START WITH THE BELOW QUESTION WHEN THE CHILD IS BETWEEN 10-12 MONTHS OLD and if response to F5.0==1**		

**D5.7**	Can your child crawl 3 continuous movements without stomach on the ground?<br>உங்கள்குழந்தையால்வயிற்றுதரையில்படாமல்தொடர்ந்து 3 முறைதவழமுடியுமா?	**D5.7.1|___| Code | குறியீடு**
		**Response | பதில்**
**D5.8**	Can your child pull self into a standing position while holding on to object?<br>உங்கள்குழந்தையால்ஒருபொருளைப்பிடித்துக்கொண்டு தானாகஎழுந்துநிற்கமுடியுமா?	**D5.8.1|___| Code | குறியீடு**
**How to administer 5.8** Ask: *What happens when your child is sitting on the floor and wants to stand up? Have you observed him/her use a chair or another object to pull him/herself up to a standing position?*<br>**பிரிவு 5.8 -யைஎப்படிநடத்தவேண்டும்:**<br>உங்கள்குழந்தைதரையில்அமர்ந்திருந்துஎழுந்திருக்கநினைக்கும்போதுஎன்னஆகிறது? தான்பிடித்துக்கொண்டுஎழுந்துநிற்பதற்குகுழந்தைஒருநாற்காலியையோஅல்லதுஏதாவதுபொருளையோபயன்படுத்துவதைநீங்கள்பார்த்துள்ளீர்களா?		
**D5.9**	Can your child stand holding on to furniture or object 10 seconds?<br>உங்கள்குழந்தையால்ஒருநாற்காலிஅல்லதுபொருளைபிடித்துக்கொண்டு 10 விநாடிகள்நிற்கமுடியுமா?	**D5.9.1|___| Code | குறியீடு**
**How to administer 5.9 and 5.13** Ask: *What happens when you place your child in a standing position? What happens when you let go? **Additional probes:** Have you seen him/her standing up? Have you seen him/her standing while holding onto a chair or something else? Have you seen him/her standing up without holding onto anything?*<br>**பிரிவு 5.9 மற்றும் 5.13 -யைஎப்படிநடத்தவேண்டும்:**<br>நீங்கள்உங்கள்குழந்தையைநிற்கவைக்கும்போதுஎன்னஆகிறது?<br>குழந்தையைபோகவிடும்போதுஎன்னஆகிறது? **கூடுதல்விசாரணை**:<br>குழந்தைநிற்பதைநீங்கள்பார்த்துள்ளீர்களா?<br>குழந்தைஒருநாற்காலிஅல்லதுஏதாவதுபொருளைபிடித்துக்கொண்டுநிற்பதைநீங்கள்பார்த்துள்ளீர்களா?<br>குழந்தைஎதையும்பிடித்துக்கொள்ளாமல்தனியாகநிற்பதைநீங்கள்பார்த்துள்ளீர்களா?		

<table>
<tr><td colspan="3">START WITH THE BELOW QUESTION WHEN THE CHILD IS BETWEEN 13-19 MONTHS OLD and if response to F5.0==1</td></tr>
<tr><td>D5.10</td><td>Can your child walk when hands are held?

கைகளைபிடித்துள்ளபோதுஉங்கள்குழந்தையால்நடக்கமுடியுமா?</td><td>D5.10.1|___| Code | குறியீடு</td></tr>
<tr><td colspan="3">How to administer 5.10 and 5.14 Ask:Does your child move around? What happens when your child wants something that is not within reach? How does he/she move to get it? Additional probes: Have you observed your child moving on all fours? Have you observed your child moving forward on his/her feet when you are holding his/her hands? Have you observed your child walking on his/her own?

பிரிவு 5.10 மற்றும் 5.14 -யைஎப்படிநடத்தவேண்டும்:
உங்கள்குழந்தைநகர்ந்துசெல்கிறதா?
கைக்குஎட்டாததூரத்திலிருக்கும்ஒருபொருளைஎடுக்கவேண்டும்எனஉங்கள்குழந்தைநினைக்கும்போதுஎன்னஆகிறது? அதைஎடுப்பதற்குகுழந்தைஎப்படிநகர்ந்துசெல்கிறான்?
கூடுதல்விசாரணை:உங்கள்குழந்தைஅனைத்துவிதமானதரைகளிலும்நகர்ந்துசெல்வதைநீங்கள்பார்த்துள்ளீர்களா?
உங்கள்குழந்தையின்கைகளைநீங்கள்பிடித்திருக்கும்போதுகுழந்தைதனதுகால்களால்முன்நோக்கிநகர்வதைநீங்கள்பார்த்துள்ளீர்களா?
உங்கள்குழந்தைசுயமாகநடப்பதைநீங்கள்பார்த்துள்ளீர்களா?</td></tr>
<tr><td>D5.11</td><td>Can your child climb onto a low chair?

உங்கள்குழந்தையால்ஒருசின்ன / குட்டிநாற்காலியில்ஏறமுடியுமா?</td><td>D5.11.1|___| Code | குறியீடு</td></tr>
<tr><td colspan="3">How to administer 5.11 Ask: If your child wants to get into a low chair, what does he/she do? Have you observed him/her climbing up by him/herself? How does he/she do it?

பிரிவு 5.11 -யைஎப்படிநடத்தவேண்டும்: ஒருசின்ன / குட்டிநாற்காலியில்உட்காரநினைக்கும்போதுஉங்கள்குழந்தைஎன்னசெய்கிறது?
குழந்தைசுயமாகஅதன்மீதுஏறுவதைநீங்கள்பார்த்துள்ளீர்களா?
அதைஎப்படிகுழந்தைசெய்கிறான்/ள்?</td></tr>
<tr><td>D5.12</td><td>Can your child climb out of a low chair?

உங்கள்குழந்தையால்ஒருஉயரம்குறைந்தநாற்காலியிலிருந்துஇறங்கமுடியுமா?</td><td>D5.12.1|___| Code | குறியீடு</td></tr>
</table>

How to administer5.12 Ask: *If your child wants to get out of a low chair, what does he/she do? Have you observed him/her climbing out of the chair by him/herself?*

பிரிவு 5.12 -யைஎப்படிநடத்தவேண்டும்: ஒருசின்ன / குட்டிநாற்காலியில்இருந்துஇறங்கநினைக்கும்போதுஉங்கள்குழந்தைஎன்னசெய்கிறது? குழந்தைஅதிலிருந்துசுயமாகஇறங்குவதைநீங்கள்பார்த்துள்ளீர்களா?

**D5.13**	Can your child stand alone for 10 seconds?<br><br>உங்கள்குழந்தையால் 10 விநாடிகளுக்குதனியாகநிற்கமுடியுமா?	**D5.13.1|___| Code | குறியீடு**

How to administer 5.9 and 5.13 Ask: *What happens when you place your child in a standing position? What happens when you let go? **Additional probes:** Have you seen him/her standing up? Have you seen him/her standing while holding onto a chair or something else? Have you seen him/her standing up without holding onto anything?*

பிரிவு 5.9 மற்றும் 5.13 -யைஎப்படிநடத்தவேண்டும்:
நீங்கள்உங்கள்குழந்தையைநிற்கவைக்கும்போதுஎன்னஆகிறது? குழந்தையைபோகவிடும்போதுஎன்னஆகிறது? **கூடுதல்விசாரணை**: குழந்தைநிற்பதைநீங்கள்பார்த்துள்ளீர்களா? குழந்தைஒருநாற்காலிஅல்லதுஏதாவதுபொருளைபிடித்துக்கொண்டுநிற்பதைநீங்கள்பார்த்துள்ளீர்களா? குழந்தைஎதையும்பிடித்துக்கொள்ளாமல்தனியாகநிற்பதைநீங்கள்பார்த்துள்ளீர்களா?

START WITH THE BELOW QUESTION WHEN THE CHILD IS BETWEEN 20-23 MONTHS OLD and if response to F5.0==1

**D5.14**	Can your child walk alone 5 steps?<br><br>உங்கள்குழந்தையால்தனியாக 5 அடிகள்நடக்க முடியுமா?	**D5.14.1|___| Code | குறியீடு**

START WITH THE BELOW QUESTION WHEN THE CHILD IS BETWEEN 24-31 MONTHS OLD

How to administer 5.10 and 5.14 Ask:*Does your child move around? What happens when your child wants something that is not within reach? How does he/she move to get it? **Additional probes:** Have you observed your child moving on all fours? Have you observed your child moving forward on his/her feet when you are holding his/her hands? Have you observed your child walking on his/her own?*

பிரிவு 5.10 மற்றும் 5.14 -யைஎப்படிநடத்தவேண்டும்:
உங்கள்குழந்தைநகர்ந்துசெல்கிறதா?
கைக்குஎட்டாததூரத்திலிருக்கும்ஒருபொருளைஎடுக்கவேண்டும்எனஉங்கள்குழந்தைநினைக்கும்போதுஎன்னஆகிறது? அதைஎடுப்பதற்குகுழந்தைஎப்படிநகர்ந்துசெல்கிறான்?
கூடுதல்விசாரணை:உங்கள்குழந்தைஅனைத்துவிதமானதரைகளிலும்நகர்ந்துசெல்வதைநீங்கள்பார்த்துள்ளீர்களா?
உங்கள்குழந்தையின்கைகளைநீங்கள்பிடித்திருக்கும்போதுகுழந்தைதனதுகால்களால்முன்நோக்கிநகர்வதைநீங்கள்பார்த்துள்ளீர்களா?
உங்கள்குழந்தைசுயமாகநடப்பதைநீங்கள்பார்த்துள்ளீர்களா?

**D5.15**	Can your child go down one step while standing with support?<br><br>உங்கள்குழந்தையால்எதையாவதுபிடித்துக்கொண்டுநிற்கும்போதுஒரேஒருபடியைகீழேஇறங்கமுடியுமா?	**D5.15.1|___| Code |**<br>**குறியீடு**
**D5.16**	Can your child go down one step while standing without support?<br><br>உங்கள்குழந்தையால்எதையும்பிடித்துக்கொள்ளாமல்நிற்கும்போதுஒரேஒருபடியைகீழேஇறங்கமுடியுமா?	**D5.16.1|___| Code |**<br>**குறியீடு**

How to administer 5.15-5.16 Ask the mother: *What happens when your child tries to walk out of a house when there is a step? Have you seen him/her go down the step? How does he/she do it?* ***Additional probes:*** *Have you seen him/her sit down on his/her bottom or on his/her knees to go down the step? Does he/she hold onto something? Have you seen him/her go down the step without touching anything with his/her hands?*

பிரிவு 5.15-5.16 -யைஎப்படிநடத்தவேண்டும்: அம்மாவைபார்த்துகேட்கவும்:
உங்கள்குழந்தைவீட்டைவிட்டுவெளியேவரநினைக்கும்போதுஅங்குபடிகள்இருந்தால்என்னஆகிறது?
குழந்தைஅந்தபடிகளில்இறங்கிசெல்லமுயற்சிப்பதைநீங்கள்பார்த்துள்ளீர்களா?
குழந்தைஅதைஎப்படிசெய்கிறான்/ள்?
கூடுதல்விசாரணை:அந்தபடிகளில்இறங்கிசெல்லவதற்குஉதவியாககுழந்தைஉட்காருவதையோஅல்லதுமுழங்காலில்நிற்பதையோநீங்கள்பார்த்துள்ளீர்களா?
குழந்தைஎதையாவதுபிடித்துக்கொள்கிறானா/ளா?
குழந்தைதனதுகைகளால்எதையும்பிடித்துக்கொள்ளாமல்படிகளில்இறங்கிசெல்வதைபார்த்துள்ளீர்களா?

START WITH THE BELOW QUESTION WHEN THE CHILD IS BETWEEN 32-37 MONTHS OLD (and if resposne to F5.0 is ==1

<table>
<tr><td>D5.17</td><td>Can your child run?

உங்கள்குழந்தையால்ஓடமுடியுமா?</td><td>D5.17.1|___| Code | குறியீடு</td></tr>
<tr><td colspan="3">How to administer5.17Ask:Have you seen your child run?

பிரிவு 5.17 -யைஎப்படிநடத்தவேண்டும்: உங்கள்குழந்தைஓடுவதைநீங்கள்பார்த்துள்ளீர்களா?

குழந்தையிடம்கூறவும்: நாமஇப்போஅங்கேஓடிதிரும்பவரப்போறோம் (குழந்தைக்குசெய்துகாண்பிக்கவும்). இப்போநீஓடுபார்க்கலாம்.</td></tr>
<tr><td>D5.18</td><td>Can your child jump? (both feet leaving the ground at the same time)

உங்கள்குழந்தையால்குதிக்கமுடியுமா? (இரண்டுகால்களும்ஒரேசமயத்தில்தரையிலிருந்துமேலெழும்பவேண்டும்)</td><td>D5.18.1|___| Code | குறியீடு</td></tr>
<tr><td colspan="3">How to administer5.18 Ask:Have you observed your child jump with both feet leaving the ground at the same time? Have you observed your child do this without holding on to anything?

பிரிவு 5.18 -யைஎப்படிநடத்தவேண்டும்: உங்கள்குழந்தைதனதுஇரண்டுகால்களையும்ஒரேசமயத்தில்தூக்கியபடிதரையிலிருந்து மேலெழும்பிகுதிப்பதைநீங்கள்பார்த்துள்ளீர்களா? உங்கள்குழந்தைஎதையும்பிடித்துக்கொள்ளாமல்இதைசெய்வதைநீங்கள்பார்த்துள்ளீர்களா?

குழந்தையைபார்த்துகூறவும்: நாமஇப்போஇப்படிஉயரமாகுதிக்கப்போறோம் (குழநந்தைக்குசெய்துகாண்பிக்கவும்). இப்போநீகுதிபார்க்கலாம்.</td></tr>
<tr><td>D5.19</td><td>Can your child kick a ball while standing?

உங்கள்குழந்தையால்நிற்கும்போதுஒருபந்தைஉதைக்கமுடியுமா?</td><td>D5.19.1|___| Code | குறியீடு</td></tr>
<tr><td colspan="3">How to administer 5.19 Ask:Have you observed your child try to kick a ball? What happens? Have you seen his/her foot make solid contact with the ball? Have you observed him/her kick the ball without falling?</td></tr>
</table>

32-m

**பிரிவு 5.19 -யைஎப்படிநடத்தவேண்டும்:** உங்கள்குழந்தைஒருபந்தைஉதைக்கமுயற்சிப்பதைநீங்கள்பார்த்துள்ளீர்களா? அந்தபந்துடன்அவனது/எதுபாதம்நல்லதிடமானதொடர்புஏற்படுத்துவதைநீங்கள்பார்த்துள்ளீர்களா? குழந்தைகீழேவிழாமல்பந்தைஉதைப்பதைநீங்கள்பார்த்துள்ளீர்களா?<br><br>குழந்தையைபார்த்துகூறவும்: நாமஇப்போஇந்தபந்தைஇப்படிஉதைக்கப்போறோம் (குழந்தைக்குசெய்துகாண்பிக்கவும்). இப்போநீசெய்பார்க்கலாம்.		
**D5.20**	Can your child throw a ball?<br><br>உங்கள்குழந்தையால்ஒருபந்தைதூக்கியெறியமுடியுமா?	**D5.20.1|___| Code | குறியீடு**
**How to administer5.20**Ask: *Have you observed your child try to throw a ball? What happens? Does it go in the right direction?*<br><br>**பிரிவு 5.20 -யைஎப்படிநடத்தவேண்டும்:** உங்கள்குழந்தைஒருபந்தைதூக்கியெறியமுயற்சிப்பதைநீங்கள்பார்த்துள்ளீர்களா? என்னஆகிறது? அந்தபந்துசரியானதிசையில்தான்போகிறதா?<br><br>குழந்தையைபார்த்துகூறவும்: நாமஇப்போஇந்தபந்தைஇப்படிதூக்கியெறியபோறோம் (அம்மாவிடம்பந்தைதூக்கியெறிந்துகாண்பிக்கவும்). இப்போநீசெய்பார்க்கலாம்.		

5 வயதுக்குட்பட்ட குழந்தைகள் மொழி, அடிப்படை இயக்கம்,சிறப்பு இயக்கம் ஆகியவற்றை அளவீடு செய்வதற்குDMC-III 86வினாபட்டியலில் வகையான கேள்விக்கள் உள்ளனமேற்பான்மையான கேள்விகள் பெற்றோர்கள் தரும் பதில்களின் அடிப்படையில் உள்ளது 7.21 முதல் 7.24 வரை மற்றும் 7.33 வரை உள்ள கேள்விகளை நேர்காணல் நடத்துபவர் கண்டிப்பாக குழந்தையின் தாயார் அல்லது குழந்தையின் முதன்மை பாதுகாவலரிடம்கேட்கவேண்டும்

**START WITH THE BELOW QUESTION WHEN THE CHILD IS GREATER THAN 37 MONTHS OLD and if response to F5.0==1**		
**D5.21**	Can your child walk backwards 5 steps?<br><br>உங்கள்குழந்தையால் 5 அடிகள்பின்நோக்கிநடக்கமுடியுமா?	**D5.21.1|___| Code | குறியீடு**

How to administer5.21 Ask:*Have you observed your child try to walk backwards? What happens? Have you observed him/her take 5 steps backwards without losing balance?*

பிரிவு 5.21 -யைஎப்படிநடத்தவேண்டும்: உங்கள்குழந்தைபின்நோக்கிநடக்கமுயற்சிப்பதைநீங்கள்பார்த்துள்ளீர்களா? என்னஆகிறது? குழந்தைதனதுசமநிலையையிழக்காமல் 5 அடிகளைஎடுத்துவைப்பதைநீங்கள்பார்த்துள்ளீர்களா?

குழந்தையைபார்த்துகூறவும்: நாமஇப்போஇதைப்போலபின்னாடிநடக்கப்போறோம் (குழந்தைக்குசெய்துகாண்பிக்கவும்). இப்போநீசெய்பார்க்கலாம்.

**D5.22**	Can your childstand on one leg for at least 1 second?<br>உங்கள்குழந்தையால்குறைந்தது 1 விநாடியாவதுஒற்றைக்காலில்நிற்கமுடியுமா?	**D5.22.1|___| Code | குறியீடு**
**D5.23**	Can your child stand on one leg for at least 10 seconds?<br>உங்கள்குழந்தையால்குறைந்தது 10 விநாடிகளாவதுஒற்றைக்காலில்நிற்கமுடியுமா?	**D5.23.1|___| Code | குறியீடு**

How to administer5.22-5.23 Ask: *Have you observed your child try to stand on one leg? What happens? Have you observed him/her stand on one leg for at least one second? For at least ten seconds?*

பிரிவு 5.22-5.23 -யைஎப்படிநடத்தவேண்டும்: உங்கள்குழந்தைஒற்றைக்காலில்நிற்கமுயற்சிப்பதைநீங்கள்பார்த்துள்ளீர்களா? என்னஆகிறது? குழந்தைகுறைந்ததுஒருவிநாடியாவதுஒற்றைக்காலில்நிற்பதைநீங்கள்பார்த்துள்ளீர்களா? குறைந்தது 10 விநாடிகள்?

குழந்தையைபார்த்துகூறவும்: நாமஇப்போஇதைப்போலஒற்றைக்காலில்நிற்கப்போறோம் (குழந்தைக்குசெய்துகாண்பிக்கவும்). இப்போநீசெய்பார்க்கலாம்.

	**Response | பதில்**

**D5.24**	Can your child hop forward on one foot four times in a row?<br><br>உங்கள்குழந்தையால்தொடர்ந்துநான்குமுறைஒற்றைக்காலில்குதிக்க / தாவிதாவி (நொண்டியாட/பாண்டியாட) முடியுமா?	**D5.24.1|___| Code | குறியீடு**

How to administer5.24*Say to the child: we are going to hop on one foot like this (demonstrate to child). Now you try.*

பிரிவு 5.24 -யைஎப்படிநடத்தவேண்டும்: குழந்தையிடம்கூறவும்: நாமஇப்போஇதைப்போலஒற்றைக்காலில்தத்திக்குதிக்கப்போறோம் (நொண்டியாடுதல்/பாண்டியாடுதல்) (குழந்தைக்குசெய்துகாண்பிக்கவும்). இப்போநீசெய்பார்க்கலாம்.

**D5.25**	Can your child throw a ball into the air and then catch it?<br><br>உங்கள்குழந்தையால்ஒருபந்தைமேலேதூக்கிப்போட்டுபிடிக்கமுடியுமா?	**D5.25.1|___| Code | குறியீடு**

How to administer5.25*Say to the child: we are going to throw and catch like this (demonstrate to child). Now you try.*

பிரிவு 5.25 -யைஎப்படிநடத்தவேண்டும்: குழந்தையைபார்த்துகூறவும்: நாமஇப்போஇதைப்போலபந்தைதூக்கிப்போட்டுபிடிக்கப்போறோம் (குழந்தைக்குசெய்துகாண்பிக்கவும்). இப்போநீசெய்பார்க்கலாம்.

**D5.26**	Can your child walk 3 steps putting one foot in front of the other with heel touching toe?<br><br>உங்கள்குழந்தையால்அடிமேல்அடிவைத்து (அப்படிவைக்கும்போதுமுன்னங்காலின்குதிக்கால்பின்னங்காலின்விரல்களைதொடவேண்டும்) 3 அடிகள்நடக்கமுடியுமா?	**D5.26.1|___| Code | குறியீடு**

How to administer5.26*Say to the child: we are going to walk one foot in front of the other like this (demonstrate). Now you try.*

பிரிவு 5.26 -யைஎப்படிநடத்தவேண்டும்: குழந்தையைபார்த்துகூறவும்: நாமஇப்போஇதைப்போலஅடிமேலேஅடிவைச்சுநடக்கப்போறோம் (குழந்தைக்குசெய்துகாண்பிக்கவும்). இப்போநீசெய்பார்க்கலாம்.

TOTAL GROSS MOTOR SCORE = SUM OF RESPONSE ITEMS 5.1.1 - 5.26.1<br><br>ஒட்டுமொத்ததிறன்மதிப்பெண் = பதில்களின்கூட்டுத்தொகை (5.1.1 - 5.26.1)		|___|___|
**6. FINE MOTOR | அறிவுசார்திறன்**		
**Watching and reaching | பார்ப்பதும்மற்றும்அடைவதும்**		
**START WITH THE BELOW QUESTION IF THE CHILD IS LESS THAN 5 MONTHS OF AGE and if response to F5.0==1**		
**D6.1**	Can your child watch a moving item in front of his or her face?<br><br>உங்கள்குழந்தையால்தனதுமுகத்துக்குமுன்பாகநகரும்ஒருபொருளைபார்க்கமுடியுமா?	**D6.1.1|___| Code | குறியீடு**
**D6.2**	Can your child reach out for objects even if child doesn't manage to grasp it?<br><br>உங்கள்குழந்தையால்தன்னால்எடுக்கமுடியாதபொருளை கையைநீட்டிஎடுக்க/ பிடிக்கமுயற்சிக்கமுடியுமா?	**D6.2.1|___| Code | குறியீடு**
**D6.3**	Can your child reach out and grasp objects?<br><br>உங்கள்குழந்தையால்பொருட்களைஎட்டிபிடிக்கமுடியுமா?	**D6.3.1|___| Code | குறியீடு**
**How to administer6.1-6.3** Ask: *When you hold a cloth or a toy in front of the child's face what does he/she do? **Additional probes:** Have you seen your child watch the object and follow it with his/her eyes? Have you seen your child reach out his/her arm/hand to try to get the object? Have you seen the child manage to take the object?*<br><br>**பிரிவு 6.1-6.3 -யைஎப்படிநடத்தவேண்டும்:** குழந்தையின்முகத்துக்குமுன்பாகஒருதுணிஅல்லதுஒருபொம்மையைவைக்கும்போதுகுழந்தைஎன்னசெய்கிறான்/ள்?<br>**கூடுதல்விசாரணை:**உங்கள்குழந்தைஅந்தபொருளைபார்த்துதனதுகண்களால்அதை தொடர்ந்துகண்காணிப்பதைநீங்கள்பார்த்துள்ளீர்களா? அந்தபொருளைபிடிப்பதற்குகுழந்தைதனதுகை/கரங்களைநீட்டுவதைநீங்கள்பார்த்துள்ளீர்களா? அந்தபொருளைஎடுப்பதற்காககுழந்தைமுயற்சிப்பதுநீங்கள்பார்த்துள்ளீர்களா?		
**Picking things up | பொருட்களைஎடுத்தல்**		

<table>
<tr><td colspan="3">START WITH THE BELOW QUESTION IF THE CHILD IS 6-9 MONTHS OF AGE and if response to F5.0==1</td></tr>
<tr><td>D6.4</td><td>Can your child pick up small objects in any way i.e. smaller than the child's hand?

உங்கள்குழந்தையால்எப்படியாவதுசிறிய / சின்னபொருட்களைகையில்எடுக்கமுடியுமா?</td><td>D6.4.1|___| Code | குறியீடு</td></tr>
<tr><td>D6.5</td><td>Can your child pick up small objects using one hand rather than two?

உங்கள்குழந்தையால்சிறிய/சின்னபொருட்களைஒரேகை யில்எடுக்கமுடியுமா?
(இரண்டுகைகளைபயன்படுத்தாமல்)</td><td>D6.5.1|___| Code | குறியீடு</td></tr>
<tr><td colspan="3">How to administer6.4-6.5 Ask: If there is a small toy or an object on the floor or table in front of the child, what does he/she do? Additional probes: Have you seen him/her pick it up? How? Does he/she use one hand or two?

பிரிவு 6.4-6.5 -யைஎப்படிநடத்தவேண்டும்:
உங்கள்குழந்தைக்குமுன்பாகதரையில்ஒருசிறயபொம்மைஅல்லதுஒருபொருள்அல்லதுஒ ருமேஜைஇருந்தால்குழந்தைஎன்னசெய்வான்/ள்?
கூடுதல்விசாரணை:குழந்தைஅதைஎடுப்பதைபார்த்துள்ளீர்களா? எப்படி? குழந்தைதனதுஇரண்டுகைகளையும்பயன்படுத்துகிறனா/ளாஅல்லதுஒருகையைமட்டுமா ?</td></tr>
<tr><td colspan="3">START WITH THE BELOW QUESTION IF THE CHILD IS 10-17 MONTHS OF AGE and if response to F5.0==1</td></tr>
<tr><td>D6.6</td><td>Can your child pick peas or lentils with thumb and forefinger?

உங்கள்குழந்தையால்ஆள்காட்டிவிரல்மற்றும்கட்டைவிர லைபயன்படுத்திதானியங்கள்அல்லதுமணிகள்எடுக்கமு டியுமா?</td><td>D6.6.1|___| Code | குறியீடு</td></tr>
<tr><td colspan="3">How to administer6.6Ask:If there are small grains or beads on the ground in front of the child, how does he/she pick them up? Additional probes:Have you observed your child pick up small grains using only his/her thumb and forefinger?</td></tr>
</table>

**பிரிவு 6.6 -யைஎப்படிநடத்தவேண்டும்:** குழந்தைக்குமுன்பாகதரையில்சிறியதானியங்களோஅல்லதுமணிகள்இருந்தால், அதைகுழந்தைஎப்படிஎடுப்பான்/ள்? **கூடுதல்விசாரணை**: உங்கள்குழந்தைதனதுஆள்காட்டிவிரலையும்கட்டைவிரலையும்பயன்படுத்திசிறியதானியங்களையோஅல்லதுமணிகள்எடுப்பதைநீங்கள்பார்த்துள்ளீர்களா?		
**D6.7**	Can your child open a door that requires pushing?<br><br>உங்கள்குழந்தையால்ஒருகதவைதள்ளிதிறக்கமுடியுமா?	**D6.7.1|___| Code | குறியீடு**
**How to administer 6.7** Ask: *What does your child do when he/she wants to go through a closed door?* ***Additional probes:*** *Have you observed him/her push the door open on his/her own without any help?*<br><br>**பிரிவு 6.6 -யைஎப்படிநடத்தவேண்டும்:** ஒருமூடியுள்ளகதவுவழியாகபோகவேண்டும்என்றுநினைக்கும்போதுஉங்கள்குழந்தைஎன்னசெய்யும்? **கூடுதல்விசாரணை:** குழந்தைஎந்தவொருதுணையும்இல்லாமல்கதவைசுயமாகதிறப்பதைபார்த்துள்ளீர்களா?		
**START WITH THE BELOW QUESTION IF THE CHILD IS 18-42 MONTHS OF AGE and if response to F5.0==1**		
**Writing | எழுதுதல்**		
**D6.8**	Can your child hold a pen in any way with the intent to write?<br><br>உங்கள்குழந்தையால்எழுதவேண்டும்என்கிறநோக்கத்துடன்ஒருபேனாவைஎந்தவொருமுறையிலாவதுகையில்பிடிக்கமுடியுமா?	**D6.8.1|___| Code | குறியீடு**
**D6.9**	Can your child hold a pen between finger and thumb (like an adult)?<br><br>உங்கள்குழந்தையால்ஒருபேனாவைஆள்காட்டிவிரல்மற்றும்கட்டைவிரலுக்குஇடையேவைத்துபிடிக்கமுடியுமா (பெரியவர்களைபோல)?	**D6.9.1|___| Code | குறியீடு**
**D6.10**	Can your child scribble with a pen?<br><br>உங்கள்குழந்தையால்பேனாவைவைத்துகிறுக்கமுடியுமா?	**D6.10.1|___| Code | குறியீடு**

**D6.11**	Can your child scribble with a pen without going off the page?<br><br>உங்கள்குழந்தையால்பேனாவைவைத்துபெபர் / காகிதத்திற்குவெளியேபோகதபடிகிறுக்கமுடியுமா?	**D6.11.1|___| Code | குறியீடு**

How to administer 6.8-6.11 Ask: *Have you observed your child holding a pen or pencil? What does your child do when you give him/her a pen? How does he/she hold it? What does he/she do with it?* ***Additional probes:*** *Have you observed him/her move it along the floor or a table as if writing or drawing? Have you observed your child scribble on paper?*

பிரிவு 6.8-6.11 -யைஎப்படிநடத்தவேண்டும்: உங்கள்குழந்தைகையில்பேனாஅல்லதுபென்சில்வைத்திருப்பதைநீங்கள்பார்த்துள்ளீர்களா? உங்கள்குழந்தையிடம்நீங்கள்பேனாஅல்லதுபென்சில்கொடுக்கும்போதுகுழந்தைஎன்ன செய்வான்/ள்? குழந்தைஅதைகையில்எப்படிபிடிப்பான்/ள்? அதைக்கொண்டுகுழந்தைஎன்னசெய்வான்/ள்? **கூடுதல்விசாரணை:**அதைக்கொண்டுதரையிலோஅல்லதுமேஜையின்மீதோகுழந்தைஎழுதுவதுஅல்லதுவரைவதைப்போல்கிறுக்குகிறானா? உங்கள்குழந்தைபெபர் /காகிதத்தில்கிறுக்குவதைநீங்கள்பார்த்துள்ளீர்களா?

குழந்தையைபார்த்துகூறவும்: நாமஇப்போஏதாவதுகிறுக்கலாமா/வரையலாமா? என்னைப்பார் (கிறுக்குவதைசெய்துகாண்பிக்கவும்). இப்போநீசெய்பார்க்கலாம்.

START WITH THE BELOW QUESTION IF THE CHILD IS GREATER THAN 42 MONTHS OF AGE and if response to F5.0==1

**D6.12**	Can your child draw a straight line?<br><br>உங்கள்குழந்தையால்ஒருநேர்கோட்டைவரையமுடியுமா?	**D6.12.1|___| Code | குறியீடு**
**D6.13**	Can your child draw a circle?<br><br>உங்கள்குழந்தையால்ஒருவட்டத்தைவரையமுடியுமா?	**D6.13.1|___| Code | குறியீடு**
**D6.14**	Can your child draw a triangle?<br><br>உங்கள்குழந்தையால்ஒருமுக்கோணத்தைவரையமுடியுமா?	**D6.14.1|___| Code | குறியீடு**
		**Response | பதில்**

**D6.15**	Child draws a picture of a person with 1 or more body parts<br><br>குழந்தை 1 அல்லதுஅதற்குமேற்பட்டஉடல்பாகங்களுடன்படத்தைவரைகிறது	**D6.15.1|___| Code | குறியீடு**
**D6.16**	Child draws a picture of a person with 2 or more body parts<br><br>குழந்தை 2 அல்லதுஅதற்குமேற்பட்டஉடல்பாகங்களுடன்படத்தைவரைகிறது	**D6.16.1|___| Code | குறியீடு**
**D6.17**	Child draws a picture of a person with 4 or more body parts<br><br>குழந்தை 4 அல்லதுஅதற்குமேற்பட்டஉடல்பாகங்களுடன்படத்தைவரைகிறது	**D6.17.1|___| Code | குறியீடு**

How to administer 6.15-6.17*Do not demonstrate.*

Child should draw a picture of a person with any of these parts: head, legs, arms, eyes, body, nose, ears, hands

பிரிவு 6.15-6.17 -யைஎப்படிநடத்தவேண்டும்: இதைசெய்துகாண்பிக்கவேண்டாம். குழந்தையைபார்த்துகூறவும்: "நாமஇப்போஒருபடத்தைவரையலாமா, எ.கா. அம்மாஇல்லஅப்பாவோடபடம், சரியா? நீசெய்பார்க்கலாம். ஒருவேளைகுழந்தைக்குபுரியவில்லைஎன்றால், இதைதிரும்பகூறவும். அதற்குபிறகும்குழந்தைபுரிந்துக்கொள்ளவில்லைஎன்றால், குழந்தையைபார்த்துகூறவும்: சரி, இப்போஅம்மாவோடதலையைவரைவோமா. இதைநீங்கள்வரையவேண்டும். குழந்தைபின்வரும்உடல்பாகங்களுடன்ஒருவரதுபடத்தைவரையவேண்டும்: தலை, கால்கள், கரங்கள், கண்கள், உடல், மூக்கு, காதுகள், கைகள்."

**D6.18**	Child writes 1 or more letters<br><br>குழந்தை 1 அல்லதுஅதற்குமேற்பட்டஎழுத்துக்களைஎழுதுகிறது	**D6.18.1|___| Code | குறியீடு**
**D6.19**	Child writes 2 or more letters<br><br>குழந்தை 2 அல்லதுஅதற்குமேற்பட்டஎழுத்துக்களைஎழுதுகிறது	**D6.19.1|___| Code | குறியீடு**

**How to administer 6.18-6.19** *Do not demonstrate.* **பிரிவு 6.18-6.19 -யைஎப்படிநடத்தவேண்டும்:** இதைசெய்துகாண்பிக்கவேண்டாம். குழந்தையைபார்த்துகூறவும்: “நாமஇப்போசிலஎழுத்துக்களைஎழுதுவோமா. உன்னாலஎன்னஎழுதமுடியும், எழுதிகாட்டுபார்க்கலாம்! உன்னால A எழுதமுடியுமா? T எழுதிகாட்டு? O எழுதிகாட்டு?” குழந்தைபதிலேதும்கூறவில்லைஎன்றால், அக்குழந்தையால்என்னஎழுத்தைஎழுதமுடியும்என்பதைஅம்மாவிடம்கேட்டுஅதைஎழுதும் படிகுழந்தையைபார்த்துகூறவும்.		
**D6.20**	Child writes 1 or more numbers<br><br>குழந்தை 1 அல்லதுஅதற்குமேற்பட்டநம்பேர் / எண்களைஎழுதுகிறது	**D6.20.1|___| Code | குறியீடு**
**D6.21**	Child writes 2 or more numbers<br><br>குழந்தை 2 அல்லதுஅதற்குமேற்பட்டநம்பேர் /எண்களைஎழுதுகிறது	**D6.21.1|___| Code | குறியீடு**
**D6.22**	Child writes 3 or more numbers<br><br>குழந்தை 3 அல்லதுஅதற்குமேற்பட்டநம்பேர் /எண்களைஎழுதுகிறது	**D6.22.1|___| Code | குறியீடு**
**How to administer 6.20-6.22** *Do not demonstrate.* **பிரிவு 6.20-6.22 -யைஎப்படிநடத்தவேண்டும்:** இதைசெய்துகாண்பிக்கவேண்டாம். குழந்தையிடம்கூறவும்: “நாமஇப்போஏதாவதுநம்பரஎழுதலாமா. 1 எழுதுபார்க்கலாம்? 2 எழுதிகாட்டு? 3, 4 என்றுசொல்லிக்கொண்டேபோகவும்.		
**D6.23**	Child writes 1-2 or more lettersin order after “A” (B or B, C)<br><br>குழந்தை “அ” -வுக்குஅடுத்து (ஆஅல்லதுஆ, இ) 1-2 அல்லதுஅதற்குமேற்பட்டஎழுத்துக்களைவரிசையாகஎழுது கிறது	**D6.23.1|___| Code | குறியீடு**
**D6.24**	Child writes 3 or more letters in order after “A” (B, C, D, and so on)<br><br>குழந்தை “அ” -வுக்குஅடுத்து (ஆ, இ, ஈமற்றும்தொடர்ந்து) 3	**D6.24.1|___| Code | குறியீடு**

<table>
<tr><td></td><td>அல்லதுஅதற்குமேற்பட்டஎழுத்துக்களைவரிசையாகஎழுது கிறது</td><td></td></tr>
<tr><td colspan="3">How to administer 6.23-6.24 Do not demonstrate. பிரிவு 6.23-6.24 -யைஎப்படிநடத்தவேண்டும்: இதைசெய்துகாண்பிக்கவேண்டாம். குழந்தையைபார்த்துகூறவும்: “நாமஇப்போஇங்கிலீஷ்எழுத்துக்களைவரிசையாஎழுதலாமா. A -விலிருந்துதுவங்கவும். அடுத்துஎன்னவரும்? அதைஎழுது!” தொடர்ந்துகுழந்தையைகேட்கவும், “அடுத்துஎன்னவரும்?”
ஆனாலும்அடுத்துஎன்னஎழுத்துவரும்என்பதைநீங்கள்குழந்தையிடம்கூறவேண்டாம்.</td></tr>
<tr><td>D6.25</td><td>Child writes 1 or more words

குழந்தை 1 அல்லதுஅதற்குமேற்பட்டவார்த்தைகளைஎழுதுகிறது</td><td>D6.25.1|___| Code | குறியீடு</td></tr>
<tr><td>D6.26</td><td>Child writes 2 or more words

குழந்தை 2 அல்லதுஅதற்குமேற்பட்டவார்த்தைகளைஎழுதுகிறது</td><td>D6.26.1|___| Code | குறியீடு</td></tr>
<tr><td>D6.27</td><td>Child writes 4 or more words

குழந்தை 4 அல்லதுஅதற்குமேற்பட்டவார்த்தைகளைஎழுதுகிறது</td><td>D6.27.1|___| Code | குறியீடு</td></tr>
<tr><td colspan="3">How to administer 6.25-6.27Do not demonstrate.

பிரிவு 6.25-6.27 -யைஎப்படிநடத்தவேண்டும்: இதைசெய்துகாண்பிக்கவேண்டாம். குழந்தையிடம்கூறவும்: “நாமஇப்போசிலவார்த்தைகளைஎழுதலாமா. “apple” என்கிறவார்த்தையைஎழுதுபார்க்கலாம்? “ball” எழுதிகாட்டு? “ball” -யைஎழுதிக்காட்டவும். தொடர்ந்து “tree” “monkey” மற்றும் “elephant” வார்த்தைகளைகூறவும். நீங்கள்படித்துக்காண்பிக்கும்வார்த்தைகளைகுழந்தைபார்க்கவில்லைஎன்பதைஉறுதிசெய்துக்கொண்டுஒருவார்த்தையைஎழுதுவதற்குகுழந்தைக்குபோதுமானநேரம்கொடுக்கவும்.</td></tr>
<tr><td colspan="2">TOTAL FINE MOTOR SCORE = SUM OF RESPONSE ITEMS 6.1.1 - 6.27.1

மொத்தஅறிவுசார்திறன்மதிப்பெண் = பதில்களின்கூட்டுத்தொகை (6.1.1 - 6.27.1)</td><td>|___|___|</td></tr>
<tr><td colspan="3">7. LANGUAGE | மொழி</td></tr>
</table>

**Pre-speech language | குழந்தைமொழி**		
**START WITH THE BELOW QUESTION IF THE CHILD IS LESS THAN 8 MONTHS OF AGE and if response to F5.0==1**		
**D7.1**	Is your child startled by loud noises?<br><br>உங்கள்குழந்தைஉரத்த / அதிகசப்தம்கேட்டால் பயப்படுகிறதா?	**D7.1.1|___| Code | குறியீடு**
**How to administer7.1** Ask: *What does your child do when there is a loud sound like a shout or a loud knock?*<br><br>**பிரிவு 7.1 -யைஎப்படிநடத்தவேண்டும்:** ஒருகூச்சல்அல்லதுஉரத்த/ அதிகசப்தம்போன்றஅதிகசத்தம்கேட்கும்போதுஉங்கள்குழந்தைஎன்னசெய்யும்?		
**D7.2**	Can your child repeat vowels in strings (ex : aa aaaa)?<br><br>உங்கள்குழந்தையால்உயிரெழுத்துக்களைகோர்வையாக திரும்பகூறமுடியுமா (உதா: அஅஅ)?	**D7.2.1|___| Code | குறியீடு**
**START WITH THE BELOW QUESTION IF THE CHILD IS 9-12 MONTHS OF AGE and if response to F5.0==1**		
**D7.3**	Can your child repeat syllables in strings (ex : ma mama)?<br><br>உங்கள்குழந்தையால்ஒருஅசைவெழுத்துக்களை/ வார்த்தைகேட்டப்பிற்குதிரும்பதானகூறமுடியுமா (உதா: மாமாமா)?	**D7.3.1|___| Code | குறியீடு**
**How to administer7.2-7.3**: Observe the child as you have opportunity during the interview. Take note of the child's vocalizations. If you hear the child making vowel sounds and/or syllable sounds, ask the mother how long the child has been making those sounds. If you do not hear the child making any sounds, ask the mother: *Does your child make any sounds? What sounds does he/she make? **Additional probes:** If you talk to her and say "aa aaaa" what does she say?Does she say it back to you? If you talk to her and say "bababa" what does she say? Does she say it back to you?*<br><br>**பிரிவு 7.2-7.3 -யைஎப்படிநடத்தவேண்டும்:**<br>நேர்காணலின்போதுஉங்களுக்குவாய்ப்பிருக்கும்காரணத்தால்குழந்தையைகண்காணிக் கவும். குழந்தைஎழுப்பும்ஒலிகளைகுறிப்பெடுத்துக்கொள்ளவும்.<br>ஒருவேளைகுழந்தைஉயிரெழுத்துஒலிகளையோமற்றும்/அல்லதுஅசைவெழுத்துஒலிகளை யோஏற்படுத்துவதைநீங்கள்கவனித்தால்,		

அந்தஒலிகளைகுழந்தைஎவ்வளவுநாட்களாகஎழுப்புகிறதுஎன்றுஅம்மாவிடம்கேட்கவும். ஒருவேளைகுழந்தைஒலிஎதுவும்எழுப்பவில்லைஎனில், அம்மாவிடம்கேட்கவும்: உங்கள்குழந்தைஏதாவதுஒலிஎழுப்புகிறனா/ளா? குழந்தைஎன்னஒலியைஎழுப்புகிறான்/ள்? **கூடுதல்விசாரணை**:நீங்கள்குழந்தையிடம்பேசி "aa aaa" என்றுகூறினால்பதிலுக்குகுழந்தைஎன்னசொல்கிறது? அதைதிரும்பகூறுகிறதா? நீங்கள்குழந்தையிடம்பேசி "bababa" என்றுகூறினால்பதிலுக்குகுழந்தைஎன்னசொல்கிறது? அதைதிரும்பகூறுகிறதா?		
		**Response | பதில்**
**D7.4**	Can your child understand the gesture reaching out to ask for something?<br><br>உங்கள்கைகள்செய்கைமூலம்காண்பித்தால்குழந்தைக்குபுரிந்துக்கொள்ளமுடியுமா?	**D7.4.1|___| Code | குறியீடு**
**How to administer 7.4** Ask: *If you stretch out your arm to ask your child for something, does he/she give you something? Even if it's not what you asked for, does the child understand the gesture reaching out to ask for something?*<br><br>**பிரிவு 7.4 -யைஎப்படிநடத்தவேண்டும்:**<br>நீங்கள்உங்களதுகையைநீட்டிஎதையாவதுகுழந்தையிடமிருந்துகேட்டால், குழந்தைஉங்களுக்குஎதையாவதுதருகிறானா/ளா? ஒருவேளைநீங்கள்கேட்பதுகுழந்தையிடம்இல்லைஎன்றாலும், அதனிடம்எதையோகேட்கிறீர்கள்எனும்சைகையைகுழந்தைபுரிந்துக்கொள்கிறதா?		
**START WITH THE BELOW QUESTION IF THE CHILD IS 13-21 MONTHS OF AGE and if response to F5.0==1**		
**D7.5**	Can your child use gestures to communicate?<br><br>உங்களுடன்தொடர்புகொள்வதற்குஉங்கள் குழந்தையால்சைகையைபயன்படுத்தமுடியுமா?	**D7.5.1|___| Code | குறியீடு**
**D7.5** Ask: *When your child wants to show you something, what does he/she do? When your child wants something, how does he/she tell you? When your child wants you to come to him/her, what does he/she do? Does he/she use any gestures to communicate to you?*		

**பிரிவு 7.5 -யையெப்படிநடத்தவேண்டும்:** உங்கள்குழந்தைஉங்களிடம்எதையோகாண்பிக்கவிரும்பினால், குழந்தைஎன்னசெய்வான்/ள்? உங்கள்குழந்தைக்குஏதாவதுதேவையென்றால்அதைகுழந்தைஎப்படிஉங்களிடம்தெரிவிப்பான்/ள்? நீங்கள்அருகில்வரவேண்டுமெனவிரும்பினால்உங்கள்குழந்தைஎன்னசெய்யும்? உங்களுடன்பேசுவதற்குஉங்கள்குழந்தைசைகைஎதையாவதுபயன்படுத்துமா?		
**Understanding words | வார்த்தைகளைபுரிந்துக்கொள்ளுதல்**		
**D7.6**	Can your child understand when told "no"?<br><br>உங்கள்குழந்தையால் "இல்லை" என்றுசொல்வதைபுரிந்துக்கொள்ளமுடியுமா?	**D7.6.1|___| Code | குறியீடு**
**How to administer7.6** Ask: *What does your child do when you say 'no'? Does he understand when you tell him no?*<br><br>**பிரிவு 7.6 -யையெப்படிநடத்தவேண்டும்:** நீங்கள் 'இல்லை' என்றுசொல்லும்போதுஉங்கள்குழந்தைஎன்னசெய்யும்? நீங்கள்இல்லைஎன்றுசொல்வதுகுழந்தைக்குபுரியுமா?		
**D7.7**	Can your child understand simple instructions like "come here" or "go away"?<br><br>"இங்கேவா" அல்லது "தூரம்போ" எனும்எளியகாரியங்களைசெய்யசொன்னால்உங்கள்குழந்தையால்புரிந்துக்கொள்ளமுடியுமா?	**D7.7.1|___| Code | குறியீடு**
**How to administer7.7** Ask: *What does your child do when you say "come here"? or "go away"? Does he/she understand when you tell him/her to do something simple?*<br><br>**பிரிவு 7.7 -யையெப்படிநடத்தவேண்டும்:** "இங்கேவா" அல்லது "தூரம்போ" என்றுசொல்லும்போதுஉங்கள்குழந்தைஎன்னசெய்யும்? எளிமையாகஎதையாவதுசெய்யச்சொன்னால்குழந்தைபுரிந்துக்கொள்வானா/ளா?		
**D7.8**	Can your child identifyat least 1 familiar object?<br><br>உங்கள்குழந்தையால்குறைந்ததுஒருபழக்கப்பட்ட/தெரிந்த பொருளையாவதுஅடையாளம்காட்டமுடியுமா?	**D7.8.1|___| Code | குறியீடு**

**How to administer7.8**Ask:*How many objects can your child identify? If you ask your child to bring his shoes, does he go and get them? What else does your child know? Anything in the kitchen? If you ask your child to show you his spoon, does he know? His cup? Among his clothes? Anything at all?*<br><br>**பிரிவு 7.8 -யைஎப்படிநடத்தவேண்டும்:** உங்கள்குழந்தையால்எத்தனைபொருட்களைஅடையாளம்காணமுடியும்? உங்கள்குழந்தையைஅதன்ஷூக்களைகொண்டுவரும்படிநீங்கள்கூறினால், குழந்தைபோய்அதனைகொண்டுவருவானா/ளா? வேறுஎன்னவெல்லாம்உங்கள்குழந்தைக்குதெரியும்? சமயலறையிலுள்ளபொருட்கள்? ஸ்பூனைஅடையாளம்காட்டும்படிஉங்கள்குழந்தையிடம்கூறினால், அவனுக்கு/ளுக்குஅதுதெரியுமா? அவனது/ளதுகப்? அவனது/ளதுஉடைகள்? வேறெதாவது?		
**START WITH THE BELOW QUESTION IF THE CHILD IS LESS THAN 22-31 MONTHS OF AGE and if response to F5.0==1**		
**D7.9**	Can your child say one definite word?<br><br>உங்கள்குழந்தையால்ஒரேஒருஉறுதியானவார்த்தையைகூறமுடியுமா?	**D7.9.1|___| Code | குறியீடு**
**How to administer 7.9** Ask: *Have you heard your child say any words? Even if he/she doesn't get the sound of the word right, does he/she say any sounds to always mean the same thing?*<br><br>**பிரிவு 7.9 -யைஎப்படிநடத்தவேண்டும்:** உங்கள்குழந்தைஏதாவதுவார்த்தையைபேசிநீங்கள்கேட்டிருக்கிறீர்களா? அந்தவார்த்தையின்உச்சரிப்பைசரியாககூறமுடியவில்லைஎன்றாலும், அந்தபொருளைகுறிப்பிடுவதற்குஉங்கள்குழந்தைஎப்போதும்அந்தவார்த்தையைத்தான் சொல்கிறனா/ளா? (உதா: அம்மா, பூனை, சுசு)		
**D7.10**	Identifies at least 1 body part<br><br>குறைந்தபட்சம் 1 உடல்பாகத்தையாவதுஅடையாளம்காட்டுகிறான்/ள்	**D7.10.1|___| Code | குறியீடு**
**How to administer7.10** Ask: *Can your child identify any body parts? For example, if you ask "where's your eyes?" can he/she point to his/her eyes? What about his/her hair, nose, fingers, mouth, ears, hands, teeth, feet, head, any one thing?*<br><br>**பிரிவு 7.10 -யைஎப்படிநடத்தவேண்டும்:** உங்கள்குழந்தையால்உடல்பாகங்களைஅடையாளம்காட்டமுடியுமா? உதாரணத்திற்கு, நீங்கள் "உனதுகண்கள்எங்கேஇருக்கிறது?" என்றுகேட்டால்,		

குழந்தைதனதுகண்களைசுட்டிக்காட்டுகிறனா/ளா? அவனது/ளதுமுடி, மூக்கு, விரல்கள், வாய், காதுகள், கைகள், பற்கள், பாதம், தலைஆகியஇவற்றில்எதையாவதுஒன்றைஅடையாளம்காட்டுகிறானா/ளா?		
**START WITH THE BELOW QUESTION IF THE CHILD IS 32-38 MONTHS OF AGE and if response to F5.0==1**		
**D7.11**	Can your child identifyat least 10 familiar objects?<br><br>உங்கள்குழந்தையால்குறைந்தது 10 பழக்கப்பட்ட/தெரிந்தபொருட்களையாவதுஅடையாளம்காட்டமுடியுமா?	**D7.11.1|___| Code | குறியீடு**
**How to administer 7.11** Ask: *How many objects can your child identify? If you ask your child to bring his shoes, does he go and get them? What else does your child know? Anything in the kitchen? If you ask your child to show you his spoon, does he know? His cup? Among his clothes? Anything else?* If necessary, write the objects below then count the number of objects that the mother reports the child can identify.<br><br>**பிரிவு 7.11 -யைஎப்படிநடத்தவேண்டும்:**<br>உங்கள்குழந்தையால்எத்தனைபொருட்களைஅடையாளம்காணமுடியும்? உங்கள்குழந்தையைஅதன்ஷூக்களைகொண்டுவரும்படிநீங்கள்கூறினால், குழந்தைபோய்அதனைகொண்டுவருகிறானா/ளா? வேறுஎன்னவெல்லாம்உங்கள்குழந்தைக்குதெரியும்? சமயலறையிலுள்ளபொருட்கள்? ஸ்பூனைஅடையாளம்காட்டும்படிஉங்கள்குழந்தையிடம்கூறினால், அவனுக்கு/ளுக்குஅதுதெரியுமா? அவனது/ளதுகப்? அவனது/ளதுஉடைகள்? வேறெதாவது? குழந்தையால்அடையாளம்காணமுடியும்என்றுஅம்மாகூறும்பொருட்களின்பெயர்களை தேவையென்றால்கீழேஎழுதிஅவற்றைகணக்கிடவும்.		
**D7.12**	Can your child name at least 1 familiar object?<br><br>உங்கள்குழந்தையால்குறைந்தது 1 பழக்கப்பட்டபொருளின்பெயரையாவதுகுறிப்பிடமுடியுமா?	**D7.12.1|___| Code | குறியீடு**
**How to administer 7.12** Ask: *How many objects can your child name? If you point to his shoes and ask your child “what’s that” what does he say? If you point to a cup and say “what’s that” what does he say? Anything else? Any one thing?*		

**பிரிவு 7.12 -யைஎப்படிநடத்தவேண்டும்:** உங்கள்குழந்தையால்எத்தனைபொருட்களின்பெயரைகுறிப்பிடமுடியும்? உங்கள்குழந்தையின்ஷூக்களைகாண்பித்துஇதுஎன்னஎன்றுகேட்டால், குழந்தைஎன்னசொல்வான்/ள்?		
		**Response | பதில்**
**D7.13**	Can your child say at least 10 words?<br>உங்கள்குழந்தையால்குறைந்தபட்சம் 10 வார்த்தைகளைசொல்லமுடியுமா?	**D7.13.1|___| Code | குறியீடு**

How to administer 7.13 and 7.19 Ask: *Have you heard your child say any words? Even if he/she doesn't get the sound of the word right, does he/she say any sounds to always mean the same thing? For example, if the child sees a chicken he/she says "ki" or if the child wants to go to the toilet he/she says "ca." Or, the child might also pronounce the word well. How many words does he/she say? Any words for animals? What words? Any words for things you have in the kitchen or around the house? What words? Any words for things that are outside? Any words for people? Any foods? Any clothes? Any body parts?* If necessary, write the words below then count the number of words the mother tells you that the child says.

பிரிவு 7.13 மற்றும் 7.19 யைஎப்படிநடத்தவேண்டும்:
உங்கள்குழந்தைஏதாவதுவார்த்தையைபேசிநீங்கள்கேட்டுள்ளீர்களா? அந்தவார்த்தையின்உச்சரிப்பைசரியாககூறமுடியவில்லைஎன்றாலும், அந்தபொருளைகுறிப்பிடுவதற்குஉங்கள்குழந்தைஎப்போதும்அந்தவார்த்தையைத்தான் சொல்கிறனா/ளா? உதாரணத்திற்கு, குழந்தைஒருகோழியைபார்த்துஅதை "கீ" என்றுஅல்லதுகுழந்தைவெளிக்குபோகவேண்டுமென்றால், அதை "கா" என்றுசொல்கிறதா. அல்லது, குழந்தைஅந்தவார்த்தையைநன்றாகவும்உச்சரிக்கலாம். குழந்தைஎத்தனைவார்த்தைகளைகூறுகிறான்/ள்? விளங்குகளைகுறிக்கும்ஏதாவதுவார்த்தை? என்னவார்த்தைகள்? சமயலறைஅல்லதுவீட்டைசுற்றியுள்ளபொருட்களைகுறிக்கும்ஏதாவதுவார்த்தைகள்? என்னவார்த்தைகள்? வீட்டிற்குவெளியேஉள்ளபொருட்களைகுறிக்கும்வார்த்தைகள்? மக்களைகுறிக்கும்ஏதாவதுவார்த்தைகள்? ஏதாவதுஉணவுகள்? ஏதாவதுஉடைகள்? ஏதாவதுஉடல்பாகங்கள்? குழந்தையால்கூறமுடியும்என்றுஅம்மாகூறும்பொருட்களின்பெயர்களைதேவையென்றால்கீழேஎழுதிஅவற்றைகணக்கிடவும்.

D7.14	Can your child imitates animal and other sounds, e.g., mee for a goat, moo for a cow, vroom for car?<br><br>உங்கள்குழந்தையால்மிருகங்கள்/விளங்குள்மற்றும்இதரஒலி களை/சப்தம்குரள்போலிசெய்யமுடிகிறதா. எ.கா., ஆடுகளுக்குமேஹ, பசுவிற்குமா, காருக்குரூம்ஒலிகள்/சப்தம்?	**D7.14.1|___| Code | குறியீடு**
**How to administer7.14** Ask: *When your child sees a goat, does he/she make the "mee" sound that a goat makes? When he/she sees a dog, does he/she make the "wowo" sound that a dog makes? Does he/she make the sound a car makes? Any other sounds?*<br><br>**பிரிவு 7.14 -யைஎப்படிநடத்தவேண்டும்:** உங்கள்குழந்தைஒருஆடுகாணும்போது, ஆடுசெய்யும் "மேஹ.. மேஹ.. மேஹ.." ஒலியை/சப்தம்ஏற்படுத்துகிறனா/ளா? குழந்தைஒருநாயைகாணும்போது, நாய்கள்செய்யும் "பௌ.. பௌ.. பௌ.." ஒலியை/சப்தம்ஏற்படுத்துகிறனா/ளா? குழந்தைகாரின்ஒலியை/சப்தம்எற்படுத்துகிறனா/ளா? வேறெதாவதுஒலிகள்/சப்தம்?		
7.15	Identifies at least 5 body parts<br><br>குழந்தைகுறைந்தது 5 உடல்பாகங்களைஅடையாளம்காட்டுகிறது	**7.15.1|___| Code | குறியீடு**
**How to administer 7.15 and 7.18**Ask:*Can your child identify body parts? For example, if you ask "where's your eyes?" can he/she point to his/her eyes? What about his/her hair, nose, fingers, mouth, ears, hands, teeth, feet, head, anything else?* Write the body parts below then count the number of body parts the mother reports the child can identify.<br><br>If necessary, write the body parts below then count the number of body parts the child can identify.<br><br>**பிரிவு 7.15 மற்றும் 7.18 -யைஎப்படிநடத்தவேண்டும்:** உங்கள்குழந்தையால்உடல்பாகங்களைஅடையாளம்காட்டமுடியுமா? உதாரணத்திற்கு, நீங்கள் "உனதுகண்கள்எங்கேஇருக்கிறது?" என்றுகேட்டால், குழந்தைதனதுகண்களைசுட்டிக்காட்டுகிறனா/ளா? அவனது/ளதுமுடி, மூக்கு, விரல்கள், வாய், காதுகள், கைகள், பற்கள், பாதம், தலைஆகியஇவற்றில்எதையாவதுஒன்றைஅடையாளம்காட்டுகிறானா/ளா? குழந்தையால்அடையாளம்காட்டமுடியும்என்றுஅம்மாகூறும்உடல்பாகங்களின்பெயர்க ளைகீழேஎழுதிஅவற்றைகணக்கிடவும்.		

குழந்தையைபார்த்துகூறவும்: உங்ககண்எங்கேஇருக்கு? ...மூக்கு? ...பாதம்? ...முடி? ...வாய்? ...காதுகள்? இவற்றில்எத்தனைபாகங்களைஅவனால்/ளால்அடையாளம்காட்டமுடிந்தது?<br><br>ஒருவேளைகுழந்தை 5 க்கும்மேற்பட்டஉடல்பாகங்களைஅடையாளம்காட்டினால், மேலும்தொடரவும்:<br><br>உங்கதலைஎங்கேஇருக்கு? ...கால்கள்? ...கைகள்? ...விரல்கள்? ...பற்கள்? ...கட்டைவிரல்? ...கால்விரல்கள்?<br><br>குழந்தையால்அடையாளம்காணமுடியும்என்றுஅம்மாகூறும்உடல்பாகங்களின்பெயர்களைதேவையென்றால்கீழேஎழுதிஅவற்றைகணக்கிடவும்.		
**D7.16**	Can your child name at least 10 familiar objects?<br><br>உங்கள்குழந்தையால்குறைந்தது 10 பழக்கப்பட்ட/தெரிந்தபொருட்களின்பெயர்களையாவதுகூற முடியுமா?	**D7.16.1|___| Code | குறியீடு**
**How to administer D7.16** Ask: *How many objects can your child name? If you point to his shoes and ask your child "what's that" what does he say? If you point to a cup and say "what's that" what does he say? Anything else?*<br><br>**பிரிவு 7.16 -யைஎப்படிநடத்தவேண்டும்:**<br>உங்கள்குழந்தையால்எத்தனைபொருட்களின்பெயர்களைகுறிப்பிடமுடியும்? உங்கள்குழந்தையிடம்அதன்ஷூக்களைகாண்பித்து, "இதுஎன்ன" என்றுகேட்டால், குழந்தைஎன்னசொல்வான்/ள்? ஒருகப்பைகாண்பித்து, "இதுஎன்ன" என்றுகேட்டால்குழந்தைஎன்னசொல்வான்/ள்? வேறெதாவது?		
**D7.17**	Can your child use two-word combinations?<br><br>உங்கள்குழந்தையால்இருவார்த்தைகளைசேர்த்துபயன்படுத்த/கூறமுடியுமா?	**D7.17.1|___| Code | குறியீடு**
**How to administer D7.17 and D7.20** Ask: *If your child wants something, how does he/she say it? What if she doesn't want you to do something? Have you heard him/her say two words together to try to make a sentence? Have you heard him/her say three words together to try to make a sentence?*<br><br>**பிரிவு 7.17 மற்றும் 7.20 -யைஎப்படிநடத்தவேண்டும்:**<br>உங்கள்குழந்தைக்குஏதாவதுதேவையென்றால்அதைகுழந்தைஎப்படிகூறுவான்/ள்? நீங்கள்எதையாவதுசெய்யக்கூடாதுஎன்றுகுழந்தைவிரும்பினால்குழந்தைஎன்னசெய்வான்		

?
குழந்தைதொடர்ச்சியாகஇரண்டுவார்த்தைகளைகூறுவதையோஅல்லதுஇருசொற்கள்சேர்க்கமுயற்சிப்பதையோநீங்கள்கேட்டுள்ளீர்ளா?
குழந்தைதொடர்ச்சியாகமூன்றுவார்த்தைகளைகூறுவதையோஅல்லதுஒருசொற்றொடரைஅமைக்கமுயற்சிப்பதையோநீங்கள்கேட்டுள்ளீர்ளா?

START WITH THE BELOW QUESTION IF THE CHILD IS 39-46 MONTHS OF AGE and if response to F5.0==1

**D7.18**	Can your child identify at least 10 body parts?<br><br>உங்கள்குழந்தையால்குறைந்தது 10 உடல்பாகங்களையாவதுஅடையாளம்காணமுடியுமா?	**D7.18.1|___| Code | குறியீடு**

How to administer 7.18 *See Item 7.15 above.*

பிரிவு 7.18 -யைஎப்படிநடத்தவேண்டும்: மேலேகொடுக்கப்பட்டுள்ள 7.15 -யைபார்க்கவும்

**D7.19**	Can your child say at least 50 words?<br><br>உங்கள்குழந்தையால்குறைந்தது 50 வார்த்தைகளைகூறமுடியுமா?	**D7.19.1|___| Code | குறியீடு**

How to administer 7.19*See item 7.13 above.*

பிரிவு 7.19 -யைஎப்படிநடத்தவேண்டும்: மேலேகொடுக்கப்பட்டுள்ள 7.13 -யைபார்க்கவும்

START WITH THE BELOW QUESTION IF THE CHILD IS GREATER THAN 46 MONTHS OF AGE and if response to F5.0==1

How to administer 7.20*See item 7.17 above.*

பிரிவு 7.20 -யைஎப்படிநடத்தவேண்டும்: மேலேகொடுக்கப்பட்டுள்ள 7.17 -யைபார்க்கவும்

**D7.20**	Can your child use three-word combinations?	**D7.20.1|___| Code | குறியீடு**

	உங்கள்குழந்தையால்மூன்றுவார்த்தைகளைசேர்த்துபயன் படுத்த/ கூறமுடியுமா?	
**Following Instructions | கட்டளைகளைபின்பற்றுதல்**		
		**Observation**
**D7.21**	Can your child follow three instructions together?<br>உங்கள்குழந்தையால்மூன்றுகட்டளைகளையும்பின்பற்ற முடிகிறதா?	**D7.21.1|___| Code | குறியீடு**
**How to administer 7.21** Ask:*If you Ask child to do three things can they remember and do all three?*<br>*Say to the child: Now I'm going to tell you to do something, are you ready to do it? Stand up and clap your hands and sit back down. See if the child can remember and do all three. Do not give the instructions one by one.*<br>**பிரிவு 7.21 -யைஎப்படிநடத்தவேண்டும்:** உங்கள்குழந்தையைமூன்றுவேலைகளைசெய்யும்படிநீங்கள்கூறினால், அவைகளைநினைவுவைத்துக்கொண்டுகுழந்தைசரியாகசெய்கிறானா/ளா?<br>குழந்தையைபார்த்துகூறவும்:<br>இப்போநான்உங்ககிட்டசிலவேலைகளைசொல்லப்போறேன், அதைசெய்றதுக்குநீங்கதயாரா? "எழுந்துநின்றுகைகளைதட்டிதிரும்பவும்நீஅமர்ந்திடு / ஊட்ககரவும்."<br>இந்துமூன்றுவேலைகளையும்நினைவில்வைத்திருந்துகுழந்தைசெய்கிறதாஎன்பதைகவனிக்கவும். இந்தகட்டளைகளைஒன்றன்பின்ஒன்றாககூறவேண்டாம்.		
		**Response**
**D7.22**	Can your child identify at least 3 colors?<br>உங்கள்குழந்தையால்குறைந்தது 3(கலர்)வண்ணங்களையாவதுஅடையாளம்காட்டமுடிந்ததா ?	**D7.22.1|___| Code | குறியீடு**
**D7.23**	Can your child identify at least 8 colors?<br>உங்கள்குழந்தையால்குறைந்தது 8 (கலர்)வண்ணங்களையாவதுஅடையாளம்காட்டமுடிந்ததா?	**D7.23.1|___| Code | குறியீடு**

How to administer 7.22-7.23 *Point to colored boxes and say to child: Show me something red. Now say show me something blue. Also try with green, yellow, orange, violet, white, black, brown, pink and grey. How many did he/she get right?*

பிரிவு 7.22-7.23 -யைஎப்படிநடத்தவேண்டும்:
வண்ணடப்பாக்களைகாண்பித்துகுழந்தையைபார்த்துகூறவும்:
சிவப்பைசுட்டிக்காட்டுபார்க்கலாம். இப்போநீலநிறத்தைசுட்டிக்காட்டுபார்க்கலாம்.
மேலும்பச்சை, மஞ்சள், ஆரஞ்சு, ஊதா, வெள்ளை, கருப்பு,
இளஞ்சிவப்புமற்றும்சாம்பல்நிறங்களையும்முயற்சிக்கவும்.
இவற்றில்எத்தனைநிறங்களைகுழந்தைசரியாககூறினான்/ள்?

		Observation
**D7.24**	Child follows 2 or more instructions<br><br>குழந்தை 2 அல்லதுஅதற்குமேற்பட்டகட்டளைகளைசரியாகபின்பற்றுகிறது	**D7.24.1|___| Code | குறியீடு**
**D7.25**	Child follows all four instructions<br><br>குழந்தைநான்குகட்டளைகளையும்சரியாகபின்பற்றுகிறது	**D7.25.1|___| Code | குறியீடு**

How to administer7.24-7.25 *Say to the child: Do these one by one.* Give these instructions one by one and see if the child can follow each one.

1 .Put your hands above head,

2. Put your fingers below your eyes.

3. Point to the center of this table/mat

4. Point to the corner of the table/mat

பிரிவு 7.24-7.25 -யைஎப்படிநடத்தவேண்டும்: குழந்தையிடம்கூறவும்: "இதஒவ்வொன்னாசெய்யனும்."
பின்வரும்கட்டளைகளைஒன்றன்பின்ஒன்றாககூறிகுழந்தையால்அதைசெய்யமுடிகிறதாஎன்பதைபார்க்கவும்.

1. தலைக்குமேலஉங்கைகைகளைவைக்கவும்
2. கண்களுக்குகீழஉங்கவிரல்களைவைக்கவும்
3. இந்தநாற்காலி/டேபல்நடுபகுதிகாண்பிங்கவும்

4. இந்தநாற்காலி /டேபல்கர்நார்/மூலையைபகுதிகாண்பிங்கவும்		
		**Response | பதில்**
**D7.26**	Child answers which goes faster for 2 or more questions<br><br>எதுவேகமாகபோகும்என்கிறகேள்விகளில்இரண்டுஅல்லது அதற்குமேற்பட்டகேள்விகளுக்குகுழந்தைபதிலளிக்கிறது.	**D7.26.1|___| Code | குறியீடு**
**How to administer 7.26** *Say to the child: Which goes faster, a person or a car? Which goes faster, a turtle or a horse? Which goes faster a bicycle or an airplane?* The child must answer at least two of the three questions correctly.<br><br>**பிரிவு 7.26 -யைஎப்படிநடத்தவேண்டும்:** குழந்தையைபார்த்துகூறவும்: எதுவேகமாபோகும், ஆளாஇல்லகாரா? எதுவேகமாபோகும், ஆமையாஇல்லகுதிரையா? எதுவேகமாபோகும், சைக்கிளாஇல்லவிமானமா? இந்தமூன்றுகேள்விகளில்குறைந்ததுஇரண்டுகேள்விகளுக்காவதுகுழந்தைகட்டாயம்பதில் கூறவேண்டும்?		
**D7.27**	Child can repeat 2 or more syllables (*pa, chi*)<br><br>குழந்தையால் 2 அல்லதுஅதற்குமேற்பட்டஅசைவொலிகளைதிரும்பகூறமுடிகிறது (பா, சி)	**D7.27.1|___| Code | குறியீடு**
**D7.28**	Child can repeat 4 syllables (*pa, chi, tu, go*)<br><br>குழந்தையால் 4 அசைவொலிகளையும்திரும்பகூறமுடிகிறது (பா, சி, து, கோ)	**D7.28.1|___| Code | குறியீடு**
**How to administer 7.27-7.28** *Say to the child. "When I say this, copy me....Pa, Chi, Tu, Go". Say to the child, say "pa", then say to the child "say pa, chi", then say to the child "say pa, chi, tu", then say to the child "say pa, chi, tu, go".* See how many the child can repeat.<br><br>**பிரிவு 7.27-7.28 -யைஎப்படிநடத்தவேண்டும்:** குழந்தையைபார்த்துகூறவும்: "நான்சொல்றதஅப்படிதிரும்பசொல்லுங்கபார்க்கலாம், பா, சி, து, கோ." குழந்தையைபார்த்துகூறவும், சொல்லுங்க "பா," பிறகுகுழந்தையைபார்த்துகூறவும், "சொல்லுங்கபா, சி," பிறகுகுழந்தையைபார்த்துகூறவும், "சொல்லுங்கபா, சி, து,"		

பிறகுகுழந்தையைபார்த்துகூறவும், “சொல்லுங்கபா, சி, து, கோ.” இதில்எத்தனைஒலிகளைகுழந்தையால்திரும்பகூறமுடிகிறதுஎன்பதைகவனிக்கவும்.		
**7.29**	Child answers what is the opposite for 2 or more questions<br>எதிர்சொற்கள்தொடர்பானகேள்விகளில்குழந்தை 2 அல்லதுஅதற்குமேல்பதில்கூறியது.	**7.29.1|___| Code | குறியீடு**
**How to administer 7.29***Say to the child; An ant is small, a goat is______. (big)*<br>*The sun comes up in the day, the moon comes up at ____. (night)*<br>*A baby is young, a grandma is ___________. (old)*<br>*The ground is down, the sky is ________. (up).*<br>*The coconut tree is tall, the plant is ______. (short)*<br>The child must answer at least two of the three questions correctly.<br>**பிரிவு 7.29 -யைஎப்படிநடத்தவேண்டும்:** குழந்தையிடம்கூறவும்: எறும்புசிறியது, ஆடு ____________. (பெரியது)<br>சூரியன்பகலில்வருகிறது, சந்திரன் ____________ வருகிறது. (இரவில்)<br>குழந்தைஇளமையானது, பாட்டி ____________ (வயதானவர்)		
**D7.30**	Child knows quantities 1, 2, and 3<br>குழந்தைக்குஎண்ணிக்கைதெரிகிறது - 1, 2 மற்றும் 3	**D7.30.1|___| Code | குறியீடு**
**D7.31**	Child knows quantities 4 and 5<br>குழந்தைக்குஎண்ணிக்கைதெரிகிறது - 4 மற்றும் 5	**D7.31.1|___| Code | குறியீடு**
**How to administer D7.30-D7.31**Put some coins in front of the child and *say to child, “give me 2 of the coins.”* Then ask for 1, 3, 5, and 4 of coins. Does he or she give you the right number of coins?<br>**பிரிவு D7.30-D7.31 -யைஎப்படிநடத்தவேண்டும்:**<br>சிலநாணயங்களைகுழந்தைக்குமுன்பாகவைத்துகுழந்தையைபார்த்துகூறவும்: “இதில்இரண்டுநாணயங்களைஎடுத்துக்கொடுபார்க்கலாம்.” தொடர்ந்து 1, 3, 5 மற்றும் 4 நாணயங்களைகேட்கவும்.<br>குழந்தைசரியானஎண்ணிக்கையில்நாணயங்களைஉங்களிடம்கொடுத்தானா/ளா?		

**D7.32**	Child knows which one does not belong for 1 or more questions<br><br>சம்மந்தமில்லாதவார்த்தைக்கானகேள்விகளில்குழந்தைக்கு 1 அல்லதுஅதற்குமேல்பதில்தெரிகிறது.	**D7.32.1|___| Code | குறியீடு**
**D7.33**	Child knows which one does not belong for all 3 questions<br><br>சம்மந்தமில்லாதவார்த்தைக்கானகேள்விகளில்குழந்தைக்குமூன்றுக்கும்பதில்தெரிகிறது.	**D7.33.1|___| Code | குறியீடு**
**How to administer 7.32-7.33***Say to the child: "I'm going to say four words. One does not belong. Listen and then tell me the words that does not belong." "**Chocolate, sugar, chalk, bread**." Did the child say "chalk"?*<br><br>*Try again with these: "**Bathroom, train, kitchen, bedroom**." Did the child say "train"?*<br><br>*Try again with these: "**Brother, helper, ladder, neighbor**." Did the child say "ladder"?*<br><br>**பிரிவு 7.32-7.33 -யைஎப்படிநடத்தவேண்டும்:** குழந்தையைபார்த்துகூறவும்: "இப்போநா 4 வார்த்தையைசொல்லப்போறேன். இதுலமத்தவார்த்தைகளோடசம்மந்தப்படாதஒருவார்த்தைசேர்ந்திருக்கும். இப்போநான்சொல்றதாநல்லாகவனிச்சுசம்மந்தமில்லாதவார்த்தையைசொல்லுங்கபார்க்கலாம்."<br><br>"**சாக்லேட், சர்க்கரை, சாக்பீஸ், பிரட்**" குழந்தை "சாக்பீஸ்" என்றுசொன்னதா?<br><br>"**குளியல்அறை, இரயில்வண்டி /டீராய்இன், சமயலறை, படுக்கைஅறை**" குழந்தை "இரயில்வண்டி" என்றுசொன்னதா?<br><br>"**சகோதரன்/அண்ணன், உதவியாளர்/சமாயல்கரன், ஏணி, பக்கத்துவீட்டார்**" குழந்தை "ஏணி" என்றுசொன்னதா?		
	TOTAL LANGUAGE SCORE = SUM OF RESPONSE ITEMS 7.1.1 - 7.33.1<br><br>மொத்தமொழிமதிப்பெண் = பதில்களின்கூட்டுத்தொகை (7.1.1 - 7.33.1)	|___|___|
**D8**	Do you have any concerns regarding the child's growth and development?<br><br>1 = **Yes, 0 = No**	**D8|___| Code | குறியீடு**

	If « yes », ask her to explain her concerns (briefly) and write them below :<br><br>-<br>குழந்தையின்வளர்ச்சிமற்றும்முன்னேற்றத்தைகுறித்துஉங்களுக்குஏதாவதுமனக்குறைஉள்ளதா?<br><br>**1=ஆம், 0=இல்லை**<br><br><<ஆம்>>என்றால், அம்மாவின்மனக்குறைகளைசுருக்கமாகவிவரிக்ககூறிஅதைகீழேகுறிப்பெடுத்துக்கொள்ளவும்:	
**D8.1**	Comments/observations | பின்குறிப்புகள்/கண்காணித்தவைகள்:<br><br>Instruction to data team: Make this question optional	

7B: WPPSI

Item administration - Sit across from the child and place the book on the table between you with the pictures facing the child. Give the child a pencil with an eraser which they can use to point to the images. They should not use their fingers. Read each item verbatim to the child as often as necessary, but do not alter the wording in any way. Always use the standard or local word as written. Use the local pronunciation of each word. Children of all ages should start with item 1 which is a teaching item. This is the only item which allows corrective feedback (i.e. tell the child what the item is if they give a wrong answer). It should be administered as follows: open up to item 1 and say **"Show me the foot"**. If the child says *eye* or points to the eye, point to the correct image and say **"This is the foot."** If the child points to the foot and says *hand*, say **"You pointed to the foot and you said eye. Which one did you mean?"** If they say *foot*, point to the correct image. and say **"Yes, this is the foot".**

குறிப்புநிர்வாகம் - குழந்தைக்குஎதிராகஅமர்ந்துகொண்டுஉங்களுக்கும்குழந்தைக்கும்இடையேஉள்ளமேஜையின்மீதுபுத்தகத்தைஅதிலுள்ளபடங்கள்குழந்தையைபார்த்திருக்கும்படிவைக்கவும். குழந்தைகள்படங்களைசுட்டிக்காட்டுவதற்குவசதியாகஅவர்களுக்குஇரப்ப

ருடன்கூடியஒருபென்சிலைகொடுக்கவும். அவர்கள்விரல்களைபயன்படுத்தக்கூடாது. ஒவ்வொருகுறிப்பையும்எத்தனைமுறைதேவைப்படுகிறதோஅத்தனைமுறையும்வார்த்தைமாறாமல்படித்துக்காண்பிக்கவும், ஆனாலும்எந்தவகையிலும்வார்த்தையைமாற்றிவிடவேண்டாம். எப்போதுமேஎழுதியுள்ளபடிநிலையானஅல்லதுபேச்சுவழக்கிலுள்ளவார்த்தையையேபயன்படுத்தவும். ஒவ்வொருவார்த்தைக்குமானபேச்சுவழக்குஉச்சரிப்பையேபயன்படுத்தவும் . அனைத்துவயதுகுழந்தைகளும்பாடம்கற்பிக்கும்குறிப்பாகிய 1 -லிருந்துதுவங்கவேண்டும். திருத்திக்கொள்ளக்கூடியபின்னூட்டைஅனுமதிக்கும்ஒரேகுறிப்புஇதுதான் (அதாவது, ஒருவேளைகுழந்தைஒருதவறானபதிலைகூறினால்அந்தபொருள்/குறிப்புஎன்னஎன்றுகுழந்தைக்குஎடுத்துக்கூறவும்)., சரியானபடத்தைசுட்டிக்காண்பித்துநீங்கள்கூறவேண்டியது: “இதுதான்பாதம்.” ஒருவேளைகுழந்தைபாதத்தைகாண்பித்துகைஎன்றுகூறினால், நீங்கள்கூறவேண்டியது: “நீபாதத்தைகாண்பித்துகைஎன்றுகூறுகிறாய். இதில்எதைநீகுறிப்பிடவிரும்புகிறாய்?” அதற்குஅவர்கள்பாதம்என்றுகூறினால், அவர்களுக்கு 1 மதிப்பெண்கொடுக்கவும், சரியானபடத்தைசுட்டிக்காட்டிநீங்கள்கூறவேண்டியது: “ஆமாம், இதுதான்பாதம்.”

Q. No.<br>கே. எண்	Question<br>கேள்வி	Answer Options<br>பதிலுக்கானதேர்வுகள்
W2	Child Name<br>குழந்தையின் பெயர்	--------
W4	Show me the **Foot**.<br>**பாதத்தை**காண்பிக்கவும்	1 **2** 3 4 DK | தெரியாது
W5	Show me the **Cup**.<br>**கப்பை**காண்பிக்கவும்	1 2 **3** 4 DK | தெரியாது

W6	Show me the **Pumpkin**.<br>**பூசணிக்காயை**காண்பிக்கவும்	1 2 3 **4** DK | தெரியாது
W7	Show me the **Butterfly**.<br>**பட்டாம்பூச்சியை**காண்பிக்கவும்	1 **2** 3 4 DK | தெரியாது
W8	Show me the **Elephant**.<br>**யானையை**காண்பிக்கவும்	1 **2** 3 4 DK | தெரியாது
W9	Show me the **Painting**.<br>**ஓவியத்தை**காண்பிக்கவும்	1 2 3 **4** DK | தெரியாது
W10	Show me the **Tubewell**.<br>**தண்ணீர்குழாயை**காண்பிக்கவும்	**1** 2 3 4 DK | தெரியாது
W11	Show me the **Spider**.<br>**எட்டுகால்பூச்சி /**<br>**சிலந்திப்பூச்சியை**காண்பிக்கவும்	1 **2** 3 4 DK | தெரியாது
W12	Show me **Raining**.<br>**மழையை**காண்பிக்கவும்	1 **2** 3 4 DK | தெரியாது
W13	Show me the **Matches**.<br>**தீப்பெட்டியை**காண்பிக்கவும்	**1** 2 3 4 DK | தெரியாது
W14	Show me the **Rickshaw**.<br>**ரிக்ஷாவண்டியை**காண்பிக்கவும்	1 2 **3** 4 DK | தெரியாது
W15	Show me the **Lamp**.<br>**விளக்கை**காண்பிக்கவும்	1 2 3 **4** DK | தெரியாது
W16	Show me **Kicking**.<br>**உதைப்பதை**காண்பிக்கவும்	1 2 **3** 4 DK | தெரியாது
W17	Show me the **Triangle**.<br>**முக்கோணத்தை**காண்பிக்கவும்	**1** 2 3 4 DK | தெரியாது
W18	Show me **Stirring**.<br>**கிளறுவது**காண்பிக்கவும்	1 2 3 **4** DK | தெரியாது
W19	Show me **Lying** down.<br>**படுத்திருப்பதை**காண்பிக்கவும்	**1** 2 3 4 DK | தெரியாது
W20	Show me **Carrying**.<br>**தூக்குவதை**காண்பிக்கவும்	**1** 2 3 4 DK | தெரியாது
W21	Show me the **Desert**.<br>**பாலைவனத்தை**காண்பிக்கவும்	1 **2** 3 4 DK | தெரியாது
W22	Show me **Diagnosing**.<br>**நோய்அறிவதை**காண்பிக்கவும்	1 **2** 3 4 DK | தெரியாது
W23	Show me the **Curly** tail.<br>**சுருண்டவாலை**காண்பிக்கவும்	1 2 **3** 4 DK | தெரியாது

W24	Show me the **Watch**.<br>**கைக்கடிகாரத்தை**காண்பிக்கவும்	1 2 3 **4** DK | தெரியாது
W25	Show me the **Binoculars**.<br>**பைனாக்குளரை**காண்பிக்கவும்	1 2 **3** 4 DK | தெரியாது
W26	Show me the bird **Beneath** the tree.<br>**மரத்திற்குகீழிருக்கும்பறவையை**காண்பிக்கவும்	yellow brown red **blue** DK | தெரியாது<br>மஞ்சள்பழுப்பு/ப்ரௌஞ்சிவப்பு**நீலம்**
W27	Show me the **Drums**.<br>**டிரம்ஸ்வாத்தியத்தை**காண்பிக்கவும்	1 2 **3** 4 DK | தெரியாது
W28	Show me **Ornamental (glittery)**.<br>**பளபளக்கும்ஆபரணங்களை**காண்பிக்கவும்	**1** 2 3 4 DK | தெரியாது
W29	Show me **Shaggy**.<br>**பரட்டைத்தலைமுடியை**காண்பிக்கவும்	1 **2** 3 4 DK | தெரியாது
W30	Show me **Balancing**.<br>**சமநிலையாய்** / **ப்யாலென்ஸ்சைவது**காண்பிக்கவும்	1 2 3 **4** DK | தெரியாது
W31	Show me **Bulldozer**.<br>**புல்டோசரை**காண்பிக்கவும்	1 **2** 3 4 DK | தெரியாது
W32	Show me the **Blackboard**.<br>**ப்ளாக்போர்ட்/கரும்பலகையை**காண்பிக்கவும்	1 2 **3** 4 DK | தெரியாது
W33	Show me **Gnawing**.<br>**கடிப்பதை**காண்பிக்கவும்	1 2 3 **4** DK | தெரியாது
W34	Show me **Planing**.<br>**சரிசமாம்ஆகுவது/**காண்பிக்கவும்	1 2 **3** 4 DK | தெரியாது
W35	Show me **Crouching**.<br>**குனிந்திருப்பதை**காண்பிக்கவும்	1 2 **3** 4 DK | தெரியாது
W36	Show me **Prancing**.<br>நடனம் / துள்ளு<br>காண்பிக்கவும்	1 2 3 **4** DK | தெரியாது
W37	Show me **Clenching**.<br>இறுக்கிபிடிப்பதுகாண்பிக்கவும்	1 **2** 3 4 DK | தெரியாது
W38	Show me **Parallel**.<br>**ப்யாரலெல்** / **இணையாகஇருப்பதை**காண்பிக்கவும்	1 **2** 3 4 DK | தெரியாது
W39	Show me the **Cylinder**.<br>**சிலின்டர்/உருளையை**காண்பிக்கவும்	1 2 **3** 4 DK | தெரியாது

W40	Show me **Equivalent**.<br>**சமமனது**காண்பிக்கவும்	1 **2** 3 4 DK | தெரியாது
W41	Show me **Horizontal**.<br>**ஹாரிஜாண்தால்/கிடைமட்டத்தை**காண்பிக்கவும்	1 2 **3** 4 DK | தெரியாது
W42	Total score (max 38)<br>மொத்தமதிப்பெண் (அதிகபட்சம் 38)	---------

7C Family Care Indicators

Instructions to data team:

Ask this section for all children between 6 months and 59 months in the household from the prefill and if response to F5.0==1. Ask this section after either 7A or 7B for each child.

Instructions to surveyors:

Say: "Now I would like to ask a few questions about your home environment."

**Q. NO. கே. எண்**	**QUESTIONS AND FILTERS**<br>**கேள்விகளும் வகைப்பிரித்தலும்**	**CODING CATEGORIES**<br>**குறியீட்டுவகைகள்**			**Coding Instruction**
F5.3	I am interested in learning about the things that [name of child] plays with when s/he is at home.<br>(குழந்தையின் பெயர்) வீட்டிலிருக்கும் போதுகுழந்தை படிப்பதும்மற்றும்விளையாடும் பொருட்களை குறித்துதெரிந்துக்கொள்வதி		Spontaneous<br>அவர்களே சுயமாக சொன்னவை	Probing<br>படித்துக் காண்பித்த பிறகு சொன்னவை	**One the first screen, show the question with "Spontaneous" written below and show all the answer options as multiple choice. If No is selected then don't allow any**
		None ..01<br>எதுவுமில்லை			

	ல்எனக்குஆர்வ ம்இருக்கிறது.<br><br>INSTRUCTIONS: First ask what all does the child play with and then check all those items that are recalled spontaneously by the mother. Then read whatever is left from the list and circle those she says yes to in the PROBING column. Finally request the mother if you can see any of the stated items that the				**option to be selected. On the next screen, show the same question with "Probing" written below and then show the answer options that were not selected in the previous question as multiple choice. Do not show the option "no" for probing.**
		Household objects (e.g. bowls, plates, cups) ..02<br>வீட்டுப்பொருட்கள் (எ.கா., கிண்ணம், தட்டு, கப்பு)			
		Objects and materials found outside the living quarters (e.g. sticks, rocks, animals, shells, leaves) ..03<br>வசிக்கும்வீட்டிற்குவெளியேஇருக்கக் கூடியபொருட்கள் (எ.கா., குச்சிகள், கற்கள், விளங்குகள், கிளிஞ்சல், இலை)			
		Homemade toys (e.g. dolls made from scraps of cloth, cars made with wood and wire) ..04<br>வீட்டில்செய்யப்பட்டபொம்மைகள் (எ.கா.,			

	child plays with and if shown, also circle the Observed column.<br><br>**குறிப்புகள்**:முதலில்எதையெல்லாம்அந்தகுழந்தைவிளையாடுகிறதுஎன்பதைகேட்டறிந்துமற்றும்அக்குழந்தையின்தாயால்அனைத்தையும்உடனடியாகநினைவுகூறமுடிகிறதாஎன்பதைசோதித்துபார்க்கவும்.<br>பிறகுபட்டியலிலுள்ளவிடுபட்டஅனைத்தையும்படித்துக்காண்பித்து, அதில்எதற்கெல்லாம்அவர்ஆம்என்றுகூறுகிறாரோஅவற்றைஆய்வுகாலம்/பத்தியில்வட்டமிடவும்.<br>இறுதியாகபட்டியலிலுள்ளஏதாவதுவிளையாட்டுகளைகுழந்தைவிளையாடுவதாகஅத	கிழிந்ததுணிகளைவைத்துதயாரித்த பொம்மைகள், மரத்தையும்ஒயர்களையும்கொண்டுதயாரித்தகார்கள்)			
		Store-bought children's toys 05<br><br>Things that make or play music, not just noise (such as instruments and toys that play melodies) ..05<br><br>கடையில்வாங்கியவிளையாட்டுபொம்மைகள்.<br>வெறும்சத்தத்தைமட்டும்உருவாக்காமல்இசையைஉருவாக்கும்பொருட்கள் (அதாவதுமெல்லிசையைஉருவாக்கும் வாத்தியங்கள்மற்றும்பொம்மைகள்)			
		Things for drawing and writing (e.g. coloring books, pencils, pens) ..06<br><br>ஓவியம்வரைவதற்கும்எழுதுவதற்கும் பயன்படும்பொருட்கள் (எ.கா., வண்ணம்தீட்டும்புத்தகம், பென்சில், பேனா)			
		Books meant for children including picture books (not including school books) ..07<br><br>படப்புத்தகங்கள்உள்ளிட்டகுழந்தைபுத்தகங்கள் (பள்ளிக்கூடபுத்தகங்கள்சேர்கமால்கூருவும்)			
		Things that are meant for			

	ன்தாய்பார்த்துள்ளராஎன்பதைகேட்டறிந்துஅதைகண்காணிக்கப்பட்டதுஎனும்பத்தியில்வட்டவும்.	stacking/constructing/building (e.g. blocks) ..08<br>கட்டமைப்பு/கட்டிடம்/ஒருங்கிணைப்பு போன்றவற்றைஉருவாக்குவதற்குபயன்படும்பொருட்கள் (எ.கா., பிளாக்ஸ்)				
		Things for moving a lot (balls, skipping rope, bats, rope for swinging, pull-along and push along toys) ..9<br>ஓடிஆடுவதற்குஉதவும்பொருட்கள் (பந்து, ஸ்கிப்பிங்கயிறு, பேட், ஊஞ்சல்கயிறு, தள்ளிவிடும்அல்லதுஇழுத்துவிடும் பொம்மைகள்)				
		Toys for shapes and colors 12<br>வடிவம்மற்றும்நிறங்களுக்கானபொம்மைகள்.				
		Other *(when in doubt write here)*<br>மற்றவை (சந்தேகமிருக்கையில்இங்கேகுறிப்பிடவும்)				
		Don't know / don't remember ..98<br>தெரியாவது / நினைவில்லை				

<table>
<tr><td></td><td colspan="3">

FOR QUESTIONS F9. 4 TO F9. 5, ASK THE RESPONDENT TO THINK OF THE PAST 3 DAYS WHEN ANSWERING THESE QUESTIONS.

கேள்விகள் 5.4 முதல் 5.9 வரையிலானகேள்விகளுக்குபதில்கூறும்போதுகடந்தமூன்றுநாட்களைநினைவுப்படுத்திபார்க்கும்படிபதில்அளிப்பவரிடம்கேட்கவும்.

"IN THE PAST 3 DAYS, DID YOU OR ANY FAMILY MEMBER OLDER THAN 15 YEARS, DO ANY OF THE FOLLOWING ACTIVITIES?"

"கடந்தமூன்றுநாட்களில், நீங்களோஅல்லது 15 வயதுக்குமேற்பட்டவேறுகுடும்பஉறுப்பினரோபின்வரும்எந்தநடவடிக்கையிலாவதுஈடுபட்டீர்களா?"

</td></tr>
<tr><td>F5.4</td><td>Read books or look at picture books with [name of child]
(குழந்தையின் பெயர்) - உடன்படப்புத்தகம்புத்தகம்அல்லதுபுகைப்பட/பொம்மைபார்த்தீர்களாவாசித்தீர்களா / படித்தீர்களா?</td><td>Yes | ஆம் ..1
No | இல்லை ..2
Sometimes | சிலநேரங்களில் ..3
Don't know / don't remember ..98
தெரியாது / நினைவில்லை 98</td><td></td></tr>
<tr><td>F5.5</td><td>Tell stories to (NAME OF CHILD)?
(குழந்தையின் பெயர்) - க்குகதைசொன்னீர்களா?</td><td>Yes | ஆம் ..1
No | இல்லை ..2
Sometimes | சிலநேரங்களில் ..3
Don't know / don't remember ..98
தெரியாது / நினைவில்லை</td><td></td></tr>
</table>

F5.6	Sing songs with [name of child]?<br>(குழந்தையின் பெயர்) - உடன்சேர்ந்து பாட்டுபாடினீர் களா?	Yes | ஆம் ..1<br>No | இல்லை ..2<br>Sometimes | சிலநேரங்களில் ..3<br>Don't know / don't remember ..98<br>தெரியாது / நினைவில்லை 98	
F5.7	Take [name of child] outside the home place?<br>(குழந்தையின் பெயர்) - யைவீட்டிலிருந் துவெளியேகூட் டிச்சென்றீர்க ளா?	Yes | ஆம் ..1<br>No | இல்லை ..2<br>Sometimes | சிலநேரங்களில் ..3<br>Don't know / don't remember ..98<br>தெரியாது / நினைவில்லை	
F5.8	Play with [name of child] with toys?<br>(குழந்தையின் பெயர்) - உடன்பொம் மைவைத்துவி ளையாடினீர்க ளா?	Yes | ஆம் ..1<br>No | இல்லை ..2<br>Sometimes | சிலநேரங்களில் ..3<br>Don't know / don't remember ..98<br>தெரியாது / நினைவில்லை 98	
F5.9	Spend time with [name of child] in naming things, counting, or drawing?<br>பொருட்களை அடையாளம்	Yes | ஆம் ..1<br>No | இல்லை ..2<br>Sometimes | சிலநேரங்களில் ..3<br>Don't know / don't remember ..98<br>தெரியாது / நினைவில்லை 98	

	காணுதல், எண்ணுதல், அல்லதுவரைதல் /ஓவியம்தீட்டுதல்போன்றகாரியங்களில் (குழந்தையின் பெயர்) - உடன்நேரம்செலவிட்டீர்களா?		

9.0 List of eligible members:<br><br>Instruction to surveyors: Write this on the back of the consent sheet: ஒப்புதல் படிவத்தின் பின்பக்கம் இதை எழுதவும்:<br><br>Instruction to data team: Display the member ID, name and age (with years/months mentioned) of the list of eligible members. Take name and ID from pre-fill and calculate age from 0.26/0.28/0.29/F5.21 (whichever one applies).Skip to 9.0B after this			
9.0A Instruction to surveyor: Please talk to the supervisor before going ahead<br>ஆய்வாளருக்கான குறிப்பு : தொடர்வதற்கு முன் தயவு செய்து மேற்பார்வையாளரிடம் பேசவும்.			
9.0A2	Replacement household ID	__________<br><br>No replacement household ID	6 digit numeric code. Check if this household ID is a replacement ID (i.e.- replacement_hh=1) and if it exists in the FPS code. If not, show error message "Wrong replacemet household ID"<br>Should NOT be optional
9.0B	Instruction to surveyor: please record **GPS Coordinates** of the household. You need to be out in the open or near a window in order to		Optional

	record the most accurate location and you should aim for an accuracy of up to 20 meters.<br><br>ஆய்வாளருக்கானகுறிப்பு: இந்தவீடுஅமைந்திருக்குமிடத்திற்குரிய ஃPஷ் புள்ளிகளைபதிவுசெய்யவும். அந்த ஃPஷ் பதிவில்துல்லியமானஇடத்தைகுறிக்கநீங்கள்வீட்டின்ஜென்னலோரத்திலோஅல்லதுவீட்டிற்குவெளியிலோஇருக்கவேண்டும். உ0 மீட்டர்துல்லியத்தைஎட்டுவதற்குநீங்கள் முயற்சிக்கவேண்டும்.		
9.0C	Which visit was this?<br>இந்தசந்திப்புமுதலாவதா, இரண்டாவதாஅல்லதுமூன்றாவதா?	1. First Visit<br>முதல்சந்திப்பு<br>2. Second Visit<br>இரண்டாம்சந்திப்பு<br>3. Third Visit<br><br>மூன்றாவதுசந்திப்பு	
9.0D	Is this form complete? இந்த படிவம் நிறைவடைந்துவிட்டதா?	1 Yesஆம்<br>0 No<br>இல்லை	Go to 9.0F if '1'<br>Go to 9.0E if '0'

9.0E Instruction to data team: Display the following message on screen and don't allow the surveyors to move forward.

2. if they are actually eligible (H0.13=1)
3. if they are still living in the household (i.e if response to H0.12=1)

Press the back button on the bottom of the tablet, select 'save changes', and move to the next household. If the appointment is on the same day, keep a note of this and revisit the household on the same day.
டேப்ளட்டின் கீழ்ப்புறத்தில் காணப்படும் 'back' பொத்தானை அழுத்தவும், 'save changes' -யை தேர்வை செலக்டு செய்துவிட்டு அடுத்த வீட்டிற்கு நேராக கடந்து செல்லவும். ஒருவேளை ஒதுக்கப்படும் சந்திப்பு நேரம் அதே நாளில் இருக்குமெனில், அதை குறிப்பெடுத்து வைத்துக்கொண்டு அந்த வீட்டை அதே நாளில் திரும்ப வந்து சந்திக்கவும்.

9.0F	Comments	Optional<br>Text entry

END OF QUESTIONNAIRE 1
