# Supplementary material for: Agreement between dried blood spots and HemoCue in Tamil Nadu, India
Source: Sci Rep. 2021 Apr 29;11:9285. doi: 10.1038/s41598-021-88425-y (PMC8085154; doi:10.1038/s41598-021-88425-y)
Supplement: Supplementary file 2 — Supplementary Information 2. [file 41598_2021_88425_MOESM2_ESM.pdf]

Agreement between two field-friendly methods of hemoglobin assessment: dried blood spots and HemoCue among females 12 to 40 years of age and children 6 to 59 months in Tamil Nadu, India

Reshma P Roshania[1], Rukshan V Mehta[1], Ashwini Shete[2], Aprajit Mahajan[3], Grant Miller[4], Alessandro Tarozzi[5], Reynaldo Martorell[1,6]*

*Corresponding Author: rmart77@emory.edu. Reynaldo Martorell, PhD, Hubert Department of Global Health, Rollins School of Public Health, Emory University, 1518 Clifton Road, NE, CNR 5005, Mailstop #1518-002-7BB, Atlanta, GA 30322 USA.

Tamil Nadu Rice Fortification Study | Baseline Questionnaire-2

தமிழ்நாடு ஊட்டச்சத்து நிறைத்த அரிசி குறித்த ஆய்வு | முதல் நிலை கேள்விப்பட்டியல்

Questionnaire 2

கேள்வித்தாள் 2

Table of contents:

பொருள் உள்ளடக்கம்:

H0. Identifying the location/household

இடம்/வீட்டை அடையாளம் காணுதல்

8. Anthropometric measures
   உடல் அளவுகள்
9. Hemocue Testing
   இரத்த பரிசோதனை
10. DBS Testing
    DBS **பரிசோதனை**

Section H0: Identifying the location/household

பிரிவு H0: இடம்/வீட்டை அடையாளம் காணுதல்

Q. No. கே.எண்	Question கேள்வி	Answer Options பதிலுக்கான தேர்வுகள்	Coding Instructions
H0.00	Date of first visit<br>முதல் சந்திப்பு தேதி		Date should be programmed to show up automatically
H0.01	Time of first visit<br>முதல் சந்திப்பு நேரம்		Time should be programmed to show up automatically
H0.02	Supervisor ID<br>மேற்பார்வையாளர் அடையாள எண்	_ _	
H0.03	Supervisor name<br>மேற்பார்வையாளர் பெயர்		We will provide a list of names with corresponding ID's so that once the ID is filled in, the name fills in
H0.04	Taluk name<br>தாலுகா பெயர்	1. Chidambaram<br>சிதம்பரம்<br>2. Bhuvanagiri<br>புவனகிரி	
H0.05	FPS area code<br>ரேஷன் கடையின் குறியீடு		Enter code. 3 digit Numeric<br>Check if this FPS code exists in the Taluk given in H0.04 in the pre-fill Error message should say "Wrong FPS code"
H0.05A	FPS area name<br>ரேஷன் கடையின் பெயர்		To automatically appear after entering ID. List of IDs and corresponding names to be shared with data team

H0.06	Where were these health measurements taken?<br>உடல் அளவுகள் எங்கே எடுக்கப்பட்டது?	1. Health camp<br>1. மருத்துவ முகம்<br>2.Household<br>2. வீட்டில்	If 1 then go to H0.06A If 2 then skip to H0.06B
H0.06A	Where is the camp taking place?<br>எங்கே முகாம் நடத்தப்பட்டது?	1. Anganwadi Center<br>1. அங்கன்வாடி மையம்<br>2. Taluk office<br>2. வட்டார அலுவலகம்<br>3. School<br>3. பள்ளி<br>4. Religious/community center<br>மதரீதியான/சமுதாயக்கூடம்<br>5. Home<br>5 வீட்டில் 6. Other<br>6. மற்றவை	
H.0.06B	Was the area cleaned well enough to conduct health measurements?<br>உடல் அளவுகள் எடுப்பதற்கு இந்த இடம் சுத்தமாக இருந்ததா?	0. No<br>0. இல்லை1. Yes<br>1. ஆம்	
H.0.06C	Was there flat ground to place the stadiometer/ infantometer and weighing machine?<br>ஸ்டேடியோ மீட்டர், இன்ஃபேன்டோ மீட்டர், எடை பார்க்கும் கருவிகள் சமமான தரையில் வைக்கப்பட்டதா?	0. No<br>0. இல்லை1. Yes<br>1. ஆம்2. Not sure<br>2. உறுதியாக சொல்ல முடியவில்லை	
H0.07A	Household ID<br>வீட்டு அடையாள எண்		.<br>Check to see if this household ID exists in the FPS area entered in H0.05. If it does

			not, show an error message “Wrong HHID”
H0.07A1	Ration card ID used to track this household<br>குடும்பஉறுப்பினருக்கு சொந்தமானரேஷன்அட்டைஅடையாளஎண்.<br>ஆய்வாளருக்கானகுறிப்பு: இந்தவீட்டைநீங்கள்கண்டுபிடிப்பதற்குஉதவியாகஉங்களிடம்கொடுக்கப்பட்டரேஷன்அட்டைவிவரங்களைஇங்குஉள்ளீடுசெய்யவும்.		Check if this ration card ID matches the Household ID entered in H 0.07A. If it does not, show error message “Ration card ID does not match Household ID” and don’t let the questionnaire move forward.
H0.07A2	Did the household agree to participate in the health camp?<br>இந்த வீட்டின் உறுப்பினர்கள் சுகாதார முகாமீள் பங்கேற்க ஒப்புக்கொண்டாரா?	0. No இல்லை<br>1. Yes ஆம்	Go to 10.04 if 0
H0.07B	Is this ration card holder’s name correct?<br>இந்த ரேஷன் கார்ட் வைத்திருப்பவரின் பெயர் சரியாக உள்ளதா?	0. No இல்லை<br>1. Yes ஆம்	Show the ration card info from the pre-fill data that matches the household ID from H0.07A. If ‘0’ is selected then show error message “Wrong ration card ID or household ID. Please check” and don’t let the questionnaire move ahead.

H0.12	I am going to read out some names. Please tell me if	1.Still living in the household	Repeat this question for every eligible member in the

	these members are still living in the household<br>**நான் சில பெயர்களை படிக்கப் போகிறேன். அவர்** இந்த வீட்டில் **வசிக்கிறாரா இல்லையா என்று என்னிடம் சொல்லுங்கள்.**<br>Is [name] still living in this household?<br>[name] அவர்கள் தற்சமயம் இந்த வீட்டில் வசிக்கிறாரா?	1.**இன்னும் இந்த வீட்டில் வசிக்கிறார்**<br>0.Not living in the household<br>0. இவர் இப்போ இந்த வீட்டில் வசிப்பதில்லை மாட்டார்	household in a roster form
H0.13	Is [name] eligible (i.e.- child between 6-59 months or a woman between 12-40 years)?<br>இவர் (பெயர்) நம் ஆய்வுக்கு தகுதியுடையவரா? (அதாவது 6-59 மாதங்கள் அல்லது 12-40 வயதுடைய பெண்)	1.Yes<br>1.ஆம்<br>0. No<br>இல்லை	Repeat this question for every eligible member (i.e.- mem_elig_child=1 OR mem_elig_wom=1) in the household in a roster form

Section H1: Menarche and pregnancy

<u>பிரிவு HI: பூப்பெய்துவது, கர்பமடைவது</u>

Instruction to data team:

Only ask this section H1 for women between the age 12-20 (refer prefill) and if they are still living in the household (i.e if response to H0.12=1)

Q. No.	Question	Answer Options	Coding Instructions
Instruction to the data team:<br>Display:<br>Name of the member<br>குடும்ப உறுப்பினர் பெயர்<br>ID of the member<br>உறுப்பினரின்அடையாளஎண்			

H1.00	Has [name]'s periods started?<br><br>(பெயர்) அவர்களின் மாதவிடாய் தொடங்கியுள்ளதா?	1.Yes<br>1.ஆம்<br><br>0. No<br>இல்லை<br><br>-99 Don't know<br>தெரியாது<br><br>-98 Refused to answer<br>பதில் கூற மறுத்துவிட்டார்	If 0, skip to the next section and don't ask H1.01-H1.03.<br><br>If -99/-98 then skip to H1.02
H1.01	What age did [name]'s periods start?<br>(பெயர்) எந்த வயதில் பூப்படைந்தார்?<br><br>Instruction to surveyor: If the respondent does not remember, encourage them to give you an approximate age<br>ஆய்வாளருக்கான குறிப்புகள்: தெரியவில்லையெனில் அவர்களை தோராயமான வயதை கூறும் மாறு சொல்லவும்.	______years<br>______வயது<br>-99 Don't know<br>தெரியாது<br>-98 Refused to answer<br>பதில் கூற மறுத்துவிட்டார்	Numeric entry between 8 and 20

Instruction to data team:

Only ask this section for women who are 18 years and older (refer prefill) and if they are still living in the household (i.e if response to H0.12=1) .

Q. No.	Question	Answer Options	Coding Instructions

H1.02	Is [name] married?<br>(பெயர்) அவர்களுக்கு திருமணம் ஆகிவிட்டதா?	0 No இல்லை<br>1 Yes ஆம்<br>-99 DK தெரியாது<br>-98 RA பதில் கூற மறுத்துவிட்டார்	If 0/-99/-98, skip to H1.04
H1.03	Is [name] currently pregnant?<br>(பெயர்) அவர்கள் தற்போது கர்ப்பமாக உள்ளாரா?	0. No இல்லை<br>1. Yes ஆம்<br>-99 DK தெரியாது<br>-98 RA பதில் கூற மறுத்துவிட்டார்	

Section H2:DBS

H2.02A	Which household ID is this household replacing?<br>எந்த குடும்பத்திற்கு பதிலாக இந்த குடும்ப அடையாள எண் மாற்றப்படுகிறது?	1. Enter Main list ID<br>2. Main list completed மெயின் லிஸ்ட் முடிந்துவிட்டது	Only ask this question if H0.07A is a replacement list ID (i.e.- from prefill, replacement_hh =1 for the ID entered in H0.07A)<br><br>If "Main list completed" is selected then directly go Section 8A.<br>If enter ID is selected then

			enter the ID that is:<br>6 digit numeric entry. Check if this household ID exists in the FPS code given in H0.05. If it does not, show error message “please check ID”<br>This HHID should not be the same as response in H0.07A. If it is the same then show error message and don’t let the questionnaire move forward.<br>The hhid entered here should from the main list (i.e.- from prefill, replacement_hh =0). If it is not then show error message and don’t let the questionnaire move forward.
H2.02B	Instruction to data team:		

	If the following criteria is met:<br>1. Eligible member (in pre-fill: mem_elig_child OR mem_elig_wom is 1)<br>2. Still living in the hh (H0.12=1)<br>3. from DBS household (section10_dbs=1 for the ID entered in H0.07A OR section10_dbs=1 for the ID entered in H2.02A)<br>4. Selected for DBS (in pre-fill: dbs_child OR dbs_wom is 1)<br>5. If they are actually eligible (H0.13=1)<br>display the name and id from the prefilled data , along with the following message:<br>**Instruction to surveyor:** These are the selected woman and/or child for the DBS sample. Please only take his/her/their DBS sample.<br>ஆய்வாளருக்கான குறிப்பு: இந்த பெயர்கள் உடைய பெண்கள் மற்றும்/அல்லது குழந்தைகள் டி.பி.எஸ் செய்ய தேர்தெடுக்கப்பட்டவர்கள். தயவு செய்து இவர்களுக்கு மட்டும் டி.பி.எஸ். எடுக்கவும்

Section 8A: Anthropometric Measures | Weight

பிரிவு 8A: உடல் அளவுகள்

Instruction to data team: Repeat Section 8A for ever eligible member in the hh (i.e.- mem_elig_child OR mem_elig_woman is 1) who is still living in the hh (i.e.- H0.12=1) AND If they are actually eligible (H0.13=1)

Instructions to surveyor:

ஆய்வாளருக்கான குறிப்புகள்:

This section must be repeated for all women between the age of 12-40 and children in the age group of 6 months to 59 months, in the presence of their mothers.

12 – 40 வயதுக்குக்குட்பட்ட அனைத்து பெண்களிடமும் மற்றும் 6 மாதங்கள் முதல் 59 மாதங்கள் வரை உள்ள அனைத்து குழந்தைகளிடமும் அவர்களுடைய அம்மாவின் முன்நிலையில் இந்த பிரிவிலுள்ள கேள்விகளை கேட்கவேண்டும்.

Weight will be measured using a flat scale.

ஒரு எடைக்கருவியை கொண்டு உங்கள் உடல் எடை அளவிடப்படும்.

Height will be measured using a stadiometer/ infantometer.

உயரம் ஸ்டேடியோ மீட்டர் / இன்ஃபேன்டோ மீட்டரை கொண்டு அளவிடப்படும்.

Read this out loud:

இதை பங்குகொள்பவருக்கு சத்தமாக படித்துக்காண்பிக்கவும்:

“In this section, we will take yours and the child’s height and weight. The participation is voluntary as before. It will take 15-20 minutes to take all the measurements. There are no risks of participating in this section of the survey. The only benefit your children will get from this survey is a health card with your height and weight written down. We are offering it for free."

“இப்போ, நாங்க உங்களுக்கும் குழந்தைக்கும் உயரம் மற்றும் எடையளவை எடுக்கப் போறோம். முன்னாடி சொன்னது போலவே இதுல உங்களுக்கு சம்மதம்னா மட்டும் தான் நாங்க உங்க எடை மற்றும் உயரத்தை அளவு எடுப்போம். இந்த அளவுகளையெல்லாம் எடுத்து முடிக்க 15-20 நிமிடங்கள் வரை ஆகும். இதுனால உங்களுக்கு எவ்வித பாதிப்பும் ஏற்படாது. இந்த ஆய்வின் மூலம் உங்களுக்கும் உங்கள் குழந்தைக்கும் கிடைக்கும் ஒரு நன்மை : உங்க உடல் எடை உயரம் இதெல்லாம் உங்களுக்கு நாங்கள் ஒரு அட்டையில் எழுதி கொடுப்போம். அதற்கு நாங்கள் உங்களிடம் பணம் எதுவும் வசூலிக்க மாட்டோம்.”

**Q. No.<br>கே.எண்**	**Question<br>கேள்வி**	**Answer Options<br>பதிலுக்கான தேர்வுகள்**	**Skip Pattern**
Instruction to the data team:<br>Display:<br>Name of the member<br>குடும்ப உறுப்பினர் பெயர்<br>ID of the member<br>உறுப்பினரின்அடையாளஎண்			
**8.00**	Is this person available for the survey right now?<br>இப்பொழுது இந்த நபர் சர்வேக்கு வீட்டில் இருக்கிறாரா?	0 No<br>இல்லை<br>1 Yes<br>ஆம்	**If 0 then go to the next person in the roster.**
**Instruction to enumerator:**<br>Read: “Now, we would like to measure your weight.”<br>**ஆய்வாளருக்கான குறிப்பு:**			

படித்துக்காட்டவும்: "இப்போ, உங்கள் எடையை நாங்கள் அளவிடப்போகிறோம்."			
8.01	Instructions to the surveyor: Do not read out this question. Fill this yourself.<br>ஆய்வாளருக்கான குறிப்பு: இந்த கேள்வியை படித்துக்காண்பிக்க வேண்டாம். இதை நீங்களாகவே நிரப்பிக்கொள்வும்.<br>Is the person being measured:<br>அளவெடுக்கப்படுபவர் யார்?:	0 Adult<br>பெரியவர்<br>1 Child who can stand with assistance/child who cannot stand<br>யாரேனும் ஒருவரது உதவியுடன் நிற்கக்கூடிய ஒரு குழந்தை/ தானாக நிற்க முடியாத குழந்தைகள்<br>2 Child who can stand without assistance<br>யாருடைய உதவியும் இல்லாமல் தனியே நிற்கக்கூடிய ஒரு குழந்தை	If 0, 2 move to 8.04<br>If 1, move to 8.10;<br><br>**validate with prefill**:<br>if 0 is selected in 8.01, mem_elig_wom=1 in the prefill<br>If 1/2 is selected for 8.01, mem_elig_child must be=1 in the prefill<br><br>If there is mismatch, display "incorrect entry. Check again"
8.04	Instructions to the surveyor: Find a flat surface to keep the scale and ask the respondent to remove all heavy materials such as purse, pens, jackets.<br><br>Trial 1: Record weight in kgs (to the nearest 0.1 kg)<br><br>ஆய்வாளருக்கான குறிப்பு:<br>• எடை கருவியை சமமான தரையில் வைக்கவும்<br>• பங்குகொள்ளும் நபர்களிடம் மிகவும் கனமான ஆபரணங்கள் / உடை ஏதாவது அணிந்திருந்தால்	1. Trial 1:<br>சோதனை 1:<br><br>□□□.□ kg<br>கிலோ<br><br>2. -98 Refused<br>ரெஸ்பான்டெண்ட் மறுத்துவிட்டார்	If 2, skip to 8.16<br><br>Weight should be with two numbers after decimal point<br><br>Restrict 5-200 kg (including 5 and 200)

	ஜாக்கெட், பர்ஸ்,பென், கொலுசு முதலியவை கழற்றிவைக்கவும்.<br>• எடைப் பதிவு: எடையை கிலோ கணக்கில் பதிவுசெய்யவும் (0.1 கிலோ துல்லியத்திற்கு பதிவுசெய்யவேண்டும்)		
30 second break between 8.04 and 8.05			
8.05	Trial 2: Record weight in kgs (to the nearest 0.1 kg)<br>சோதனை 2: எடையை கிலோ கணக்கில் பதிவுசெய்யவும் (0.1 கிலோ துல்லியத்திற்கு பதிவுசெய்யவேண்டும்)	1. Trial 2:<br>சோதனை 2:<br>□□□.□ kg<br>□□□.□<br>கிலோ<br>2. -98 Refused<br>மறுப்பு தெரிவித்தார்	If 2, skip to 8.07<br><br>If difference between 8.04 and 8.05 is more than 0.2kg then move to 8.06<br>Else, move to 8.07<br>Weight should be with two numbers after decimal point<br><br>Restrict 5-200 kg (including 5 and 200)
30 second break between 8.05 and 8.06			
8.06	Trial 3:<br>Record weight in kgs (to the nearest 0.1 kg)<br>சோதனை 3: எடையை கிலோ கணக்கில் பதிவுசெய்யவும் (0.1 கிலோ துல்லியத்திற்கு பதிவுசெய்யவேண்டும்)	1. Trial 3:<br>சோதனை 3:<br>□□□.□ kg<br>□□□.□<br>கிலோ<br>2. -98 Refused<br>மறுப்பு தெரிவித்தார்	Only ask if the difference between 8.04 and 8.05 is more than 0.2 kg<br><br>Weight should be with two numbers after decimal point<br><br>Restrict 5-200 kg (including 5 and 200)

8.07	Instructions to the surveyor: Do not read out this question. Fill this yourself.<br>ஆய்வாளருக்கான குறிப்பு: இந்த கேள்வியை படித்துக்காண்பிக்க வேண்டாம். இதை நீங்களாகவே நிரப்பிக்கொள்வும்.<br>Was the respondent wearing heavy materials (jacket, bracelet, heavy anklets, etc.) during the measurement?<br>எடை பார்க்கும் போது பங்குகொள்ளும் நபர் மிகவும் கனமான ஆபரணங்கள் / உடை ஏதாவது அணிந்திருந்தாரா (ஜாக்கெட், பிரேஸ்லேட் கொலுசு முதலியவை)?	1.Yes<br>ஆம்<br>0 .No<br>இல்லை	
8.09	Instructions to the surveyor: Do not read out this question. Fill this yourself.<br>ஆய்வாளருக்கான குறிப்பு: இந்த கேள்வியை படித்துக்காண்பிக்க வேண்டாம். இதை நீங்களாகவே நிரப்பிக்கொள்வும்.<br>Was the respondent able to stand absolutely straight?<br>பங்குகொள்ளும் நபரால் நேராக நிற்க முடிந்ததா?	0 No<br>இல்லை<br>1 Yes<br>ஆம்	End the section after this question
8.10	Instructions to the surveyor: Find a flat surface to keep the scale, and ask the respondent to remove all heavy materials such as purse, pens, jackets, heavy anklets<br>**ஆய்வாளருக்கான குறிப்பு:**<br>• எடை கருவியை சமமான தரையில் வைக்கவும்.	1. Trial 1a: Adult only:<br>சோதனை 1: பெரியவர் மட்டும்<br>□□□.□ kg<br>□□□.□ கிலோ<br>Trial 1b: Total adult and child:	Restriction: a) has to be lesser than b)<br><br>Weight should be with two numbers after decimal point<br><br>If refused to answer, skip to 8.16

	• பங்குகொள்ளும் நபர்களிடம் மிகவும் கனமான ஆபரணங்கள் / உடை ஏதாவது அணிந்திருந்தால் ஜாக்கெட், பர்ஸ்,பென், கொலுசு முதலியவை கழற்றிவைக்கவும்.<br>Trial 1:<br>**சோதனை** 1:<br>a) Record only adult weight (without child respondent) in kgs (to the nearest 0.1 kg)<br>பெரியவரின் எடையை மட்டும் (குழந்தையை சேர்க்காமல்) கிலோ கணக்கில் பதிவு செய்யவும் (0.1 கிலோ துல்லியத்திற்கு பதிவு செய்யவேண்டும்)<br>b) Now weigh adult and child together and record weight in kgs (to the nearest 0.1 kg)<br>இப்போது, பெரியவர் மற்றும் குழந்தை ஆகிய இருவரையும் ஒன்றாக சேர்த்து எடையை கிலோ கணக்கில் பதிவு செய்யவும் (0.1 கிலோ துல்லியத்திற்கு பதிவு செய்யவேண்டும்)<br>c) Calculated child respondent weight in kgs (to the nearest 0.1 kg)<br>குழந்தையின் எடை:	**சோதனை** 1: பெரியவர் மற்றும் குழந்தையின் மொத்த எடை<br>□□□.□ kg<br>□□□.□ கிலோ<br>Trial 1c: Child weight (calculate):<br>**சோதனை** 1: குழந்தையின் எடை (கணக்கிடவும்)<br>□□□.□ kg<br>□□□.□ கிலோ<br>2. -98 Refused<br>மறுப்பு தெரிவித்தார்	Restrict 5-200 kg (including 5 and 200)
30 second break between 8.10 and 8.011			
8.11	Trial 2:<br>**சோதனை** 2:<br>a) Record only adult weight (without child respondent) in kgs (to the nearest 0.1 kg)<br>பெரியவரின் எடையை மட்டும் (குழந்தையை சேர்க்காமல்) கிலோ	1 Trial 2a: Adult only:<br>**சோதனை** 2: பெரியவர் மட்டும்<br>□□□.□ kg<br>□□□.□ கிலோ	If 2 skip to 8.13<br><br>Weight should be with two numbers after decimal point

	கணக்கில் பதிவு செய்யவும் (0.1 கிலோ துல்லியத்திற்கு பதிவு செய்யவேண்டும்)<br>b) Now weigh adult and child together and record weight in kgs (to the nearest 0.1 kg)<br>இப்போது, பெரியவர் மற்றும் குழந்தை ஆகிய இருவரையும் ஒன்றாக சேர்த்து எடையை கிலோ கணக்கில் பதிவு செய்யவும் (0.1 கிலோ துல்லியத்திற்கு பதிவு செய்யவேண்டும்)<br>c) Calculated child respondent weight in kgs (to the nearest 0.1 kg)<br>குழந்தையின் எடை:	Trial 2b: Total adult and child: சோதனை 2: பெரியவர் மற்றும் குழந்தையின் மொத்த எடை<br>□□□.□ kg<br>□□□.□ கிலோ<br>Trial 2c: Child weight (calculate): சோதனை 2: குழந்தையின் எடை (கணக்கிடவும்)<br>□□□.□ kg<br>□□□.□ கிலோ<br>2. -98 Refused மறுப்பு தெரிவித்தார்	Only go to 8.12 if difference between 8.0c) and 8.10d) is greater than 0.2 kgs, Else, move to 8.13<br>Restriction: b) has to be lesser than c)<br><br>Restrict 5-200 kg (including 5 and 200)
30 second break between 8.11 and 8.12			
8.12	Trial 3:<br>சோதனை 3:<br>(if the difference between trials is more than 0.2 kg)<br>(மேற்காணும் இரண்டு சோதனைகளுக்கும் இடையே 0.2 கிலோ வேறுபாடு காணப்பட்டால்)<br><br>a) Record only adult weight (without child respondent) in kgs (to the nearest 0.1 kg)<br>பெரியவரின் எடையை மட்டும் (குழந்தையை சேர்க்காமல்) கிலோ கணக்கில் பதிவு செய்யவும் (0.1 கிலோ துல்லியத்திற்கு பதிவு செய்யவேண்டும்)	1.Trial 3a: Adult only:<br>சோதனை 3: பெரியவர் மட்டும்<br>□□□.□ kg<br>□□□.□ கிலோ<br>Trial 3b: Total adult and child:<br>சோதனை 3: பெரியவர் மற்றும் குழந்தையின் மொத்த எடை<br>□□□.□ kg<br>□□□.□ கிலோ<br>Trial 3c: Child weight (calculate):	Only ask if the difference between 8.10c) and 8.11d) is more than 0.2 kg<br><br>Weight should be with two numbers after decimal point<br><br>Restriction: b) has to be lesser than c)<br><br>Restrict 5-200 kg (including 5 and 200)

	b) Now weigh adult and child together and record weight in kgs (to the nearest 0.1 kg)<br>இப்போது, பெரியவர் மற்றும் குழந்தை ஆகிய இருவரையும் ஒன்றாக சேர்த்து எடையை கிலோ கணக்கில் பதிவு செய்யவும் (0.1 கிலோ துல்லியத்திற்கு பதிவு செய்யவேண்டும்)<br>c) Calculated child respondent weight in kgs (to the nearest 0.1 kg)<br>குழந்தையின்	சோதனை 3: குழந்தையின் எடை (கணக்கிடவும்)<br>□□□.□ kg<br>□□□.□ கிலோ<br>2. -98 Refused<br>மறுப்பு தெரிவித்தார்	
8.13	Instructions to the surveyor: Do not read out this question. Fill this yourself.<br>ஆய்வாளருக்கான குறிப்பு: இந்த கேள்வியை படித்துக்காண்பிக்க வேண்டாம். இதை நீங்களாகவே நிரப்பிக்கொள்வும்.<br>Was the child wearing heavy materials (jacket, bracelet, etc.) during the measurement?<br>எடை பார்க்கும் போது பங்குகொள்ளும் குழந்தை மிகவும் கனமான ஆபரணங்கள் / உடை ஏதாவது அணிந்திருந்ததா (ஜாக்கெட், பிரேஸ்லேட் முதலியவை)?	0 No<br>இல்லை<br>1 Yes<br>ஆம்	

Section 8B: Anthropometric Measures | Height

பிரிவு 8B: உடல் அளவுகள்

Instruction to data team: Repeat Section 8B for ever eligible member in the hh (i.e.- mem_elig_child OR mem_elig_woman is 1) who is still living in the hh (i.e.- H0.12=1) AND If they are actually eligible (H0.13=1)

Q. No.<br>கே.எண்	Question<br>கேள்வி	Answer Options	Skip Pattern

		பதிலுக்கான தேர்வுகள்	
Instruction to the data team:<br>Display:<br>Name of the member<br>குடும்ப உறுப்பினர் பெயர்<br>ID of the member<br>உறுப்பினரின்அடையாளஎண்			
**8.15**	Is this person available for the survey right now?<br><br>இப்பொழுது இந்த நபர் சர்வேக்கு வீட்டில் இருக்கிறாரா?	0 No<br><br>இல்லை<br><br>1 Yes<br><br>ஆம்	**If 0 then go to the next person in the roster.**

Instruction to enumerator: Say "Now we would like to measure your height."<br>**ஆய்வாளருக்கான குறிப்பு: ரெஸ்பான்டென்டிடம் சொல்லவும்: "இப்போது, நாங்கள் உங்கள் உயரத்தை அளவிட இருக்கிறோம்"**			
8.16	Instructions to the surveyor: Do not read out this question. Fill this yourself.<br>**ஆய்வாளருக்கான குறிப்பு: இந்த கேள்வியை படித்துக்காண்பிக்க வேண்டாம். இதை நீங்களாகவே நிரப்பிக்கொள்வும்.**<br>Is the person being measured:<br>**அளவெடுக்கப்படும் இவர்:**	0 Adult<br>**பெரியவர்**<br>1 Child who can stand with assistance/child who can not stand<br>**தானாக நிற்க முடியாத குழந்தைகள் யாரேனும் ஒருவரது உதவியுடன் நிற்கக்கூடிய ஒர குழந்தை**<br>2 Child who can stand without assistance<br>**யாருடைய உதவியும் இல்லாமல் தனியே நிற்கக்கூடிய ஒரு குழந்தை**	If 0, 2, go to 8.19<br><br>If 1, go to 8.24<br><br>**validate with prefill**: if 0 is selected in 8.01, mem_elig_wom= 1 in the prefill<br>If 1/2 is selected for 8.01, mem_elig_child must be=1 in the prefill<br><br>If there is mismatch, display "incorrect entry. Check again"
8.19	Instructions to the surveyor: Find a flat to keep the stadiometer.	1. Trial 1:<br>**சோதனை** 1:<br>□□□.□ cm	If 2, skip to section 9

	ஆய்வாளருக்கான குறிப்பு: ஸ்டேடியோ மீட்டர் மீட்டரை வைப்பதற்கு உதவியாக சமமானதாக இருக்கும் தரைபரப்பை கண்டறியவும்.<br>Trial 1: Record height in centimeters (nearest to 0.1 cm)<br>சோதனை 1: உயரத்தை சென்டிமீட்டரில் பதிவுசெய்யவும் (0.1 செ.மீ. துல்லியத்திற்கு பதிவு செய்யவேண்டும்)	□□□.□ செ.மீ.<br>2. -98 Refused<br>மறுப்பு தெரிவித்தார்	Restrict between 25-210 cm (including 25 and 210)
30 second break between 8.19 and 8.20			
8.20	Trial 2: Record height in centimeters (nearest to 0.1 cm)<br>சோதனை 2: உயரத்தை சென்டிமீட்டரில் பதிவுசெய்யவும் (0.1 செ.மீ. துல்லியத்திற்கு பதிவு செய்யவேண்டும்)	1. Trial 2:<br>சோதனை 2:<br>□□□.□ cm<br>□□□.□ செ.மீ.<br>2. -98 Refused<br>மறுப்பு தெரிவித்தார்	If 2, skip to 8.22<br><br>Go to 8.21 if difference between 8.19 and 8.20 is more than 0.5 cm), else go to 8.22<br><br>Restrict between 25-210 cm (including 25 and 210)
30 second break between 8.20 and 8.21			
8.21	Trial 3:<br>Record height in centimeters (nearest to 0.1 cm)<br>சோதனை 3: உயரத்தை சென்டிமீட்டரில் பதிவுசெய்யவும் (0.1 செ.மீ. துல்லியத்திற்கு பதிவு செய்யவேண்டும்)	Trial 3:<br>சோதனை 3:<br>□□□.□ cm<br>□□□.□ செ.மீ.<br>-98 Refused<br>மறுப்பு தெரிவித்தார்	Only ask if the difference between 8.20 and 8.19 is more than 0.5 cm)<br><br>Restrict between 25-210 cm (including 25 and 210)
8.22	Instructions to the surveyor: Do not read out this question. Fill this yourself.	0 No<br>இல்லை<br>1 Yes	Skip to Section 9 after this

	ஆய்வாளருக்கான குறிப்பு: இந்த கேள்வியை படித்துக்காண்பிக்க வேண்டாம். இதை நீங்களாகவே நிரப்பிக்கொள்வும்.<br>Was the individual able to stand according to protocol?<br>நெறிமுறைக்கு ஏற்ப இவரால் நிற்க முடிந்ததா?	ஆம்	
8.24	Instructions to the surveyor: Record length using infantometer<br>ஆய்வாளருக்கான குறிப்பு: இன்ஃபேன்டோ மீட்டரை பயன்படுத்தி உயரத்தை பதிவு செய்யவும்<br>Trial 1: Record length in centimeters (nearest to 0.1 cm)<br>சோதனை 1: உயரத்தை சென்டிமீட்டரில் பதிவு செய்யவும் (0.1 செ.மீ. துல்லியத்திற்கு பதிவு செய்யவேண்டும்)	1. Trial 1:<br>சோதனை 1:<br>□□□.□ cm<br>□□□.□ செ.மீ.<br>2. -98 Refused<br>மறுப்பு தெரிவித்தார்	If 2, skip to next section<br><br>Restrict between 25-100 cm (including 25 and 100)
30 second break between 8.24 and 8.25			
8.25	Trial 2: Record length in centimeters (nearest to 0.1 cm)<br>சோதனை 2: உயரத்தை சென்டிமீட்டரில் பதிவுசெய்யவும் (0.1 செ.மீ. துல்லியத்திற்கு பதிவு செய்யவேண்டும்)	1. Trial 2:<br>சோதனை 2:<br>□□□.□ cm<br>□□□.□ செ.மீ.<br>2. -98 Refused<br>மறுப்பு தெரிவித்தார்	If 2, skip to 8.27<br><br>Go to 8.26 if difference between 8.24 and 8.25 is more than 0.5 cm), Else go to 8.27<br><br>Restrict between 25-100 cm (including 25 and 100)

30 second break between 8.25 and 8.26			
8.26	Trial 3: Record length in centimeters (nearest to 0.1 cm)<br>**சோதனை** 3: **உயரத்தை சென்டிமீட்டரில் பதிவுசெய்யவும்** (0.1 **செ.மீ. துல்லியத்திற்கு பதிவு செய்யவேண்டும்**)	1. Trial 3:<br>**சோதனை** 3:<br>□□□.□ cm<br>□□□.□ **செ.மீ.**<br>2. -98 Refused<br>**மறுப்பு தெரிவித்தார்**	Only ask if the difference between 8.24 and 8.25 is more than 0.5 cm)<br><br>Restrict between 25-100 cm (including 25 and 100)
8.27	Instructions to the surveyor: Do not read out this question. Fill this yourself.<br><br>Was the child able to lie down according to protocol?<br>**செயல் முறையின் விதிப்படி குழந்தையால் படுக்க முடிந்ததா** ?	0 No<br>**இல்லை**<br>1 Yes<br>**ஆம்**	

Section 9: Testing for anaemia (Hemocue Test)

பிரிவு 9: இரத்த சோகையை கண்டறிவதற்கான பரிசோதனை (இரத்த பரிசோதனை)

Instructions to surveyors:

ஆய்வாளருக்கான குறிப்புகள்:

Read: This section must be repeated for all women in the household within the target age bracket (12 and 40 years) and for all children with the target age bracket (6 months to 59 months) in the presence of their mothers.

படித்துக்காண்பிக்கவும்: 12 முதல் 40 வயதுக்குட்பட்ட அனைத்து வீட்டுப்பெண்களுக்கும், 6 மாதங்கள் முதல் 59 மாதங்களுக்குட்பட்ட அனைத்து குழந்தைகளுக்கும் இந்த பிரிவிலுள்ள கேள்விகளை அவர்களது அம்மாவின் முன்நிலையில் கேட்கவேண்டும்.

Now we would like test you/your child for anemia. Anemia is a condition where the amount of hemoglobin in the blood is lower than normal. Hemoglobin is an important part of the blood and we need enough of it to conduct our daily activities

and to stay healthy. Anemia can be caused by many things, including poor nutrition and infections. It can result in weakness and fatigue, higher risk of getting sick, developmental delays and complications during pregnancy.

இப்போது, உங்களுக்கும்/ உங்கள் குழந்தைக்கும் இரத்த சோகை நோய் உள்ளதா என்று கண்டறிய நாங்கள் பரிசோதனை செய்ய போகிறோம். இரத்த சோகை எனில் இரத்தத்தில் காணப்படும் ஹீமோகுளோபினின் அளவு இயல்பைவிட குறைவாக காணப்படுவதாகும். ஹீமோகுளோபின் என்பது இரத்தத்தில் காணப்படும் ஒரு முக்கிய பொருளாகும், மேலும் நாம் நமது தினசரி வேலைகளை செய்வதற்கும் மற்றும் ஆரோக்கியமாக இருப்பதற்கும் இதை நாம் அதிகம் பெற்றிருக்கவேண்டும். இரத்த சோகை ஊட்டச்சத்து குறைபாடு மற்றும் நோய் தொற்று போன்றவை உள்ளடக்கிய பல காரணங்களால் ஏற்படக்கூடும். மேலும் இது சோர்வையும், களைப்பையும் மற்றும் நோய் வயப்படுவதற்கான அதிக ஆபத்துக்களையும், வளர்ச்சி குறைபாட்டையும் மற்றும் கர்பகால சிக்கல்களையும் ஏற்படுத்தும்.

To do this, we'll need to obtain a few drops of blood from a small prick and we'll use a machine to tell if you have anemia. This prick should hurt very little and any pain will quickly go away. This will not pose any risk to you. We will give you the results of the test and tell you if there is any serious issue. If you have any questions about your results please ask a medical provider.

இந்த பரிசோதனையை செய்ய உங்கள் கைவிரலில் ஒரு சிறிய ஊசி மூலம் குத்தி ஒரு சில சொட்டு இரத்தத்தை எடுத்து அதை ஒரு கருவியில் செலுத்தி உங்களுக்கு இரத்த சோகை இருக்கிறதா என்று நாங்கள் கண்டறிவோம். இப்படி ஊசி மூலம் குத்துவது உங்களுக்கு கொஞ்சமாக வலியை ஏற்படுத்தலாம், ஆனாலும் அந்த வலி மிக விரைவாகவே நீங்கிவிடும். இதனால் உங்களுக்கு எந்த ஆபத்தும் ஏற்படாது. இந்த பரிசோதனையின் முடிவை கண்டறிந்து உங்களுக்கு ஏதேனும் அபாயகரமான பிரச்சனை இருந்தால் நாங்கள் அதை உங்களிடம் தெரிவிப்போம். ஒருவேளை உங்கள் பரிசோதனை முடிவில் உங்களுக்கு ஏதேனும் சந்தேகம் ஏற்பட்டால் அதைக்குறித்து நீங்கள் மருத்துவரிடம் கேட்டு தெளிவுபெறலாம்.

If the respondent appears reluctant to participate in the test, remind them of the following:

ஒருவேளை இந்த பரிசோதனையில் கலந்துக்கொள்ள பங்குகொள்ளும் நபர் தயக்கம் காட்டினால் அவருக்கு பின்வரும் இந்த விஷயங்களை நினைவுப்படுத்தவும்:

1. The test is completely safe and hygienic.
   இந்த பரிசோதனை முற்றிலும் பாதுகாப்பானதும் சுகாதாரமானதும் ஆகும்.
2. The pain from the blood prick is very small and will quickly go away.

இரத்தம் எடுக்க ஊசி குத்துவதால் கொஞ்சமாகவே வலி ஏற்படும், ஆனாலும் அது விரைவிலேயே சரியாகிவிடும்

3. The value of this test (if purchased) is at least 100 rupees, but we are offering it for free.
   இந்த பரிசோதனையை நீங்கள் வெளியே சென்று செய்துக்கொண்டால் அதற்கு குறைந்தது 100 ரூபாய் செலவாகும், ஆனால் நாங்கள் அதை உங்களுக்கு இலவசமாகவே செய்கிறோம்.

4. The respondent's participation will be helpful to us in better understanding health conditions in Tamil Nadu.
   நீங்கள் இதில் பங்குகொள்வதால் தமிழகத்திலுள்ள பெண்களின் உடல் நிலையை அறிந்துக்கொள்வதற்கு எங்களுக்கு மிகவும் உதவியாக இருக்கும்.

If the respondent is still reluctant, DO NOT pressure them to participate. You must be calm and polite.

பங்குகொள்பவர் இன்னும் தயக்கம் காட்டியபடியே இருந்தால், பங்குகொள்ளும்படி அவரை மேலும் வற்புறுத்தவேண்டாம். அமைதியாக அவர் விருப்பப்படியே விட்டுவிடவும்.

Instruction to data team: Repeat Section 9 and 10 for ever eligible member in the hh (i.e.- mem_elig_child OR mem_elig_woman is 1), who is still living in the hh (i.e.- H0.12=1) AND If they are actually eligible (H0.13=1)

**Q. No. கே.எண்**	**Question கேள்வி**	**Answer Options பதிலுக்கான தேர்வுகள்**	**Skip Pattern**
Instruction to the data team:<br>Display:<br>Name of the member<br>குடும்ப உறுப்பினர் பெயர்<br>ID of the member<br>உறுப்பினரின்அடையாளஎண்			
**9.00**	Is this person available for the survey right now?<br><br>இப்பொழுது இந்த நபர் சர்வேக்கு வீட்டில் இருக்கிறாரா?	0 No<br>இல்லை<br>1 Yes<br>ஆம்	**If 0 then go to the next person in the roster.**

Q. No.<br>கே. எண்	Question<br>கேள்வி	Answer Options<br>பதிலுக்கான தேர்வுகள்	Coding Instructions
9.01A	Hemocue measurement<br>ஹீமோகுளோபின் அளவு<br><br><u>Instructions to the surveyor:</u><br>Do not read out this question. Fill this yourself.<br>ஆய்வாளருக்கான குறிப்பு: இந்த கேள்வியை படித்துக்காண்பிக்க வேண்டாம். இதை நீங்களாகவே நிரப்பிக்கொள்வும்	1. Finger prick<br>விரல்நுனியில்<br><br>2. Feet prick<br>பாதத்தில்	
9.02	Hemocue measurement<br>ஹீமோகுளோபின் அளவு<br><br>**<u>Instructions to surveyor:</u>**<br>Record Hemoglobin concentration to the nearest 0.1 g/dL)<br>**<u>ஆய்வாளருக்கான குறிப்பு:</u>**<br>ஹீமோகுளோபின் அடர்த்தியை 0.1 g/dL துல்லியத்திற்கு பதிவு செய்யவும்<br><br>Refer to a doctor if Hb level is less than 7g/dL for pregnant women and children and less than 8g/dL for other women<br>Hb நிலை கர்ப்பிணி பெண்களுக்கும் குழந்தைகளுக்கும் 7g / dL க்கும் குறைவாக இருந்தால், மற்ற பெண்களுக்கு 8g / dL க்கும் குறைவாக இருந்தால் ஒரு	☐☐.☐ g/dL<br>-98 Refused<br>மறுப்பு தெரிவித்தார்	Allow values between 0 to 25, including 0. Values should have 1 number after the decimal point.<br><br>Skip to Section 10 if mem_elig_child is 1 for this person.

	மருத்துவரைப் பார்க்க பரிந்துரைக்கவும்		
9.03	Have you used any cigarettes or beedis in the last 30 days?<br>கடந்த 30 நாட்களில் நீங்கள் ஏதேனும் பீடியோ சிகரெட்டோ பிடித்தீர்களா?	0 No<br>இல்லை<br>1 Yesஆம்<br>-98 Refused<br>மறுப்பு தெரிவித்தார்	If No, skip to section 10
9.04	Approximately how many did you smoke in the last 24 hours?<br>கடந்த 24 மணி நேரத்தில் தோராயமாக எத்தனை பீடி அல்லது சிகரெட்டை நீங்கள் பிடித்தீர்கள்?	□□<br>-98 Refused<br>மறுப்பு தெரிவித்தார்<br>-99 Don't know<br>தெரியாது	Restrict numeric entry to integer, 0-100, not including 0 but including 100

Section 10: DBS Testing

பிரிவு 10: DBS பரிசோதனை

Instruction to data team: No need to show all that is written below on the tablet. In this section, the tablet should directly go to the questions starting from 10.00.

தரவுக்குழுவுக்கான குறிப்பு: **கீழே கொடுக்கப்பட்டள்ள அனைத்தையும் டேப்ளட்டின் திரையில் காண்பிக்க வேண்டிய அவசியமில்லை**. **இந்த பிரிவு டேப்ளட்டில் வரும் போது நேரடியாக** 10.00 **கேள்வியிலிருந்து துவங்கினால் மாத்திரமே போதுமானதாகும்**.

Interviewer read: Now I would like to take a sample of your blood. This will allow us to understand your health.

ஆய்வாளர் பங்குகொள்ளும் நபரிடம் படித்துக்காண்பிக்க வேண்டியது: இப்போது, உங்கள் இரத்த மாதிரியை நான் எடுக்க இருக்கிறேன். இதன் மூலம் உங்கள் உடல் ஆரோக்கியத்தை குறித்து எங்களால் அறிந்துக்கொள்ள முடியும்.

Interviewer read: Let's sit. What I will do is make a small prick to your finger, then place the blood onto this card, taking only a few drops. Would you prefer that I use your right or left hand?

ஆய்வாளர் பங்குகொள்ளும் நபரிடம் படித்துக்காண்பிக்க வேண்டியது: நாம் உட்கார்ந்துக்கொள்வோமா. இப்போது, நான் உங்கள் விரலுநுனியில் ஒரு சிறிய ஊசி மூலம் குத்தி அதிலிருந்து வரக்கூடிய இரத்தத்தில் ஒரு சில துளிகளை மட்டுமே இந்த அட்டையில்

தடவிக்கொள்வேன். இப்போது ஊசியை வலதுக்கைவிரலில் குத்தலாமா அல்லது இடதுக்கைவிரலில் குத்தலாமா?

Interviewer flag: Look for skin without damage and calluses on the respondent's chosen hand. Middle or ring fingers are best.

ஆய்வாளருக்கான குறிப்பு: பங்குகொள்பவர் சொன்ன கைவிரலில் தோலில் ஏதேனும் பாதிப்போ அல்லது சிராப்போ இருக்கிறதா என்று பார்க்கவும். இதற்கு மோதிர விரல் அல்லது நடுவிரல் தான் சிறந்தது.

Interviewer flag: Wash your hands and put on your disposable gloves. Clean the respondent's finger with an alcohol pad and allow the alcohol to dry completely.

ஆய்வாளருக்கான குறிப்பு: உங்கள் கைகளை கழுவிக்கொண்டு கைகளுக்கு ஒருமுறை பயன்படுத்தப்படும் கையுறையை அணிந்துக்கொள்ளவும். பங்குகொள்பவரின் கைவிரலில் ஆல்கஹால் தடவிக்கொண்டு அதை நன்றாக உலர விடவும்.

Interviewer read: Please rub your hands together for a few moments to warm them. Thank you. Now I will make a small prick to your finger. This will require me to put some gentle pressure on your finger.

ஆய்வாளர் பங்குகொள்ளும் நபரிடம் படித்துக்காண்பிக்க வேண்டியது: இப்போது, உங்கள் இரண்டு உள்ளங்கைகளையும் ஒன்றோடு ஒன்று வைத்து நன்றாக தேய்த்து சூடு உண்டாக்கவும். நன்றி. இப்போது, நான் உங்கள் விரலில் ஊசியை கொண்டு லேசாக குத்துவேன். இதற்காக நான் உங்கள் விரலில் சிறிது அழுத்தம் கொடுக்க வேண்டியதிருக்கும்.

Interviewer flag: Place a disposable cloth in your lap. Make sure the respondent's hand is positioned below the level of her heart. Grasp the chosen finger between your thumb and index finger. Gently milk the chosen finger, and squeeze it gently in preparation. Push the lancet cap in and remove it by twisting.

ஆய்வாளருக்கான குறிப்பு: உங்கள் மடியில் ஒரு முறை பயன்படுத்தப்படும் ஒரு துணியை வைத்துக்கொள்ளவும். இப்போது பங்குகொள்பவரின் கைவிரல் அவரது இதயத்திற்கு கீழாக இருக்கும்படி வைக்கப்பட்டிருக்கிறதை உறுதி செய்துக்கொள்ளவும். இப்போது பங்குகொள்ளும் நபரின் விரலை உங்கள் கட்டைவிரல் மற்றும் ஆள்காட்டி விரலால் பிடித்துக்கொள்ளவும். பங்குகொள்ளும் நபரின் அந்த விரலை தயார் படுத்தும் காரணத்திற்காக மென்மையாக உருவவும். லேன்சன்ட் கேப்பை உள்ளே தள்ளி அதை திருகி அகற்றவும்.

Interviewer flag: Prick the side of the finger farther from the thumb, near the tip, with the lancet. Wipe away the first drop of blood with a sterile gauze pad. Holding the subject's hand downward, gently milk the hand starting at the wrist, and working down to the base of the finger. Do not squeeze the finger. Once a large drop is hanging, allow it to drop onto the collection card. You may touch the card to the drop but do not touch the card directly against the finger.

ஆய்வாளருக்கான குறிப்பு: இப்போது, தேர்வு செய்யப்பட்டுள்ள அந்த விரலில் பக்கவாட்டு நுனியில் கட்டை விரலிலிருந்து தள்ளி லேன்சன்ட் மூலம் குத்தவும். வெளியே வரும் முதல் சொட்டு இரத்தத்தை ஸ்டெரெய்ல் காஜ் பேட் கொண்டு துடைத்து எடுத்துவிடவும். இப்போது பங்குகொள்பவரின் கைகளை கீழ்நோக்கியபடி பிடித்துக்கொண்டு மணிக்கட்டிலிருந்து துவங்கி விரல்நுனி வரை மென்மையாக உருவவும். ஆனால் விரலை கசக்க வேண்டாம். இப்போது அவரது விரல்நுனியில் ஒரு பெரிய சொட்டு இரத்தம் காணப்படும்போது, அதை சேகரிப்பு அட்டையில் விழும்படி செய்யவும். அட்டையை அந்த சொட்டு இரத்தத்தின் மீது படும்படி நீங்கள் வைக்கலாம் ஆனாலும் அட்டையை விரல் மீது படும்படி வைக்கக்கூடாது.

Interviewer flag: Attempt to fill five full circles on the collection card, one drop per circle, then stand it onto a disposable cloth to dry. Once finished clean the subject’s finger and place a bandage onto the site of the prick. If necessary, ask permission to prick a second finger and repeat the procedure. Dispose of all materials properly.

ஆய்வாளருக்கான குறிப்பு: அந்த அட்டையில் இரத்தத்தின் ஐந்து முழு வட்டங்களை உருவாக்கிக்கொள்ளவும். ஒரு சொட்டிற்கு ஒரு வட்டம். பிறகு அந்த அட்டையை ஒருமுறை பயன்படுத்தப்படும் துணியின் மீது வைத்து உலரவிடவும். அது உலர்ந்ததும் பங்குகொள்பவரின் விரலை சுத்தம் செய்து ஊசி குத்தப்பட்டுள்ள அந்த இடத்தின் மீது ஒரு பேன்டேஜை போட்டுவிடவும். தேவையெனில் இரண்டாவது விரலில் குத்தி இரத்தமெடுக்க அவரிடம் அனுமதி கேட்டு, இந்த செய்முறையை மீண்டும் செய்யவும். இறுதியில் பயன்படுத்திய எல்லா பொருட்களையும் அகற்றிவிடவும்.

Instruction to data team:

Show this section for a person if the following criteria is met:

1. Eligible member (in pre-fill: mem_elig_child OR mem_elig_wom is 1)

2. Still living in the hh (H0.12=1)

3. from DBS household (section10_dbs=1 for the ID entered in H0.07A OR section10_dbs=1 for the ID entered in H2.02A)

4. Selected for DBS (in pre-fill: dbs_child OR dbs_wom is 1)

5. If they are actually eligible (H0.13=1)

Q. No.<br>கே. எண்	Question<br>கேள்வி	Answer Options<br>பதிலுக்கான தேர்வுகள்	Coding Instructions
Instruction to the data team:			

Display:<br>Name of the member<br>குடும்ப உறுப்பினர் பெயர்<br>ID of the member<br>உறுப்பினரின்அடையாளஎண்			
10.0A	Is this person available for the survey right now?<br><br>இப்பொழுது இந்த நபர் சர்வேக்கு வீட்டில் இருக்கிறாரா?	0 No இல்லை<br><br>1 Yes ஆம்	If 0 then go to the next person in the roster.
10.00	Did you make an attempt to take a DBS sample from [name]?<br>Instruction to enumerator: Don't ask the respondent.<br>நீங்கள் DBS மாதிரியை [பெயர்] யிடம் எடுக்க முயற்சித்தீர்களா?<br>ஆம் எனில் , அவகள் / அவற்றின் பாதுகாவலர் ஒப்புதல் அளித்தீர்களா?<br>ஆய்வாளருக்கான குறிப்பு:<br>பங்குபெறுபவரிடம் கேட்க வேண்டாம்	1. Tried to get the DBS sample but respondent refused. இரத்த மாதிரி எடுக்க முயற்சி செய்தோம் ஆனால் மறுப்புதெரிவித்துவிட்டார்கள்<br>2. Tried to take DBS sample but could not get enough blood drops முயற்சி செய்த பின்னுப்பும் இரத்த மாதிரியை எடுக்க முடியவில்லை<br>3. Successfully took the DBS sample இரத்த மாதிரிகள் எடுக்க பட்டது<br>4.	If 1, skip to 10.03<br><br>If 2,3, go to 10.01
10.01	How many eligible/ complete blood spots were collected?<br>எத்தனை இரத்த வட்டங்கள் சேகரிக்கப்பட்டது<br>Instruction to enumerator: Don't ask the respondent.<br>ஆய்வாளருக்கான குறிப்பு:	___ number of eligible/ complete blood spots<br>___ தகுதியான ரத்த வட்டங்களின் எண்ணிக்கை	Numeric entry between 0-5, including 0 and 5.<br><br>If entry between 0-2 and selected '3' in 10.00 then show error message

	பங்குபெறுபவரிடம் கேட்க வேண்டாம்.		“Wrong entry” and don’t let the questionnaire move forward.<br><br>If entry between 3-5 and selected ‘2’ in 10.00 then show error message “Wrong entry” and don’t let the questionnaire move forward.
10.02	How many pricks did you take to collect the blood spots?<br>இரத்த துளிகள் எடுப்பதற்கு எத்தனை முறைகள் ஊசி குத்தப்பட்டது?<br>Instruction to enumerator: Don’t ask the respondent.<br>ஆய்வாளருக்கான குறிப்பு: பங்குபெறுபவரிடம் கேட்க வேண்டாம்.	-- times<br>முறைகள்	Numeric entry less than 4 and greater than 0, not including 0
10.02A	Why did you not prick 3 times?<br>எதனால் 3 முறை ஊசி குத்த உங்களால் முடிய வில்லை?	1. . Child / women was uncooperative<br>குழந்தை/ பெண்கள் ஒத்துழைக்க முடியவில்லை?<br>2. Parent/guardian refused<br>பெற்றோர்/ பாதுகாவலர் மறுத்து விட்டார்<br>3. Others: Specify<br>மற்றவை: குறிப்பிடவும்	Only ask if 10.00 is ‘2’ AND 10.02 is less than 3.

10.03	Comments<br>குறிப்புகள்		Optional

Instruction to the surveyor: Please write down the following information on the measurements card and give it to the woman/child.

ஆய்வாளருக்கான குறிப்பு: அளவீடு எழுதும் அட்டையில் கீழ்க்கண்ட தகவலை எழுதி, அதை பெண்மணி / குழந்தைக்கு(குழந்தையின் பெற்றோரிடம்) கொடுக்கவும்

Instruction to data team: Display a table with the following information

Name (from the pre-fill)

Age (from the pre-fill)

Weight :

- from 8.04 if 0/2 is selected in 8.01
- from 8.10c) if 1 is selected in 8.01

Height

- from 8.19 if 0/2 is selected in 8.16
- from 8.24 if 1 is selected in 8.16

Hb

- from 9.02

10.04	Is this form complete? இந்த படிவம் நிறைவடைந்துவிட்டதா?	0. No இல்லை<br>1. Yes ஆம்	Go to 10.04A if '0'<br>Go to 10.05 if '1'
10.04A Instruction to data team: Display the following message on screen and don't allow the surveyors to move forward.<br><br>**Press the back button on the bottom of the tablet, select 'save changes', and move to the next household. If the appointment is on the same day, keep a note of this and revisit the household on the same day.**<br><br>**டேப்ளட்டின் கீழ்ப்புறத்தில் காணப்படும் 'back' பொத்தானை அழுத்தவும், 'save changes' -யை தேர்வை செலக்டு செய்துவிட்டு அடுத்த வீட்டிற்கு நேராக கடந்து செல்லவும். ஒருவேளை ஒதுக்கப்படும் சந்திப்பு நேரம் அதே நாளில் இருக்குமெனில், அதை குறிப்பெடுத்து வைத்துக்கொண்டு அந்த வீட்டை அதே நாளில் திரும்ப வந்து சந்திக்கவும்.**			
10.05	Overall comments		

	முழு ஆய்வுக்கான குறிப்புகள்		

END OF QUESTIONNAIRE 2

கேள்விப்பட்டியல் 2 முடிவுற்றது
